# Protonation‐Gated Orthogonal Control of Highly Efficient Molecular Motors With Broad Visible‐Light Responsiveness

**DOI:** 10.1002/anie.4214875

**Published:** 2026-07-17

**Authors:** Jinghao Wang, Alexander Ryabchun, Ben L. Feringa

**Affiliations:** ^1^ Stratingh Institute for Chemistry University of Groningen Groningen the Netherlands

**Keywords:** adaptive molecular machines, broad‐visible‐spectrum excitation, molecular motors, multi‐stimulus‐responsive systems, protonation‐gated switching

## Abstract

Molecular motors with red‐shifted absorption are highly attractive for biomedical applications, yet remain challenging to realize because conventional strategies for extending excitation wavelengths often compromise photochemical efficiency and directional motor function. Here, we report a protonation‐gated strategy in which reversible modulation of conjugation topology enables state‐dependent molecular motor operation across a broad visible‐light window. Modifying coumarin‐derived overcrowded alkenes with benzazole substituents, the resulting motors undergo efficient photoisomerization under blue–green light irradiation. Photophysical studies supported by DFT calculations reveal that protonation induces a pronounced conformational inversion with the formation of intramolecular hydrogen bonds, thereby promoting molecular planarization and enhanced electronic delocalization, leading to the efficient photoisomerization under 530–600 nm irradiation while quantitative photoconversion is retained. Overall, these findings establish reversible protonation as a powerful chemical gating strategy to expand the visible‐light responsiveness of the motor without permanent structural modification, paving the way for its application in multimodal responsive bioimaging and advanced optical devices.

## Graphical abstract

Reversible protonation gates coumarin‐based overcrowded‐alkene molecular motors by dynamically reshaping their conjugation topology. This strategy enables efficient, near‐quantitative unidirectional photoisomerization from blue–green to red‐shifted visible light up to 600 nm, while preserving motor function and allowing orthogonal light, heat, and acid/base control in a polymer matrix.

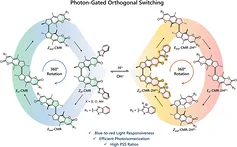

## 1 Introduction

Light‐driven molecular motors represent one of the most advanced classes of artificial molecular machines, capable of converting light energy into controlled unidirectional motion at the nanoscale and thereby offering broad potential toward dynamic surfaces, adaptive catalysts, and responsive materials [1, 2, 3, 4, 5, 6, 7, 8, 9, 10]. Among these practical applications, achieving efficient operation under visible light irradiation remains particularly important, as ultraviolet excitation often induces photodegradation and limited compatibility in biological systems and functional materials [11, 12, 13, 14]. Since the pioneering development of overcrowded‐alkene‐based motors, significant efforts have been devoted to improving their photochemical efficiency, spectral tunability, and operational controllability [15, 16, 17]. Current strategies to red‐shift motor absorption mainly rely on either molecular structure modifications, through π‐conjugation extension, donor–acceptor engineering, or using external photosensitizers [18, 19, 20, 21, 22, 23, 24, 25]. Although these strategies have achieved longer‐wavelength responsiveness in some cases, they often compromise with photochemical efficiency or directional fidelity [26, 27, 28, 29]. More importantly, most existing systems operate within a relatively narrow spectral window, preventing the increasing demands for applications across different regions of the visible spectrum [30, 31]. As a result, a general approach that enables reversible tailored wavelength activation while preserving efficient motor function remains highly desirable.

In contrast, chemical‐gated light‐responsive systems provide an attractive alternative because they enable in situ control over molecular function through external chemical inputs within a single architecture [32, 33, 34, 35, 36, 37, 38, 39, 40]. Among such inputs, protonation is particularly appealing because it can reversibly reconfigure conjugation, charge distribution, and excited‐state energetics in molecules, thereby providing a powerful means to directly tune wavelength responsiveness without permanently altering the molecular framework [41, 42, 43, 44, 45]. However, integrating proton‐induced switching with efficient motor function remains challenging because structural perturbations potentially influence the overcrowded‐alkene stability and disrupt the delicate balance between photoisomerization and thermal helix inversion processes [33].

**FIGURE 1:**
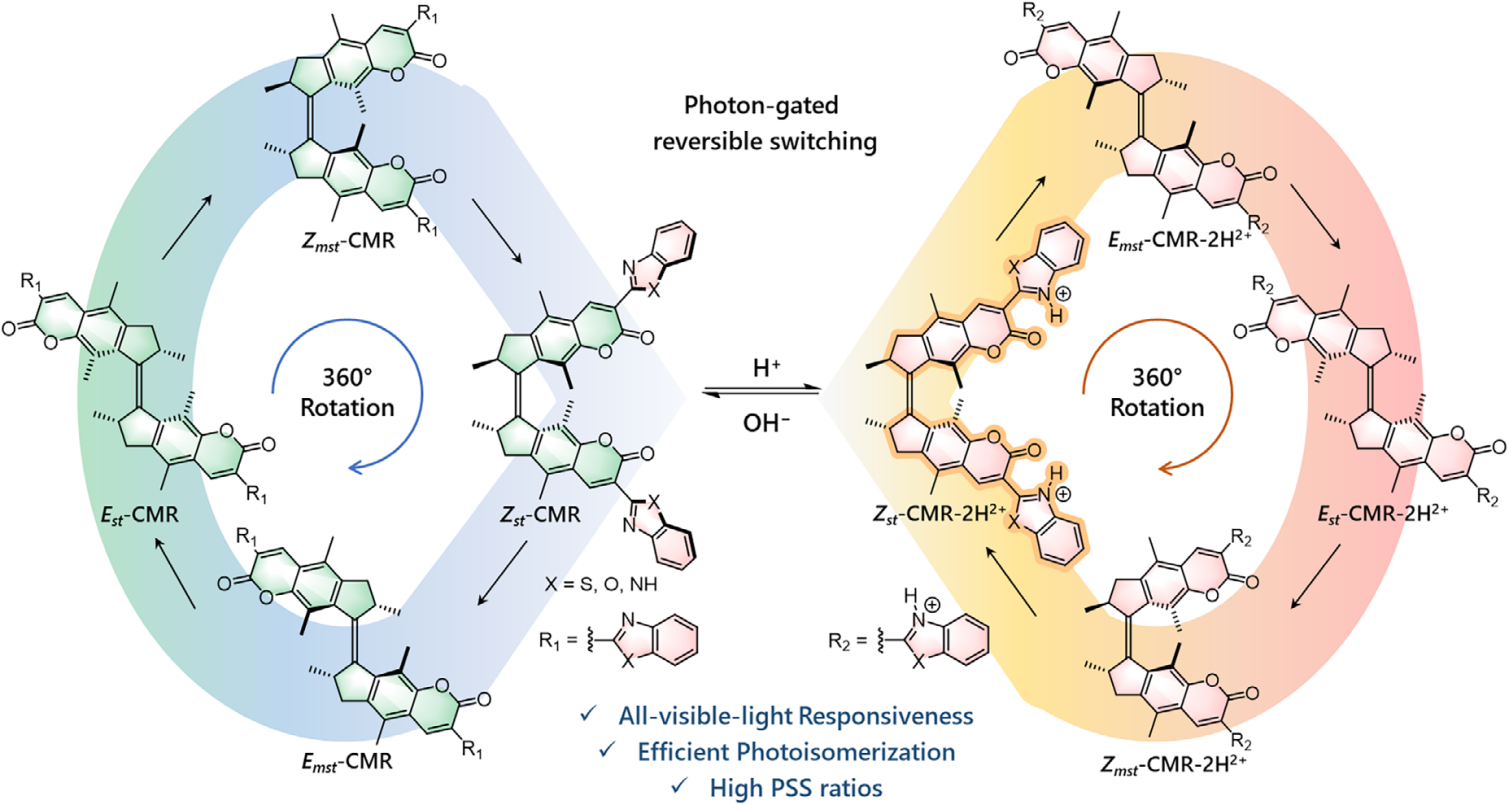
Schematic illustration of the protonation‐gated orthogonal control of molecular motors' rotation with broad visible‐light responsiveness. Left: four‐state rotary cycle of the motors in the neutral states under blue‐green light irradiation; Right: four‐state rotary cycle of the motors in the protonated states under yellow–red light irradiation. The subscript “*st*” indicates a stable state, and “*mst*” means metastable state.

Herein, we report a protonation‐gated molecular motor platform in which reversible modulation of conjugation topology enables state‐selective access to the broad visible‐light excitation window with high efficiency. By combining a coumarin‐derived motor core with nitrogen‐containing benzazole substituents, we designed motors whose neutral and protonated forms exhibit distinct conformational and electronic structures. In the neutral state, these coumarin‐fused molecular motors undergo unidirectional rotation with near‐quantitative photoconversion under blue–green light irradiation (Figure 1, left). Upon protonation process, the motors displayed a substantial bathochromic shift in absorption maximum of up to 100 nm, while showing no fatigue during multiple cycles of protonation/deprotonation. Further computational calculations indicate a pronounced conformational reorganization that promotes planarization and enhanced electronic delocalization of the structures. Importantly, the unidirectional light‐driven rotation of these protonated motors is retained, enabling quantitative photoconversion and efficient operation at substantially longer wavelengths in the visible region, allowing up to 600 nm irradiation (Figure 1, right). Further incorporation of the motors into a polymer matrix reveals reversible multimodal control under orthogonal light, heat, and proton stimuli. Overall, this gating mechanism establishes a general strategy for controlling motor rotation with light across essentially the blue‐to‐red visible range without covalent modification of the motor scaffold. By dynamically reprogramming electronic structure rather than permanently modifying molecular architecture, this work provides a new design principle for adaptive molecular machines capable of broad visible‐light responsiveness and stimulus‐controlled function.

## 2 Results and Discussion

### 2.1 Synthesis and Unidirectional Rotary Cycle of Motors in a Neutral State

The target molecular motors were designed by functionalization of the “first‐generation motors” scaffold through a Knoevenagel condensation, affording the formation of the coumarin‐fused motors containing benzazole substituents, which could undergo a reversible protonation process under acid‐base cycles (Figure 1) [35, 46, 47]. To investigate the influence of the different heteroatoms in the benzazole units on the photophysical and motor properties, three derivatives incorporating benzothiazole (**BZT**, **X** = **S**), benzoxazole (**BZO**, **X** = **O**), and benzimidazole (**BID**, **X** = **NH**) were synthesized. The detailed synthetic route is summarized in the Section S3. Unless otherwise noted, all experiments in this study were performed with the (***S***, ***S***)‐(***M***, ***M***)‐***Z_st_*** enantiomer. For clarity, **CMR** is used as a general abbreviation for the coumarin‐fused motor scaffold across the series, and the stable isomer (***S***, ***S***)‐(***M***, ***M***)‐***Z_st_***‐**CMBZT** is hereafter abbreviated as ***Z_st_***‐**CMBZT**, in the same way for ***Z_st_***‐**CMBZO** and ***Z_st_***‐**CMBID**. All the novel structures were fully characterized by ^1^H and ^13^C‐NMR spectroscopy as well as high‐resolution electrospray ionization mass spectrometry (Sections S15 and S16).

**FIGURE 2:**
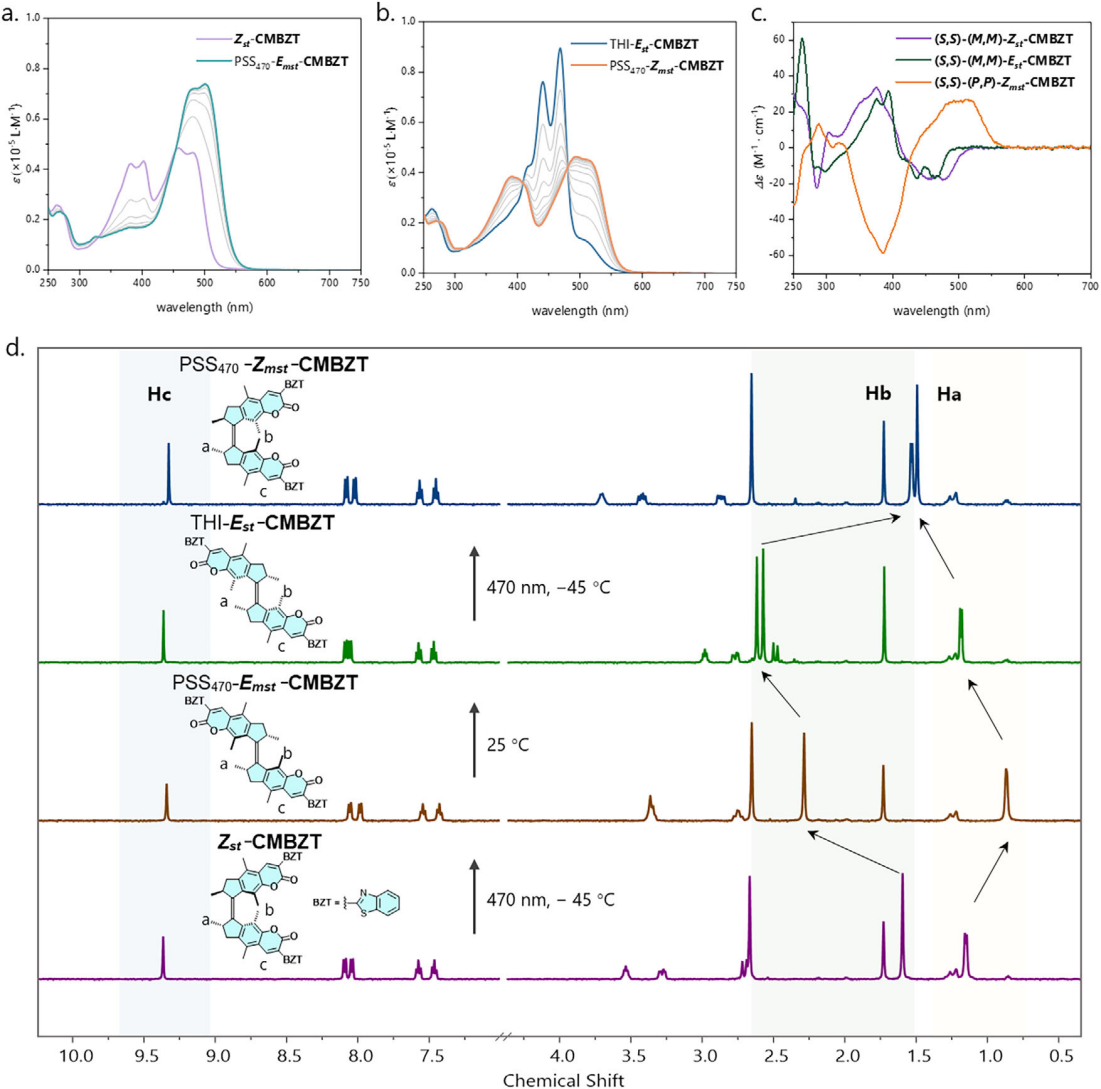
Rotary cycle of the **CMBZT** via UV–vis, ECD, and ^1^H‐NMR spectroscopies. (a) Changes in the UV–vis absorption spectra of ***Z_st_***‐**CMBZT** (purple line; THF, −12 °C, 15 µM) upon irradiation at 470 nm to generate PSS_470_‐***E_mst_***‐**CMBZT** (green line), recorded every 5s (grey lines, indicating the intermediate spectra). (b) Changes in the UV–vis absorption spectra of ***E_st_***‐**CMBZT** (blue line; THF, −12 °C, 15 µM) upon irradiation at 470 nm light to generate PSS_470_‐***Z_mst_***‐**CMBZT** (orange line), recorded every 5s (grey lines, indicating the intermediate spectra).(c) ECD spectra starting from (*S*,*S*)‐(*M*,*M*)‐***Z_st_***‐**CMBZT** (purple line, THF), followed by 470 nm light irradiation at –12°C of the sample and then heating the solution to room temperature to obtain (*S*,*S*)‐(*M*,*M*)‐***E_st_***‐**CMBZT** (green line) and subsequent 470 nm irradiation to generate (*S*,*S*)‐(*P*,*P*)‐***Z_mst_***‐**CMBZT** (orange line). (d) Rotary cycle of motor **CMBZT** followed by ^1^H NMR spectroscopy (500 MHz, 2 mM in 0.5 mL CD_2_Cl_2_) starting from ***Z_st_***‐**CMBZT** (bottom purple line) to PSS_470_‐***E_mst_***‐**CMBZT** (brown line) under 470 nm light irradiation at –45°C, followed by heating to room temperature for THI to generate THI‐***E_st_***‐**CMBZT** (green line); and subsequent irradiation under 470 nm light at –45°C to PSS_470_‐***Z_mst_***‐**CMBZT** (blue line). All ^1^H NMR measurements were performed at –45 °C to ensure the chemical shifts were not influenced by temperature. Arrows were used to direct attention to the changes in the chemical shifts (*δ*, ppm) of the characteristic resonances.

To investigate the photophysical properties and unidirectional four‐step rotary cycle of these new motors in the neutral state, UV–vis absorption and electronic circular dichroism (ECD) spectroscopy, as well as in situ NMR irradiation experiments were performed to identify the processes of sequential photoisomerization and thermal helix inversion (THI) steps, namely, ***Z_st_*** → ***E_mst_*** → ***E_st_*** → ***Z_mst_*** → ***Z_st_*** (Figure 1). Because the neutral state transformations of the three motors were similar, we mainly discuss the spectral behavior of ***Z_st_***‐**CMBZT** here, while using the appropriate LED wavelength for each sample. Initially, upon irradiation of ***Z_st_***‐**CMBZT** using 470 nm light at −12°C, the characteristic absorption band of ***Z_st_***‐**CMBZT** at 350–400 nm decreased, and a new absorption band at 470–540 nm appeared, displaying a clear isosbestic point at 452 nm, which indicates a selective photochemical interconversion of ***Z_st_***‐**CMBZT** to the corresponding photostationary state (PSS), named PSS_470_‐***E_ms__t_***‐**CMBZT** (Figure 2a). Subsequently, keeping the solution in the dark at −12°C resulted in the THI process with the formation of THI‐***E_st_***‐**CMBZT** (Figure S2b). Following the THI process, irradiation of THI‐***E_st_***‐**CMBZT** induced the second photochemical isomerization, accompanied by a bathochromic absorption maximum from 470 to 510 nm, consistent with the generation of PSS_470_‐***Z_ms__t_***‐**CMBZT** (Figure 2b). Finally, after warming the solution at 328 K for 5 h, nearly identical UV–vis spectra were obtained as the initial isomer with the absorption maximum at 470 nm, indicating the THI process from PSS_470_‐***Z_ms__t_***‐**CMBZT** to THI‐***Z_s__t_***‐**CMBZT** (Figure S2d) [48]. The rotary cycle was further supported by ECD spectroscopy. The initial (***S***, ***S***)‐(***M***, ***M***)‐***Z_st_***‐**CMBZT** motor exhibited a negative band ranging from 400 to 500 nm and a positive peak at 350 nm (purple line, Figure 2c). Upon sequential irradiation and warming, the ECD spectrum underwent a gradual hypochromic shift and decrease in intensity, reflecting the formation of (***S***, ***S***)‐(***M***, ***M***)‐***E_st_***‐**CMBZT** isomer (green line, Figure 2c). Further irradiation resulted in an inversion of the ECD signals with a positive band at ∼520 nm and a negative signal at ∼380 nm, which can be assigned to the formation of the (***S***, ***S***)‐(***P***, ***P***)‐***Z_mst_***‐**CMBZT** diastereomers (orange line, Figure 2c).

Further ^1^H‐NMR experiments with in situ irradiation also demonstrated the unidirectional motor rotation. Irradiation of the isomer ***Z_st_***‐**CMBZT** with 470 nm light at –45°C led to nearly quantitative isomerization toward the corresponding metastable isomer, PSS_470_‐***E_ms__t_***‐**CMBZT**, as evidenced by an upfield shift of the allylic methyl signals and a downfield shift of the benzylic methyl signals (**Ha** and **Hb**, respectively, Figure 2d). Subsequent warming of the sample to 25°C resulted in the THI process with the full conversion to the thermodynamically stable ***E_st_***‐**CMBZT** isomer, which was characterized by the downfield shift of both the allylic and benzylic methyl protons (Figure 2d). Further irradiation afforded ***Z_mst_***‐**CMBZT** in near‐quantitative yield, followed by a second THI step with the regeneration of ***Z*_st_**‐**CMBZT**, thus accomplishing a complete 360° unidirectional rotation with excellent selectivity, as reflected by the quantitative photoconversion (Figure S31). The integrations of the characteristic ^1^H‐NMR signals reveal the high PSS ratios for both photochemical steps, reaching 98:2 for the ***Z_st_***‐**CMBZT** to ***E_ms__t_***‐**CMBZT,** and 99:1 for the ***E_s__t_***‐**CMBZT to *Z_ms__t_***‐**CMBZT**, respectively (Table 1 and Figure S32). Comparable behavior was observed for **CMBZO** and **CMBID** under 455 nm irradiation, both of which exhibit analogous UV–vis spectral evolution and undergo efficient unidirectional rotary cycles with similarly high PSS ratios reaching well beyond 95:5 (Table 1 and Figures S3, S4, and S33–S36).

**TABLE 1:** Gibbs free energy of activation (Δ^‡^ *G*), switching quantum yield (QY_sw_), and corresponding half‐lives (*t*_1/2_) of **CMR** motors at 20°C.

Motor (CMR)	Δ^‡^ *G′ (E_mst_* →*E_st_*) (kJ mol^−1^)^a^	*t*_1/2_′ (s)	QY_sw_ (*Z_st_* →*E_mst_*) (%)^b^	PSS ratio (*E_mst_*:*Z_st_*)^c^	Δ^‡^ *G″* (*Z*_mst_ → *Z*_st_) (kJ mol^−1^)^a^	*t*_1/2_″ (h)	QY_sw_ (*E*_st_→*Z*_mst_) (%)^b^	PSS ratio (*Z_mst_*:*E_st_*)^c^
**CMBZT**	79.9 ± 1.5	19.9	13.5 ± 0.6	98:2	101.9 ± 1.5	45.5	6.0 ± 0.2	> 9:1
**CMBZO**	80.0 ± 1.6	20.5	19.0 ± 2.1	96:4	100.7 ± 2.0	27.2	8.7 ± 0.2	>99:1
**CMBID**	80.6 ± 0.5	25.7	22.9 ± 2.3	97:3	101.5 ± 3.5	38.8	6.6 ± 0.6	98:2

^a^ Determined in THF at 20 °C.

^b^ This value was obtained using UV–visible spectroscopy with in situ irradiation.

^c^ This value was obtained using ^1^H‐NMR with in situ irradiation.

Eyring analysis of the THI processes monitored by UV–vis spectroscopy gave activation free energies of ∼ 80 and 102 kJ/mol at 293 K for the ***E_mst_***‐**CMR**→ ***E_st_***‐**CMR** and ***Z_mst_***‐**CMR**→ ***Z_st_***‐**CMR** conversions (Δ^⧧^ *G′* and Δ^⧧^ *G″*, respectively, Figures S5–S10). These values are comparable to those of the substituted first‐generation molecular motors, indicating that incorporation of the benzazole‐functionalized coumarin unit only slightly affects the THI barriers [16].

Furthermore, the quantum yields of both photochemical steps were determined using potassium ferrioxalate actinometry to evaluate the efficiency of the photoisomerization processes (Figures S43 and S44) [49]. All motors in both photochemical steps exhibited relatively high forward quantum yields, with ***Z_st_*‐CMBID** showing the highest value of 22% (*λ*_max_ = 455 nm for **CMBID** and **CMBZO**; *λ*_max_ = 470 nm for **CMBZT,** Figures S46 and S48). Together with the nearly quantitative photoisomerization observed across the series, irrespective of substitution pattern, these results demonstrate that incorporation of coumarin–benzazole motifs into the molecular motor scaffold constitutes a general strategy to red‐shift absorbance bands into the visible region in their neutral state (400–500 nm), while preserving high PSS ratios, efficient photochemical switching, and unaffected THI processes.

### 2.2 Protonation‐Gated Absorption Shifts

**FIGURE 3:**
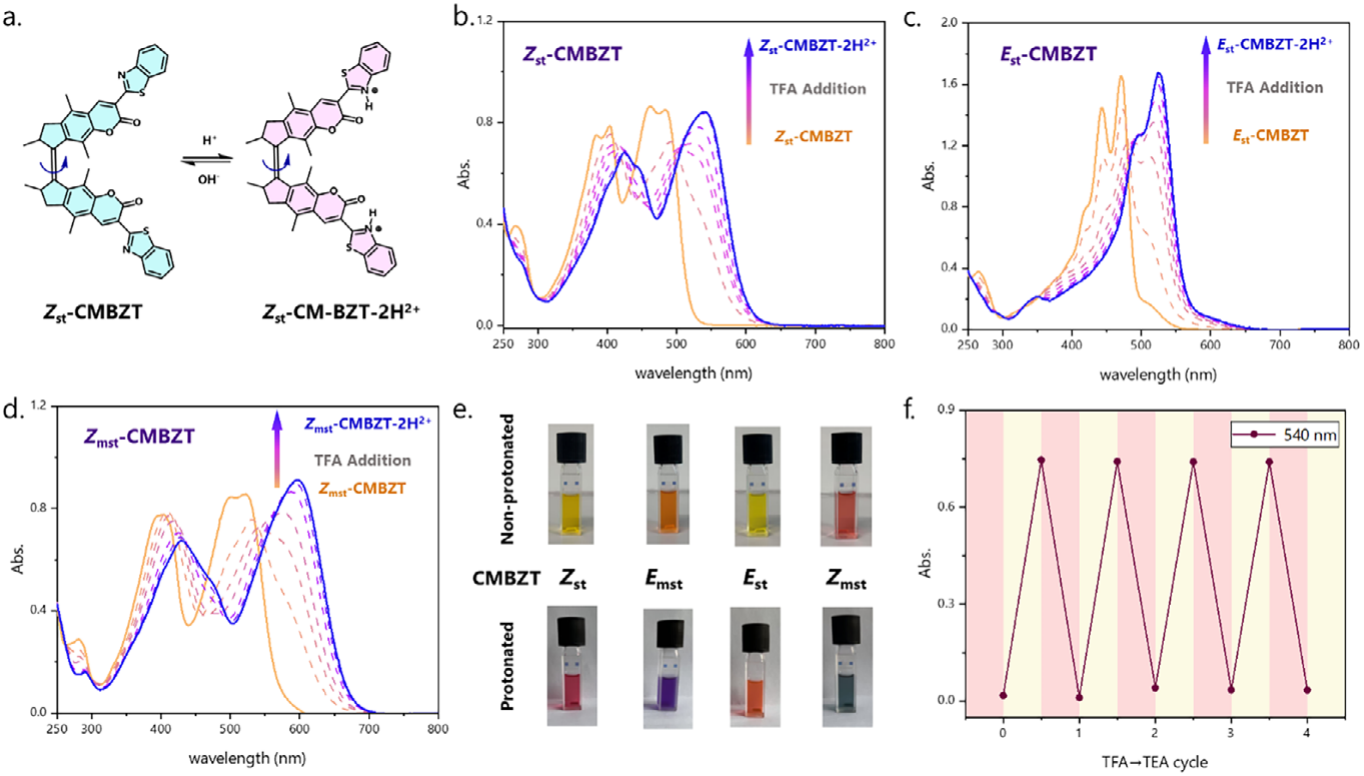
Protonation‐gated absorption shifts of the motors. (a) Reversible reaction of ***Z_st_*‐CMBZT** under TFA and TEA titrations. (b–d) Protonation process of ***Z_st_*‐CMBZT**, ***E_st_*‐CMBZT** and ***Z_mst_*‐CMBZT** with the addition of TFA (0 to 800 equiv, 0.1 M, diluted in DCM) in DCM solution recorded by UV–vis spectra. **e**. Photos of the color changes corresponding to the protonation process. **f**. Fatigue studies of ***Z_st_*‐CMBZT** (DCM, 1.5*10^−5^ M) before and after addition of TFA and TEA (1.5 equiv with respect to TFA).

Protonation of N‐containing heteroaromatic rings is a well‐established strategy for red‐shifting absorption, as protonation generally influences the electronic delocalization and stabilizes excited states [50, 51]. To assess the feasibility of this approach in our system and to identify the species formed upon acid treatment, we first examined **R1‐3**, a preliminary model bearing coumarin‐fused benzazole units (Figures S11–S13) [47]. ^1^H‐NMR analysis showed that alternating addition of trifluoroacetic acid (TFA) and triethylamine (TEA) at room temperature induced clean and reversible interconversion between the neutral benzazole and protonated benzazolium forms, with quantitative conversion in both directions. These results establish TFA/TEA cycling as a suitable platform for probing the formation and stability of the corresponding coumarin–benzazolium complexes. We then extended this analysis to the motor system ***Z_st_*‐CMR**. Acid–base titration monitored by ^1^H‐NMR revealed a quantitative and fully reversible protonation/deprotonation process (Figures 3a and S14–S16). The spectral changes were localized primarily in the aromatic region, consistent with protonation of the benzazole unit, whereas the aliphatic resonances associated with the motor core remained unchanged (Figures S14–S16). This behavior indicates that protonation occurs selectively at the heteroaromatic moiety without disrupting the structural framework of the motors.

To evaluate the extent of the protonation process and its impact on the photophysical properties of the motors, titration experiments were conducted with excess TFA (0.1 M, diluted in DCM), monitored by UV–vis spectroscopy. For ***Z_st_***‐**CMBZT**, stepwise addition of TFA (0 → 500 equiv.) facilitates the gradual formation of the protonated compound ***Z_st_***‐**CMBZT‐2H^2+^**, accompanied by a pronounced bathochromic shift of the absorption maximum, from $\lambda_{\mathrm{abs}.}^{\max}=470$ to 542 nm. This spectral change was consistent with the observed color transition from yellow to pink (Table S2 and Figures 3b,e). A similar trend was observed for the ***E_st_*** state and ***Z_mst_*** states, showing red‐shifted absorption peaks upon addition of TFA with Δ $\lambda_{\mathrm{abs}.}^{\max}$ of 55 nm for the ***E_st_***‐**CMBZT‐2H^2+^** state and 76 nm for the ***Z_mst_***‐**CMBZT‐2H^2+^** state. As a result, the corresponding protonated isomers show orange and green colors, respectively (Figure 3c,d). In addition, fatigue studies of all three ***Z_st_*‐CMR** isomers monitored by UV–vis spectroscopy revealed fully reversible protonation/deprotonation behavior with no detectable degradation (Figures 3f, S20, and S21).

**CMBZO** displayed a comparable response under TFA titration (0–800 equiv.), with progressive protonation inducing red‐shift of the absorption maxima for all three diastereomeric states. The corresponding Δ $\lambda_{\mathrm{abs}.}^{\max}$ values reached 88, 67, and 106 nm for the ***Z_st_***, ***E_st_***, and ***Z_mst_*** isomers, respectively (Figure S18). In contrast, protonation of the benzimidazole‐based **CMBID** was completed with substantially less TFA (0–100 equiv.), indicating a higher susceptibility toward protonation. However, **CMBID** exhibited markedly smaller bathochromic shifts than the corresponding **BZT** and **BZO** systems, with Δ $\lambda_{\mathrm{abs}.}^{\max}$ values of 48, 20, and 47 nm for the ***Z_st_***, ***E_st_***, and ***Z_mst_*** states, respectively (Figure S17). This difference between coumarin benzazole motors suggests that not only the ionization but also conformational changes might contribute to the UV–vis spectral shifts before and after the protonation process.

### 2.3 Conformational Analysis

To clarify the structural origin of the distinct protonation‐dependent spectral responses, density functional theory (DFT) calculations were performed. Ground‐state geometry optimizations were carried out for the possible isomers of the **CMR** derivatives at the B3LYP/6‐311++G(d,p) level with a SMD (THF/CH_2_Cl_2_) solvent model (Section S14). For each motor, three possible rotational conformers (conformer **a‐c**) were considered for both the neutral and protonated forms in both photoresponsive states (***Z_st_*** and ***E_st_***). As both states displayed closely analogous conformational trends, only the ***Z_st_*** state is discussed below. The corresponding proportion of different conformers, together with their relative free energies, are summarized in Tables S4–S6.

**FIGURE 4:**
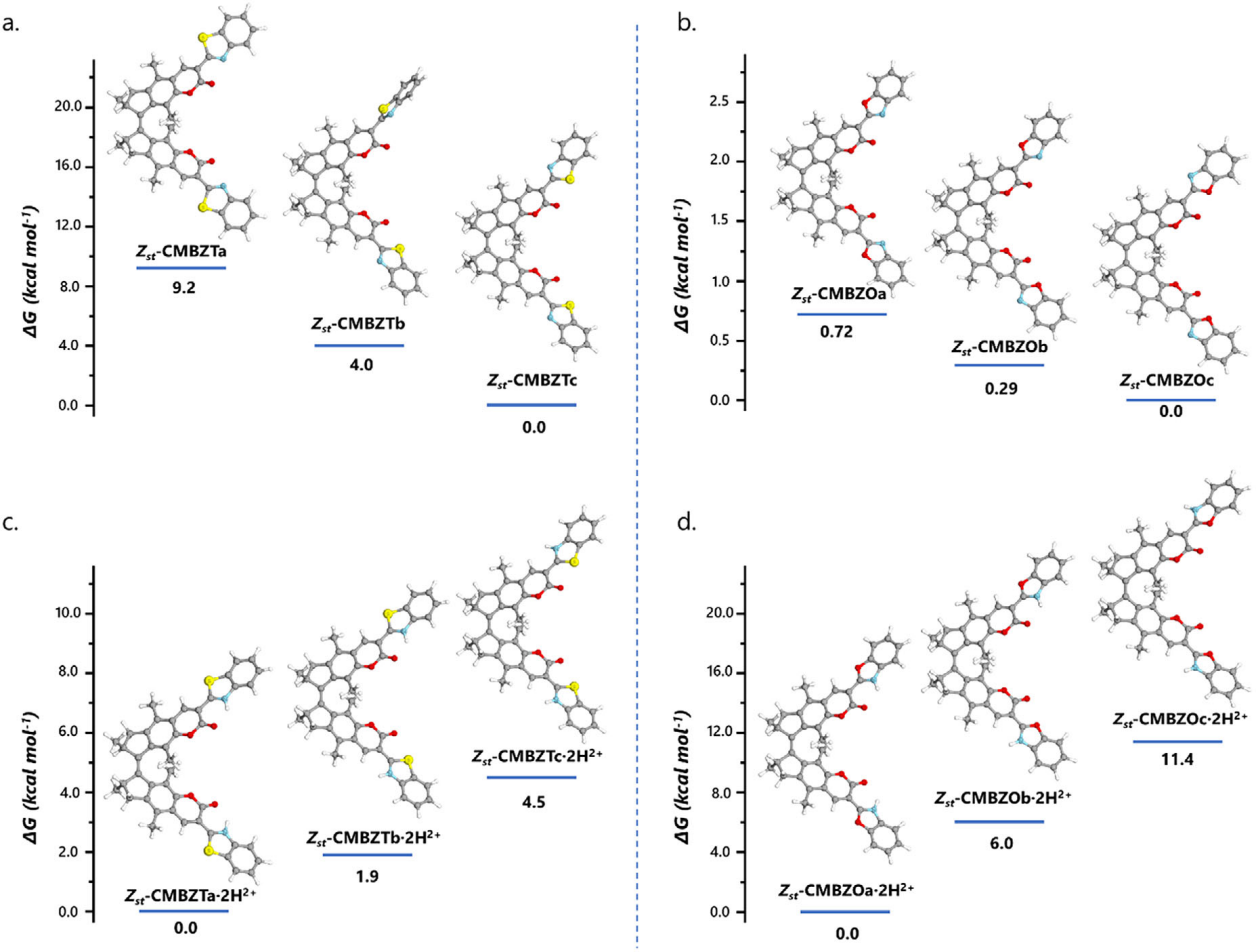
DFT optimized geometries of different conformers before and after protonation and the corresponding free‐energy diagrams of (a) ***Z_st_*‐CMBZT**, (b) ***Z_st_*‐CMBZO**, (c) ***Z_st_*‐CMBZT‐2H^2+^**, (d) ***Z_st_*‐CMBZO‐2H^2+^**, with the lowest free energy in each case set to 0. Atom colors: S, yellow; O, red; N, blue.

For ***Z_st_*‐CMBZT**, the neutral species exhibits a clear conformational preference. Among the three candidate conformers, ***Z_st_*‐BZTc** is calculated to be the most stable, showing 4.0 and 9.2 kcal mol^−1^ below ***Z_st_*‐BZTa** and ***Z_st_*‐BZTb**, respectively (Figure 4a). The reason for the stability could originate from the presence of an intramolecular chalcogen‐bonding interaction between the carboxyl group and the sulfur atom of the benzothiazole unit, with the S···O distance = 2.81 Å, which favors a more coplanar arrangement and thereby enhances conjugation between the coumarin scaffold and the benzothiazole fragment (Figures S60 and S62) [52, 53]. Upon protonation, however, the preferred geometry changes markedly (Figure 4c). In the protonated form, stabilization arises instead from an intramolecular hydrogen bond between the carboxyl group and the protonated benzothiazolium N–H unit, which favors a different rotational isomer in which the sulfur atom is oriented away from the carboxyl group (Figures S61 and S63) [45]. This energetic reordering indicates that protonation triggers a pronounced conformational reorganization in ***Z_st_*‐CMBZT‐2H^2+^**, which enhances delocalization and leads to a substantial reduction in the highest occupied molecular orbital (HOMO) and the lowest unoccupied molecular orbital (LUMO) gap (decreased from 2.80 to 2.45 eV), consistent with the large red‐shift observed experimentally (Figures S70 and S71).

A related but distinct behavior is observed for ***Z_st_*‐CMBZO**. In the neutral state, all three conformers (***Z_st_*‐BZOa**–**c**) adopt a relatively non‐planar geometry between the coumarin and benzoxazole fragments, with a dihedral angle ranging from approximately 4 to 22° (Figures S64 and S66). In contrast to ***Z_st_*‐CMBZT**, the relative free‐energy differences among these conformers are small, all within 1 kcal mol^−1^, indicating little intrinsic conformational preference (Figure 4b). After protonation, ***Z_st_*‐BZOa‐2H^2+^** becomes clearly favored, lying 6.0 and 11.4 kcal mol^−1^ below ***Z_st_*‐BZOb‐2H^2+^** and ***Z_st_*‐BZOc‐2H^2+^**, respectively (Figure 4d). This stabilization is associated with the formation of an intramolecular hydrogen bond in the protonated state, likewise producing a pronounced decrease in the HOMO–LUMO gap and a large bathochromic shift (Figures S72 and S73). Concomitantly, the dihedral angle decreases to 0°, consistent with increased planarity and enhanced π‐conjugation. By contrast, ***Z_st_*‐CMBID** is already conformationally preorganized in the neutral state by a stabilizing intramolecular hydrogen bond (Figures S68 and S69). Protonation therefore induces only limited additional structural reorganization and mainly retains the existing conformational preference. As a result, the additional reduction in the HOMO–LUMO gap is smaller, accounting for the reduced red‐shift of this system (Figures S74 and S75).

Thus, the calculated bathochromic shift can be understood as the combined result of structural and electronic effects. Protonation of benzazole‐substituted motors promotes a more planar and more strongly conjugated geometry, which improves orbital overlap across the π ‐system with reduced HOMO‐LUMO gap. In addition, the TD‐DFT simulated absorption spectra show that the low‐energy optically allowed transitions are shifted to longer wavelengths upon protonation. Collectively, these effects serve to reduce the excitation energy, thereby explaining the observed red shift in spectra.

### 2.4 Unidirectional Rotary Cycle of Motors in a Protonated State

**FIGURE 5:**
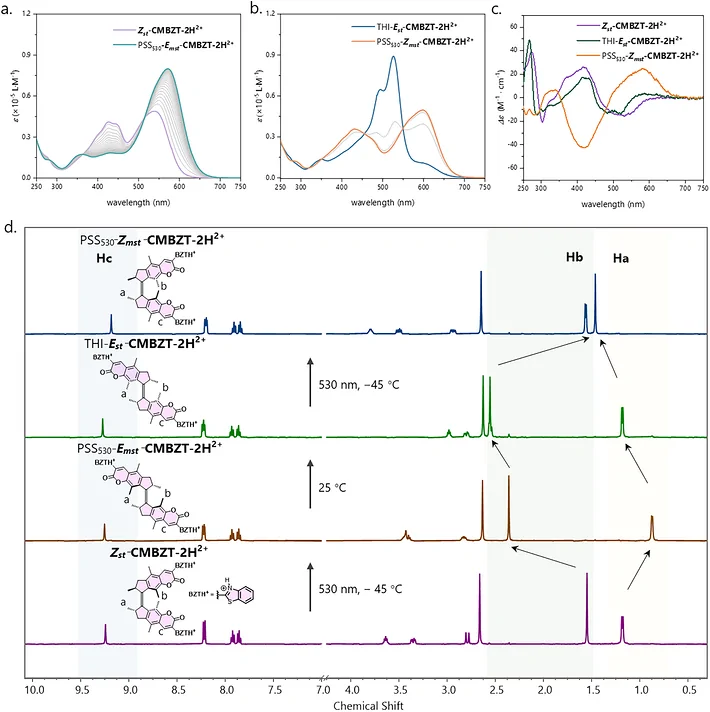
Rotary cycle of the **CMBZT‐2H^2+^** via UV–vis, ECD, and ^1^H‐NMR spectroscopies. (a) Changes in the UV–vis absorption spectra of ***Z_st_***‐**CMBZT‐2H^2+^** (purple line; DCM with 1000 equiv. TFA, −12 °C, 15 µM) upon irradiation at 530 nm to generate PSS_530_‐***E_mst_***‐**CMBZT‐2H^2+^** (green line), recorded every 5s (grey lines, indicating the intermediate spectra). (b) Changes in the UV–vis absorption spectra of ***E_st_***‐**CMBZT‐2H^2+^** (blue line; DCM with 1000 equiv. TFA, −12 °C, 15 µM) upon irradiation at 530 nm light to generate PSS_530_‐***Z_mst_***‐**CMBZT‐2H^2+^** (orange line), recorded every 5s (grey lines, indicating the intermediate spectra). (c) CD spectra starting from (*S*,*S*)‐(*M*,*M*)‐***Z_st_***‐**CMBZT‐2H^2+^** (purple line, DCM with 1000 equiv. TFA), followed by 530 nm light irradiation at –12°C of the sample and then heating to room temperature to obtain (*S*,*S*)‐(*M*,*M*)‐***E_st_***‐**CMBZT‐2H^2+^** (green line) and subsequent 530 nm irradiation to generate (*S*,*S*)‐(*P*,*P*)‐***Z_mst_***‐**CMBZT‐2H^2+^** (orange line). (d) Rotary cycle of motor **CMBZT‐2H^2+^** followed by ^1^H NMR spectroscopy (500 MHz, 2 mM in 0.5 mL CD_2_Cl_2_ with 11.5 µL TFA) starting from ***Z_st_***‐**CMBZT‐2H^2+^** (bottom red line) to PSS_530_‐***E_mst_***‐**CMBZT‐2H^2+^** (green line) under 530 nm light irradiation at –45 °C, followed by heating to room temperature for THI to generate THI‐***E_st_***‐**CMBZT‐2H^2+^** (cyan line); and subsequent irradiation under 530 nm light at –45°C to PSS_530_‐***Z_mst_***‐**CMBZT‐2H^2+^** (purple line). All ^1^H NMR measurements were performed at –45°C to ensure the chemical shifts were not influenced by temperature. Arrows were used to direct attention to the changes in the chemical shifts (*δ*, ppm) of the characteristic resonances.

Based on the titration experiments and corresponding DFT analysis, the pronounced bathochromic shift upon protonation suggested that these motors might be able to operate under substantially longer‐wavelength visible light irradiation. To assess this, UV–vis spectroscopy was first used to confirm that protonation preserves the overall rotary behavior of the motor while red shifting its photo‐responsive region. Irradiation of ***Z_st_*‐CMBZT‐2H^2+^** at −12°C with 530 nm light induced a clean spectral transformation from the initial protonated state to the PSS_530_‐***E_ms__t_*‐CMBZT‐2H^2+^**, which is evidenced from the decrease of the original absorption band at 400–500 nm with the appearance of a new absorption maximum up to 570 nm, with the presence of a clear isosbestic point at 505 nm (Figure 5a). Subsequently, the sample was kept in the dark at −12°C, which promoted the THI process with the formation of the corresponding THI‐***E_s__t_*‐CMBZT‐2H^2+^** diastereomer (Figure S22b). Further irradiation led to the second photochemical isomerization, generating PSS_530_‐***Z_mst_*‐CMBZT‐2H^2+^** and red‐shifting the absorption spectra further (Figure 5b). Finally, the initial protonated absorption pattern was fully recovered back to THI‐***Z_s__t_*‐CMBZT‐2H^2+^**, by reheating the generated solution, which indicates completion of the rotatory cycle (Figure S22). Notably, the multistate chiral information is retained upon protonation, with spectral signals closely resembling those of the non‐protonated state, while exhibiting a red shift of approximately 50–80 nm depending on the protonation state (Figure 5c). To further examine the robustness of the rotary operation after acid/base cycling, UV–vis switching experiments showed that the salt‐containing ***Z_st_***‐**CMBZT** sample still underwent efficient photoisomerization and subsequent thermal recovery process, indicating that accumulated triethylammonium trifluoroacetate does not significantly interfere with motor operation under the tested conditions (Figures S56–S59).

The impact of the pronounced bathochromic shift induced by protonation on motor function was next evaluated by ^1^H‐NMR spectroscopy under in situ irradiation conditions. Upon irradiation with 530 nm light at −45°C, ***Z_st_*‐CMBZT‐2H^2+^** underwent nearly quantitative photoconversion to the corresponding metastable state, as evidenced by the characteristic changes in the allylic and benzylic methyl resonances (Figure 5d). Subsequent thermal helix inversion and the second photochemical step proceeded cleanly with the formation of PSS_530_‐***Z_mst_*‐CMBZT‐2H^2+^** in the protonated states, ultimately regenerating THI‐***Z_st_*‐CMBZT‐2H^2+^** after warming the sample to room temperature for 30 days, consistent with completion of the unidirectional cycle (Figures S37 and S38).

A similar trend was observed for the protonated **CMBZO‐2H^2+^** and **CMBID‐2H^2+^** derivatives, which also retained the unidirectional rotary sequence of their neutral counterparts under red‐shifted excitation conditions (Figures S23, S24, S54, and S55). Importantly, the red‐shift is not gained at the expense of photochemical pathway selectivity or photo‐efficiency (Table 2). Integration of the characteristic ^1^H‐NMR signals revealed PSS ratios of >95:5 for all the photochemical processes in the protonated states of **CMR‐2H^2+^**, comparable to those of the neutral motor (Figures S39–S42). Additionally, these motors demonstrated again high forward switching efficiency after protonation, ranging from 2.8% to 5.3% for ***Z_st_* → *E_mst_*** upon 565 nm irradiation and 6.5% to 8.2% for ***E_st_* → *Z_mst_*** upon 530 nm irradiation (Figures S49–S51 and Table S3). Furthermore, Eyring analysis revealed that protonation had no significant influence on the THI process. The corresponding barriers for both THI steps were only slightly increased relative to those of the unprotonated species, with Δ^⧧^ *G′* values about 81 kJ mol^−1^ for the ***E_mst_***‐**CMR‐2H^2+^** → ***E_st_***‐**CMR‐2H^2+^** and Δ^⧧^ *G″* about 103 kJ mol^−1^ for ***Z_mst_***‐**CMR‐2H^2+^** → ***Z_st_***‐**CMR‐2H^2+^** steps at 293 K (Figures S25–S30). The slower THI of the protonated motor could be attributed to the modified conformational and electronic landscape after protonation, where intramolecular hydrogen bond, increased planarity, and solvation effects may stabilize the ***Z*_mst_** state and increase the structural reorganization required for helix inversion. These results show that protonation does not disrupt the rotary cycle, but instead enables state‐dependent operation at substantially longer visible‐light wavelengths than in the neutral system.

**TABLE 2:** Gibbs free energy of activation (Δ^‡^ *G*), switching quantum yield (QY_sw_), and corresponding half‐lives (*t*_1/2_) of **CMR‐2H^2+^** motors at 20°C.

Motor (CMR‐2H^2+^)	Δ^‡^ *G′ (E_mst_* →*E_st_*) (kJ mol^−1^)^a^	*t*_1/2_′ (s)	QY_sw_ (*Z_st_* →*E_mst_*) (%)^b^	PSS ratio (*E_mst_*: *Z_st_*)^c^	Δ^‡^ *G″* (*Z*_mst_ → *Z*_st_) (kJ mol^−1^)^a^	*t*_1/2_″ (h)	QY_sw_ (*E*_st_→*Z*_mst_) (%)^b^	PSS ratio (*Z_mst_*: *E_st_*)^c^
**CMBZT‐2H^2+^**	80.6 ± 1.3	26.0	2.8 ± 0.5	98:2	103.1 ± 1.8	73.3	8.2 ± 0.2	98:2
**CMBZO‐2H^2+^**	82.1 ± 1.8	48.3	5.3 ± 0.4	97:3	103.5 ± 0.2	87.4	6.5 ± 0.3	98:2
**CMBID‐2H^2+^**	80.9 ± 1.6	29.1	5.0 ± 0.1	97:3	103.4 ± 2.6	83.2	13.8 ± 0.6	98:2

^a^ Determined at 20 °C.

^b^ This value was obtained using UV–visible spectroscopy with in situ irradiation.

^c^ This value was obtained using ^1^H‐NMR with in situ irradiation.

**FIGURE 6:**
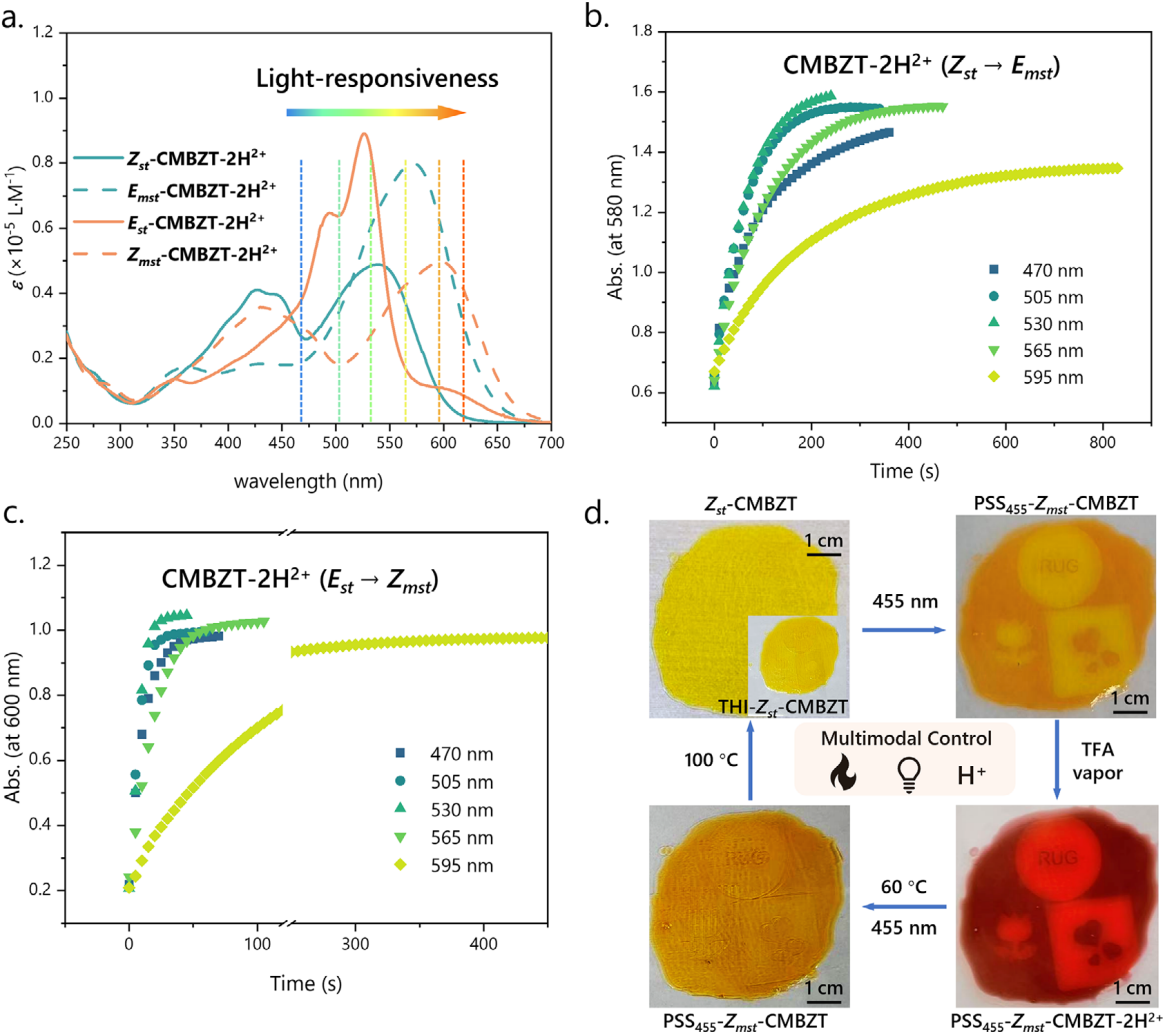
(a) UV–vis absorption spectra of **CMBZT‐2H^2+^** of four rotatory states in DCM (***Z_st_***‐**CMBZT‐2H^2+^**: solid cyan line; ***E_mst_***‐**CMBZT‐2H^2+^**: dash cyan line; ***E_st_***‐**CMBZT‐2H^2+^**: solid orange line; ***Z_mst_***‐**CMBZT‐2H^2+^**: dash orange line). Visible light‐responsiveness is revealed by using different irradiation wavelengths (from blue to red, indicated by a straight line). Evolution of the absorption changes during the irradiation of (b). ***Z_st_*‐CMBZT‐2H^2+^** and (c). ***E_st_*‐CMBZT‐2H^2+^** (2*10^−5^ M, −15°C in DCM) with 470, 505, 530, 565, and 595 nm light. (d) Multimodal regulation of patterned color switching in a polymer matrix with ***Z_st_***‐**CMBZT** through light, protonation, and heat.

To rigorously establish the motor's responsiveness across the entire visible region, irradiation experiments of **CMBZT‐2H^2+^** and **CMBZO‐2H^2+^** were performed using blue, green, yellow, and red light (from 470 to 617 nm, Figure 6a), while monitoring the spectral evolution by UV–vis spectroscopy. In each case, the UV–vis spectra showed clear photoisomerization from stable to metastable forms. Kinetic analysis of the absorption maxima was employed to track the progress of the photochemical process. Although the time required to reach the PSS state varied with irradiation wavelength, all wavelengths effectively promoted the photoconversion (Figures 6b,c, S52, and S53). Notably, the absorption reached almost the same point upon longer exposure for the ***E_st_* → *Z_mst_*** transformation under all wavelengths examined, indicating near quantitative photoconversions were achieved (Figure S52b). Collectively, the results establish protonation as an effective chemical gating mechanism that extends orthogonal motor operation to the blue‐to‐red visible wavelength range while preserving the efficiency and directionality of the light‐responsive system.

To further evaluate the applicability of this dual‐responsive system in a solid‐state medium, a predefined pattern was fabricated by incorporating the ***Z_st_*‐CMBZT** molecules into a styrene–ethylene–butylene–styrene triblock copolymer matrix [41, 54]. The patterned film exhibited distinct multicolor transitions under the combined regulation of light, heat, and proton stimuli (Figure 6d). Initially, the film showed a transparent yellow appearance, corresponding to the doped ***Z_st_*‐CMBZT** compound (Figure 6d, top left). Upon irradiation with 455 nm light through different masks, the exposed regions gradually turned orange, whereas the masked regions remained yellow, consistent with the photoisomerization process from ***Z_st_*‐CMBZT** to PSS_455_‐***Z_mst_*‐CMBZT** upon light irradiation (Figure 6d, top right). Subsequent exposure to TFA vapor protonated both states, inducing distinct color changes from yellow to red (***Z_st_*‐CMBZT** → ***Z_mst_*‐CMBZT‐2H^2^** ^+^) and from orange to dark purple (PSS_455_‐***Z_mst_*‐CMBZT** → PSS_455_‐***Z_mst_*‐CMBZT‐2H^2^** ^+^), respectively (Figure 6d, bottom right). Importantly, the patterned features remained clearly discernible throughout the whole process, demonstrating effective orthogonal modulation of the photoisomerization and protonation of the motors in the polymers. The acid‐generated patterns could be erased under simultaneous 455 nm irradiation and heating at 60°C, yielding a homogenized orange state as excess TFA evaporated and promoted the rotation toward PSS_455_‐***Z_mst_*‐CMBZT** in the whole area (Figure 6d, bottom left). Further heating at 100°C for 2 h restored the original yellow state, indicating the THI process from PSS_455_‐***Z_mst_*‐CMBZT** to THI‐***Z_st_*‐CMBZT** (Figure 6d, top left). These results demonstrate that the capability of the present system to achieve reversible multistate color switching in a patterned polymer film through light, thermal, and protonic inputs, suggesting its potential in advanced information encryption and smart display materials.

## 3 Conclusion

In this study, we have developed a protonation‐gated molecular motor platform in which reversible chemical input orthogonally controls light responsiveness by modulating conjugation topology. In the neutral state, these coumarin‐fused motors undergo unidirectional rotation with near‐quantitative photoconversion under blue‐to‐green irradiation. Protonation induces structural reorganization, enhances π‐delocalization, and produces an approximately 100 nm bathochromic shift, thereby extending motor operation across the broad visible spectrum. Notably, this reprogramming of the excitation window occurs without compromising photochemical selectivity, switching efficiency, or directionality of rotation. Moreover, the solid‐state patterning experiment demonstrates that this dual‐responsive molecular motor system enables reversible and spatially resolved multistate color switching in a polymer matrix under orthogonal light, heat, and proton control. These results highlight reversible protonation‐gated photochromism as an effective design strategy for adaptive molecular motor systems with broad visible‐light responsiveness, with clear implications for future materials and biological applications.

## Author Contributions

**Jinghao Wang**: conceptualization, methodology, investigation, formal analysis, writing – original draft, writing – review and editing. **Alexander Ryabchun**: methodology, investigation, formal analysis, writing – review and editing. **Ben L. Feringa**: conceptualization, formal analysis, supervision, funding acquisition, writing – review and editing.

## Conflicts of Interest

The authors declare no conflicts of interest.

## Supporting information




**Supporting File 1**: The authors have cited additional references within the Supporting Information [49, 55, 56, 57, 58, 59, 60, 61, 62, 63, 64, 65, 66, 67, 68, 69, 70, 71].

**Supporting File 2**: anie73618‐sup‐0002‐data.zip.

## Data Availability

The data that supports the findings of this study are available in the Supporting Information of this article.
