# Supplementary material for: Protonation‐Gated Orthogonal Control of Highly Efficient Molecular Motors With Broad Visible‐Light Responsiveness
Source: Angew Chem Int Ed Engl. 2026 Jul 17;65(38):e4214875. doi: 10.1002/anie.4214875 (PMC13573078; doi:10.1002/anie.4214875)
Supplement: Supplementary file 1 [file ANIE-65-e4214875-s002.pdf]

Protonation-Gated Orthogonal Control of Highly Efficient Molecular Motors with Broad Visible-Light Responsiveness

Jinghao Wang, Alexander Ryabchun, Ben L. Feringa*

Stratingh Institute for Chemistry, University of Groningen, Nijenborgh 3, 9747 AG Groningen, The Netherlands.

*To whom correspondence should be addressed.

E-mail: b.l.feringa@rug.nl (B.L.F.)

1. Materials 3
2. General methods 3
3. Synthetic route 4
4. Motor rotatory cycles recorded by UV/vis absorption spectra before protonation 6
5. Kinetic studies of thermal helix inversion steps of motors before protonation 9
6. Protonation of CMR using TFA 12
7. Motor rotatory cycles recorded by UV/vis absorption spectroscopy after protonation 20
8. Kinetic studies of thermal helix inversion steps of motors after protonation 23
9. Unidirectional rotation and PSS ratio determination of motors 26
10. Rotation quantum yield determination 39
11. Visible light-responsiveness of CMR-2H^{2+} revealed by UV/vis absorption spectra 47
12. Electronic circular dichroism spectra 48
13. UV/vis spectra of rotatory cycles of the CMR after TFA/TEA cycles 49
14. Computational Analysis 51
15. NMR spectra 64
16. HRMS data of the synthesized compounds 67
17. References 69

1. Materials

All reagents from commercial sources such as Aldrich, TCI, BLD, combi-Blocks were used without further purification. Anhydrous DCM, DCE and THF was obtained from a solvent purification system (MBRAUN SPS systems, MBSPS-800). Solvents used in the irradiation experiments were degassed by purging with argon for 30 min. Styrene–ethylene–butylene–styrene triblock copolymer (SEBS, Mediprene-500120M, HEXPOL TPE AB, Sweden) was employed as the polymer matrix to embed the molecular motors.[1] For column chromatography, silica gel (Silicycles Siliaflash P60, 40–60 μm, 230–400 mesh) was used in all cases. Separation was carried out on silica gel 60 (silicon dioxide, SiO_2; Merck, Germany) and kieselguhr F254 (Celite; Merck, Germany) for thin-layer chromatography (TLC), and visualization was achieved either by potassium permanganate ($KMnO_4$) staining or under UV light. All reactions were performed under a N_2 atmosphere.

2. General methods

NMR spectra were recorded at 25 °C on Varian AMX400 (^{1}H: 400 MHz, ^{13}C: 101 MHz), Varian Unity Plus (^{1}H: 500 MHz, ^{13}C: 125 MHz) spectrometers. Chemical shifts (δ) are expressed relative to the resonances of the residual non-deuterated solvent for ^{1}H NMR [$CDCl_3$: ^{1}H(δ) = 7.26 ppm, CD_2Cl_2: ^{1}H(δ) = 5.32 ppm] and ^{13}C NMR [$CDCl_3$: ^{13}C(δ) = 77.2 ppm, CD_2Cl_2: ^{13}C (δ) = 53.84 ppm]. Absolute values of the coupling constants are given in Hertz (Hz), regardless of their sign. Multiplicities are abbreviated as singlet (s), doublet (d), doublet of doublets (dd), triplet (t), triplet of doublets (td), quartet (q), multiplet (m), and broad (br). High-resolution mass spectrometry (HRMS) was performed on an LTQ Orbitrap XL spectrometer with ESI ionization. All reactions were performed under an N_2 atmosphere. UV-vis spectra were recorded on a Hewlett-Packard HP 8543 spectrometer in a quartz cuvette with 1 cm path length. ECD spectra were recorded on Jasco J-815 Circular Dichroism Spectrometer. Irradiation experiments were performed using LED lamps (Thorlab) at 455/470/505/530/565/595/617 nm. The set up and methods for recording the rotation quantum yield were described in the specific section. Polymer films were prepared by drop-casting the mixture of polymers (~ 250 mg/ml in Toluene) with $\boldsymbol{Z_{st}}$**-CMBZT** (0.5 mg/ml, 0.2 *wt*%) on the glass substrate. Patterns were created by irradiation on top of the polymer films using the corresponding LED with a collimator through different masks.

3. Synthetic route

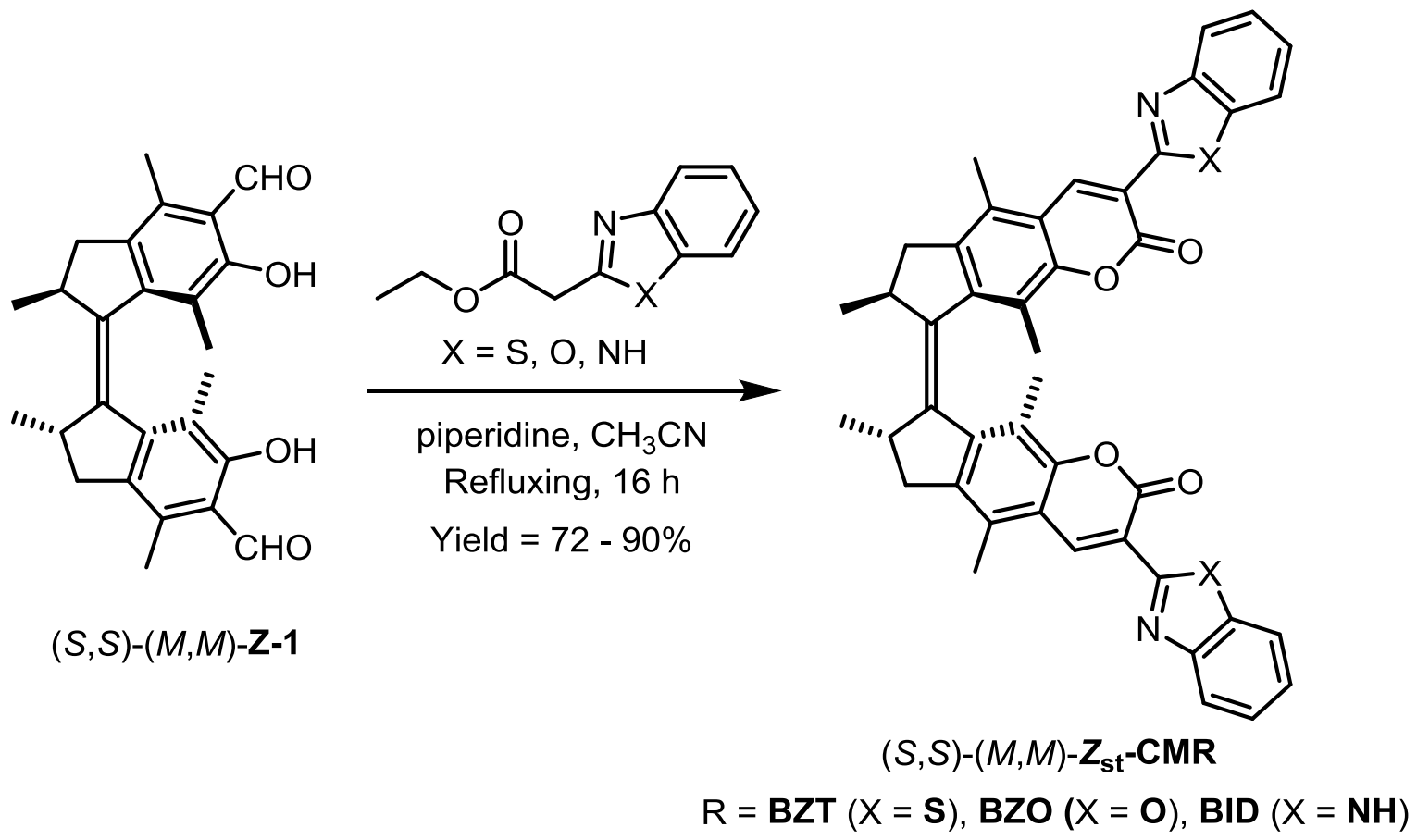

Figure S1. Overall synthetic scheme.

General Method: The reaction was set up in the dark. Under N_2, aldehyde motor (*S*,*S*)-(*M*,*M*)-**Z-1**[2] (40.5 mg, 100 µmol, 1.0 equiv.) and ethyl-2-(benzo[d]thiazol-2-yl)acetate / ethyl-(1H-benzimidazol-2-yl)acetate / ethyl-2-(benzo[d]oxazol-2-yl)acetate (250 µmol, 2.5 equiv.) were dissolved in dry acetonitrile (9.9 mL).[3] Piperidine (0.10 mL of a 0.1 mol/L stock solution in acetonitrile, 10.0 µmol, 10 mol%) was added and the viscous suspension was stirred and heated at reflux for 16 h. After completion of the reaction, the mixture was quenched with aq. saturated Na_2CO_3 (10 mL). The aqueous phase was extracted with EtOAc (3*25 mL) and the combined organic layers were washed with brine, dried over Na_2SO_4 and concentrated under vacuo. The crude product was purified by column chromatography (SiO_2, pentane:EtOAc = 10:1 to 4:1) to afford the target motors (*S*,*S*)-(*M*,*M*)-***Z**$_{st}$*-**CMR**.

CMBZT

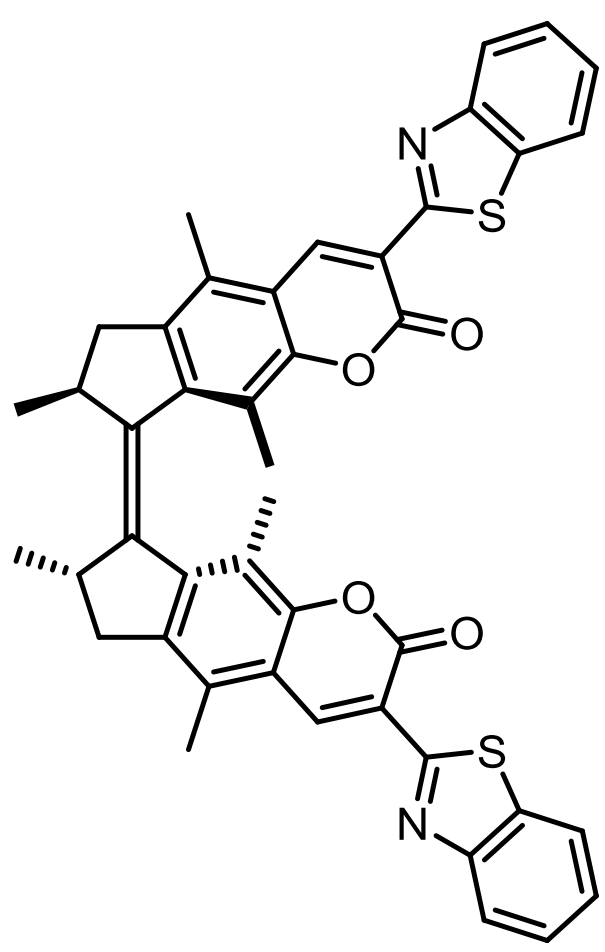

(*S*,*S*)-(*M*,*M*)-***Z**$_{st}$*-**CMBZT**

Starting from 40.5 mg of (*S*,*S*)-(*M*,*M*)-**Z-1** (0.10 mmol) and 55.3 mg of ethyl 2-(benzo[d]thiazol-2-yl)acetate (0.25 mmol), 64.4 mg of (*S*,*S*)-(*M*,*M*)-***Z**$_{st}$*-**CMBZT** was obtained as orange-red solid (90 %). ^{1}H NMR (400 MHz, $CDCl_3$) δ 9.30 (s, 2H), 8.11 (d, *J* = 8.2 Hz, 2H), 7.98 (d, *J* = 8.0 Hz, 2H), 7.53 (t, *J* = 7.7 Hz, 2H), 7.41 (t, *J* = 7.6 Hz, 2H), 3.52 (p, *J* = 6.8 Hz, 2H), 3.27 (dd, *J* = 15.0, 6.3 Hz, 2H), 2.69 – 2.65 (m, 8H), 1.63 (s, 6H), 1.17 (d, *J* = 6.7 Hz, 6H). ^{13}C NMR (101 MHz, $CDCl_3$) δ 160.74, 160.04, 152.61, 152.48, 145.95, 144.09, 140.62, 138.61, 136.95, 129.86, 126.29, 125.07, 122.73, 121.75, 121.10, 118.12, 117.35, 41.83, 38.60, 20.37, 14.77, 14.21. HRMS (ESI pos) calcd. for $[M+H]^+$: 719.2032, found: 719.2035.

CMBID

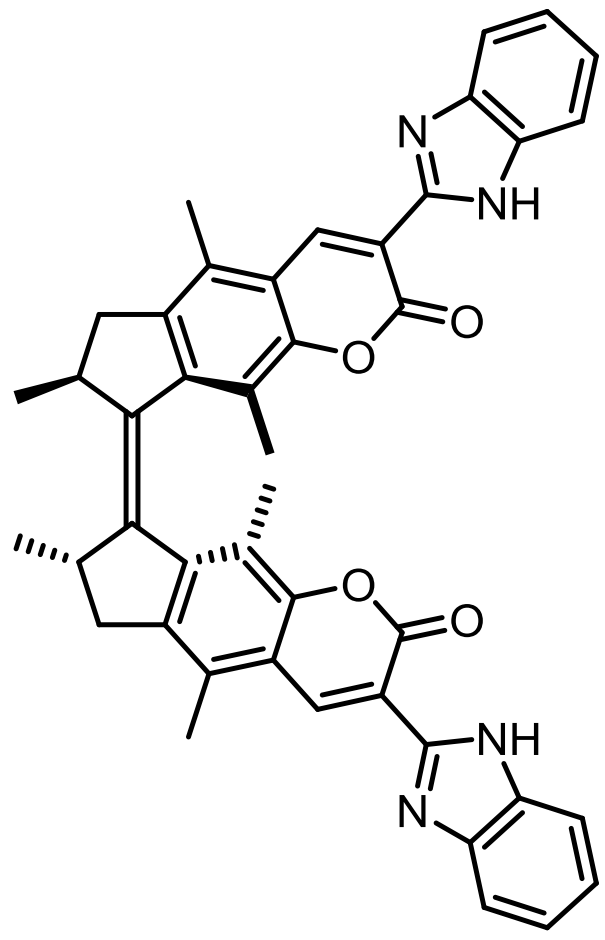

(*S*,*S*)-(*M*,*M*)-Z_{st}-**CMBID**

Starting from 40.5 mg of (*S*,*S*)-(*M*,*M*)-***Z*-1** (0.10 mmol) and 51.1 mg of ethyl (1H-benzimidazol-2-yl)acetate (0.25 mmol), 56.8 mg of (*S*,*S*)-(*M*,*M*)-$\boldsymbol{Z_{st}}$-**CMBID** was obtained as orange-red solid (83 %). ^{1}H NMR (400 MHz, $CDCl_3$) 11.43 (s, 2H), 9.31 (s, 2H), 7.91 – 7.76 (m, 2H), 7.61 – 7.51 (m, 2H), 7.33 (dd, *J* = 6.6, 2.9 Hz, 4H), 3.51 (q, *J* = 6.4 Hz, 2H), 3.27 (dd, *J* = 14.9, 6.2 Hz, 2H), 2.66 (m, 8H), 1.64 (s, 6H), 1.17 (d, *J* = 6.8 Hz, 6H). ^{13}C NMR (101 MHz, $CDCl_3$) δ 161.15, 152.26, 146.93, 145.78, 144.03, 143.44, 140.88, 139.25, 133.71, 129.71, 123.55, 122.96, 120.99, 119.19, 117.51, 113.83, 111.56, 41.81, 38.60, 20.36, 14.81, 14.24. HRMS (ESI pos) calcd. for $[M+H]^+$: 685.2809, found: 685.2810.

CMBZO

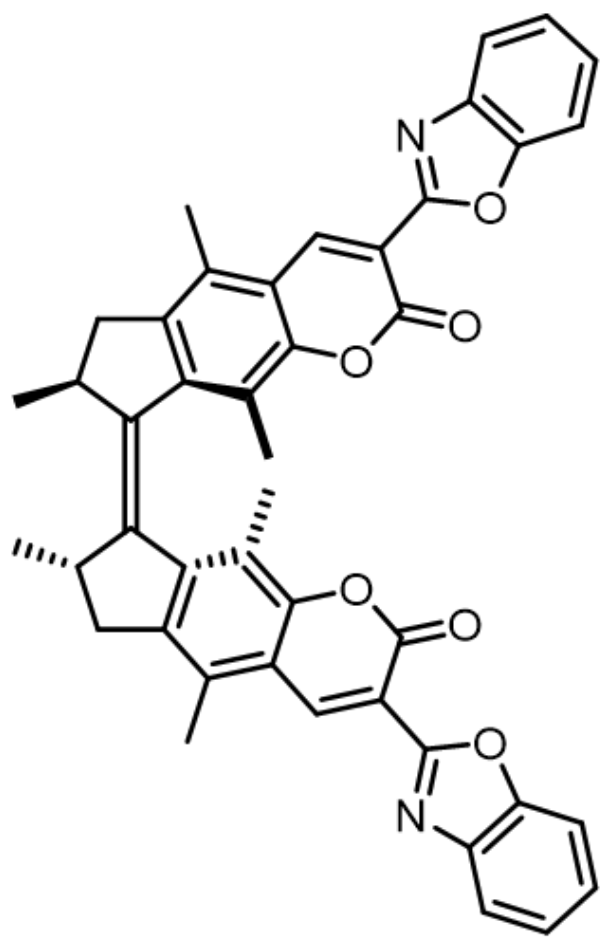

(*S*,*S*)-(*M*,*M*)-Z_{st}-**CMBZO**

Starting from 40.5 mg of (*S*,*S*)-(*M*,*M*)-***Z*-1** (0.10 mmol) and 51.3 mg of ethyl 2-(benzo[d]oxazol-2-yl)acetate (0.25 mmol), 49.4 mg of (*S*,*S*)-(*M*,*M*)-$\boldsymbol{Z_{st}}$-**CMBZO** was obtained as orange-red solid (72 %). ^{1}H NMR (400 MHz, $CDCl_3$) δ 9.00 (s, 2H), 7.90 – 7.84 (m, 2H), 7.70 – 7.63 (m, 2H), 7.45 – 7.34 (m, 4H), 3.52 (t, *J* = 6.6 Hz, 2H), 3.26 (dd, *J* = 15.1, 6.3 Hz, 2H), 2.66 (d, *J* = 14.9 Hz, 2H), 2.61 (s, 6H), 1.63 (s, 6H), 1.17 (d, *J* = 6.7 Hz, 6H). ^{13}C NMR (101 MHz, $CDCl_3$) δ 159.04, 156.84, 153.25, 150.68, 146.51, 144.23, 142.23, 141.87, 140.47, 129.51, 125.74, 124.81, 121.24, 120.56, 116.80, 112.83, 110.86, 41.88, 38.56, 20.33, 14.65, 14.21. HRMS (ESI pos) calcd. for $[M+H]^+$: 687.2490, found: 687.2492.

4. Motor rotatory cycles recorded by UV/vis absorption spectra before protonation

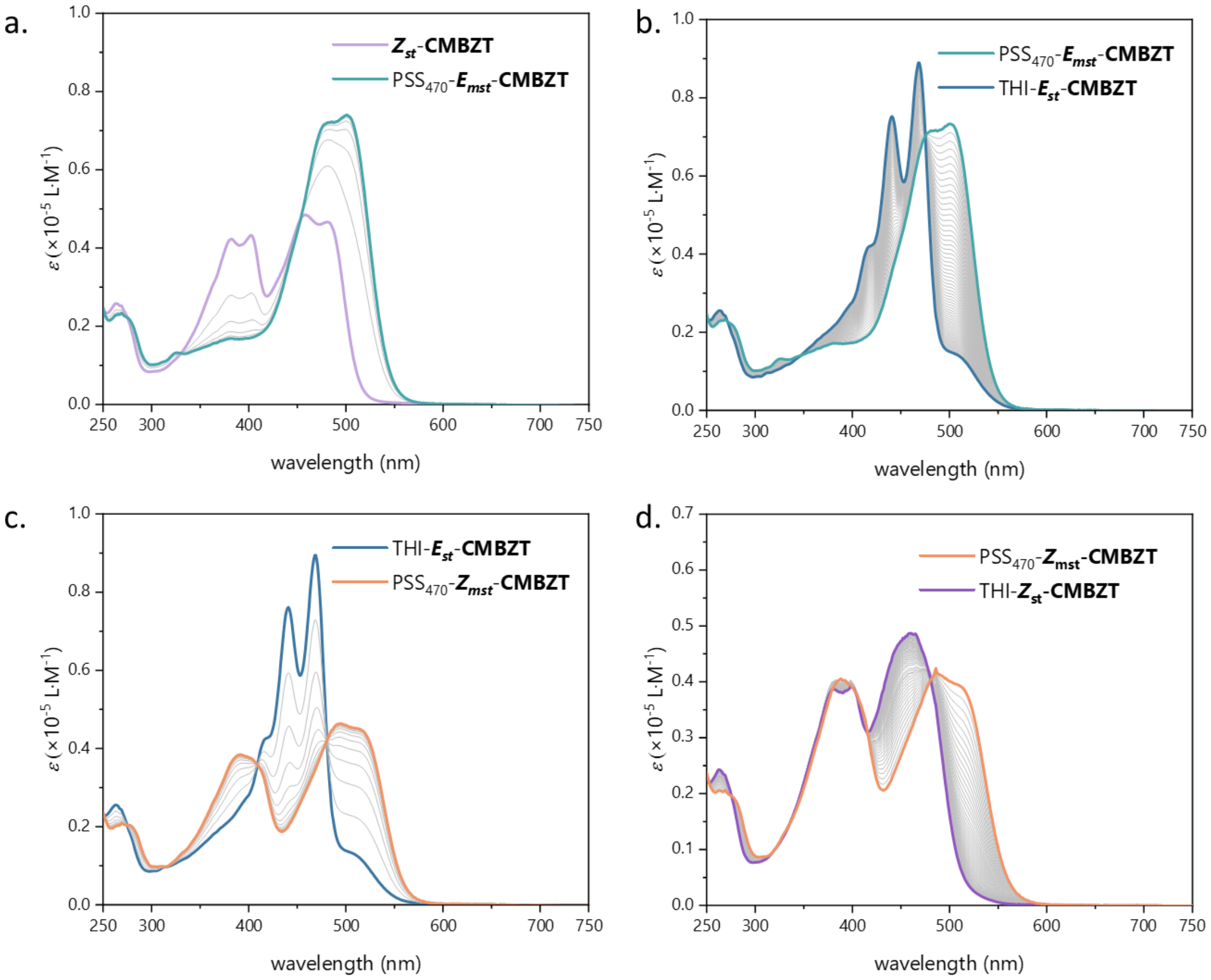

Figure S2. Unidirectional process of **CMBZT** ($1.5*10^{-5}$ M, in THF) recorded by UV/vis absorption spectroscopy. **a.** UV-vis spectra of ***Z**st*-**CMBZT** (light purple) upon 470 nm light irradiation to get PSS470-***E**mst*-**CMBZT** (light green line, − 12 ºC). **b.** maintaining in the dark for 2 h to reach THI-***E**st*-**CMBZT** (blue line, −12 ºC). **c.** subsequent irradiating with 470 nm light to get PSS470-***Z**mst*-**CMBZT** (orange line, −12 ºC). **d.** finally keeping in the dark for 3 h to recover THI-***Z**st*-**CMBZT** (purple line, 57 ºC**)**.

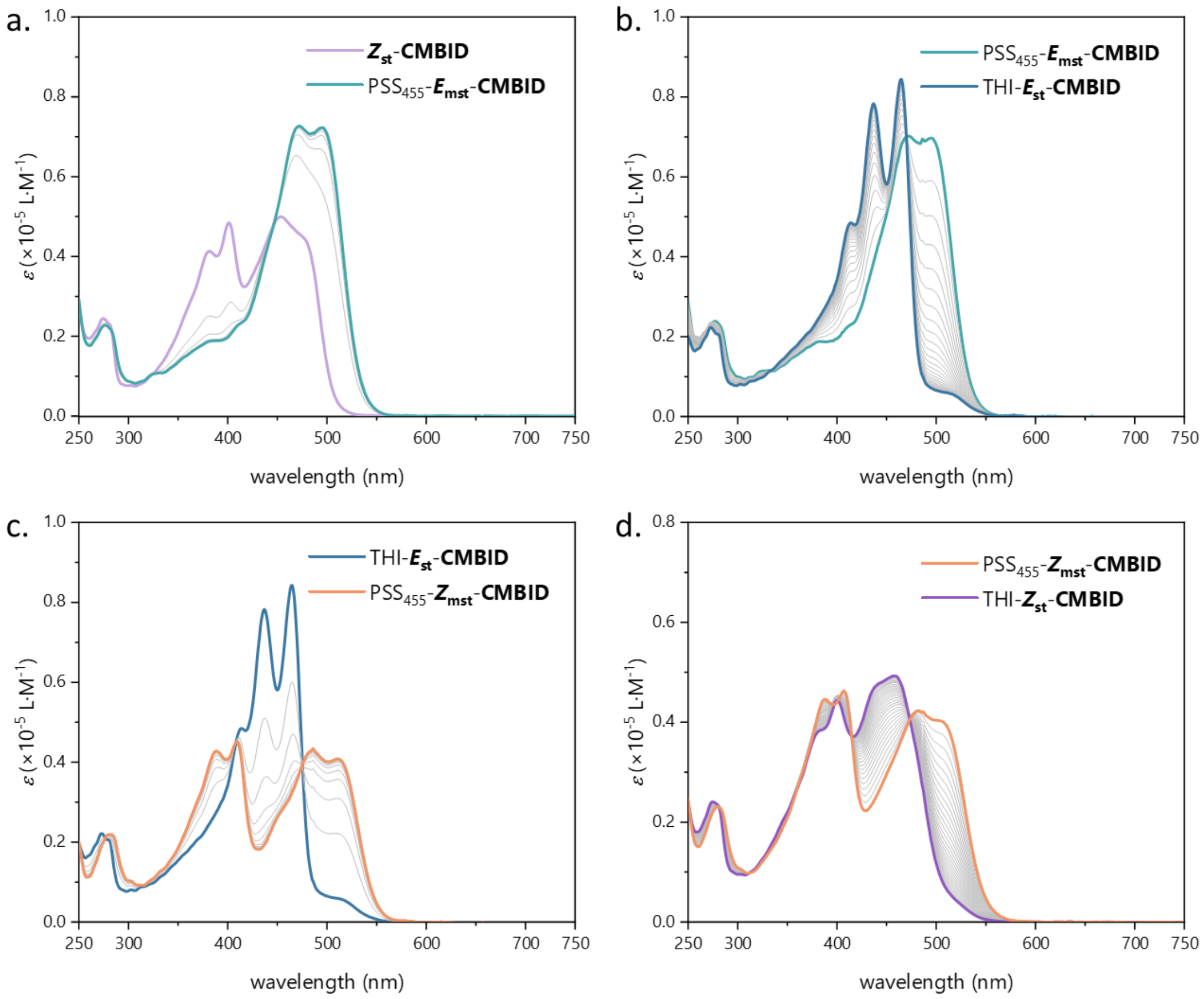

Figure S3. Unidirectional process of **CMBID** ($1.5*10^{-5}$ M, in THF) recorded by UV/vis absorption spectroscopy. **a.** UV-vis spectra of ***Z**$_{st}$***-CMBID** (light purple) upon 455 nm light irradiation to get PSS_{455}-***E**$_{mst}$***-CMBID** (light green line, − 12 ºC). **b.** maintaining in the dark for 2 h to reach THI-***E**$_{st}$***-CMBID** (blue line, − 12 ºC). **c.** subsequent irradiating with 455 nm light to get PSS_{455}-***Z**$_{mst}$***-CMBID** (orange line, − 12 ºC). **d.** finally keeping in the dark for 3 h to recover THI-***Z**$_{st}$***-CMBID (**purple line, 57 ºC**)**.

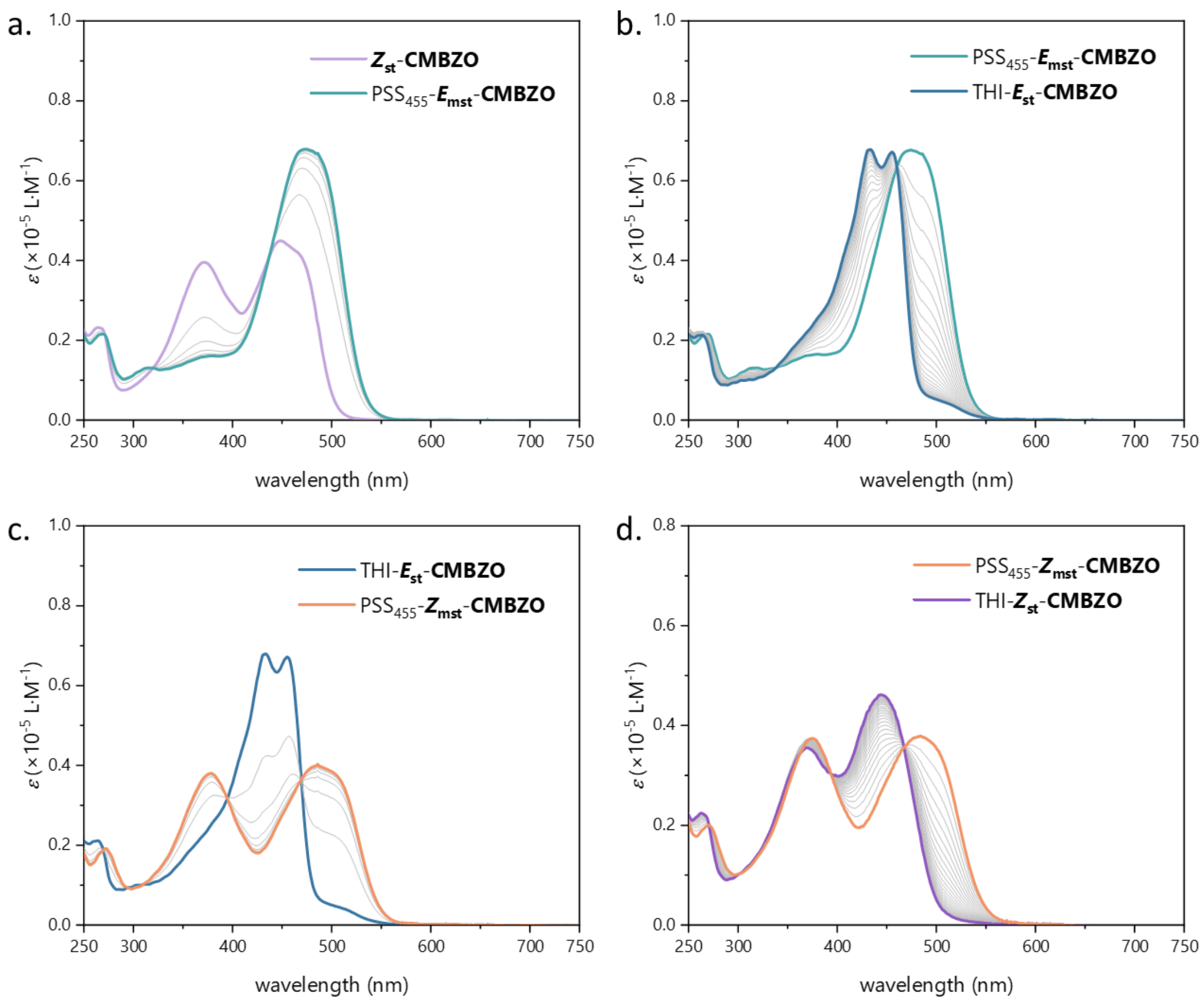

Figure S4. Unidirectional process of **CMBZO** ($1.5*10^{-5}$ M, in THF) recorded by UV/vis absorption spectroscopy. **a.** UV-vis spectra of ***Z****st***-CMBZO** (light purple) upon 455 nm light irradiation to get PSS$_{455}$-***E****mst***-CMBZO** (light green line, − 12 ºC). **b.** maintaining in the dark for 2 h to reach THI-***E****st***-CMBZO** (blue line, − 12 ºC). **c.** subsequent irradiating with 455 nm light to get PSS$_{455}$-***Z****mst***-CMBZO** (orange line, − 12 ºC). **d.** finally keeping in the dark for 3 h to recover THI-***Z****st***-CMBZO (**purple line, 57 ºC**)**.

5. Kinetic studies of thermal helix inversion steps of motors before protonation

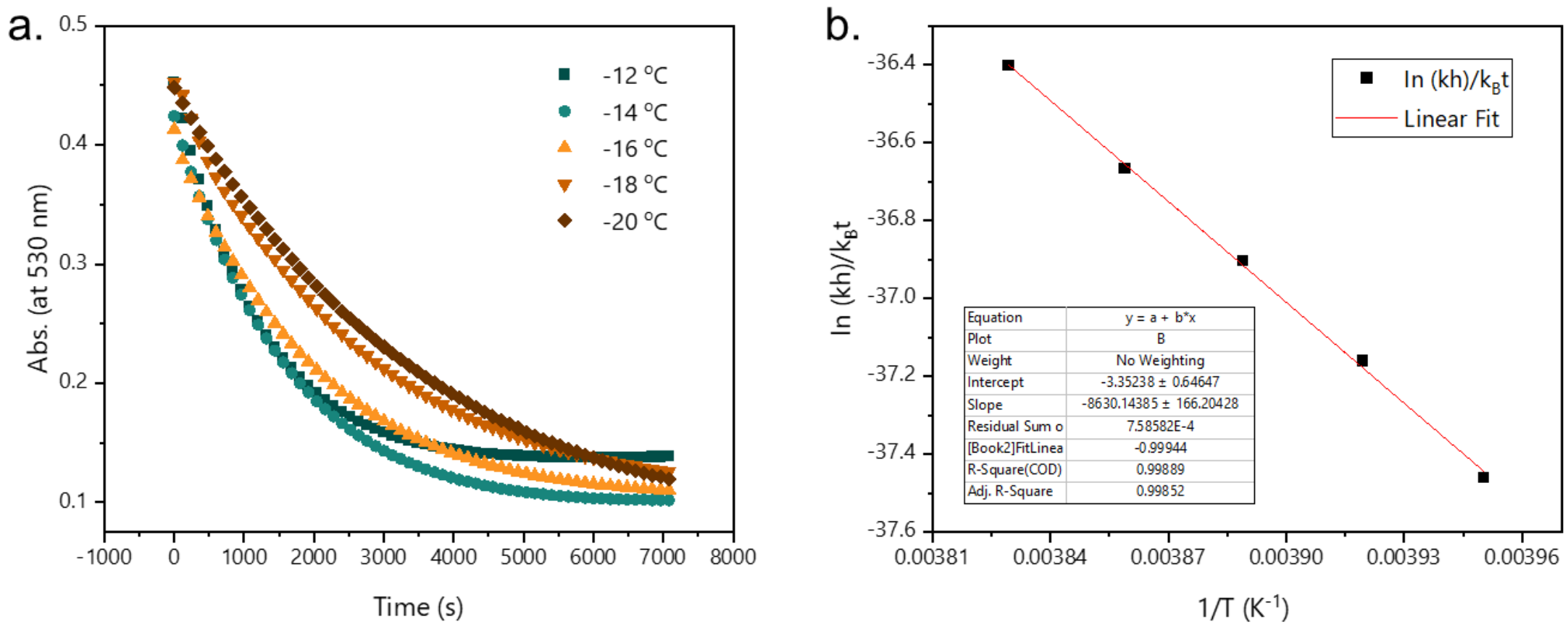

Figure S5. Kinetic studies of thermal helix inversion steps of PSS$_{470}$-***E$_{mst}$*-CMBZT** based on UV/vis absorption spectra. **a.** Changes of the absorption values at 530 nm of (THF, 1.5*10^{-5} M) at different temperature (-20 ℃ to -12 ℃), which shows the thermal helix inversion (THI) process with the formation of predominantly THI-***E$_{st}$*-CMBZT** motor. **b.** Eyring plot of THI step from ***E$_{mst}$*-CMBZT** to ***E$_{st}$*-CMBZT** with insertion of the linear fitting parameters of the Eyring analysis.[4]

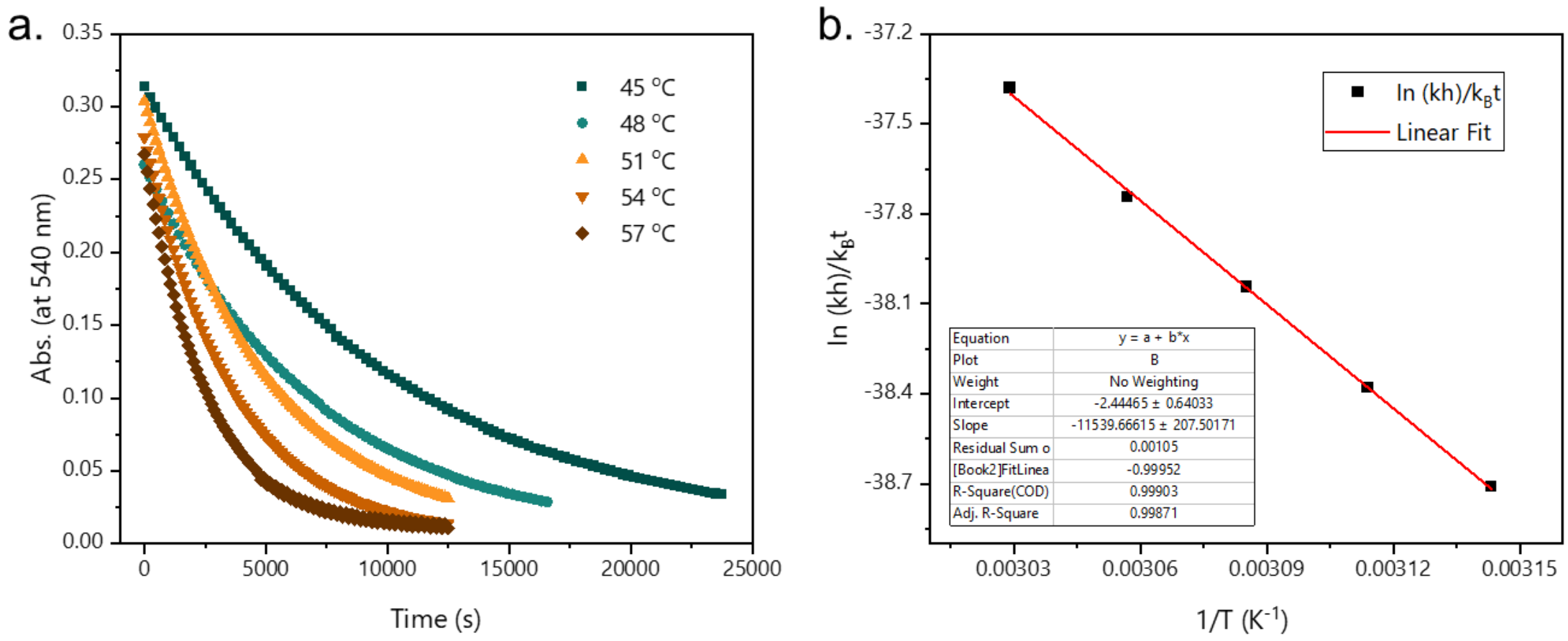

Figure S6. Kinetic studies of thermal helix inversion steps of PSS$_{470}$-***Z$_{mst}$*-CMBZT** based on UV/vis absorption spectra. **a.** Changes of the absorption values at 540 nm of (THF, 1.5*10^{-5} M) at different temperature (45 ℃ to 57 ℃), which shows the thermal helix inversion (THI) process with the formation of predominantly THI-***Z$_{st}$*-CMBZT** motor. **b.** Eyring plot of THI step from ***Z$_{mst}$*-CMBZT** to ***Z$_{st}$*-CMBZT** with insertion of the linear fitting parameters of the Eyring analysis.

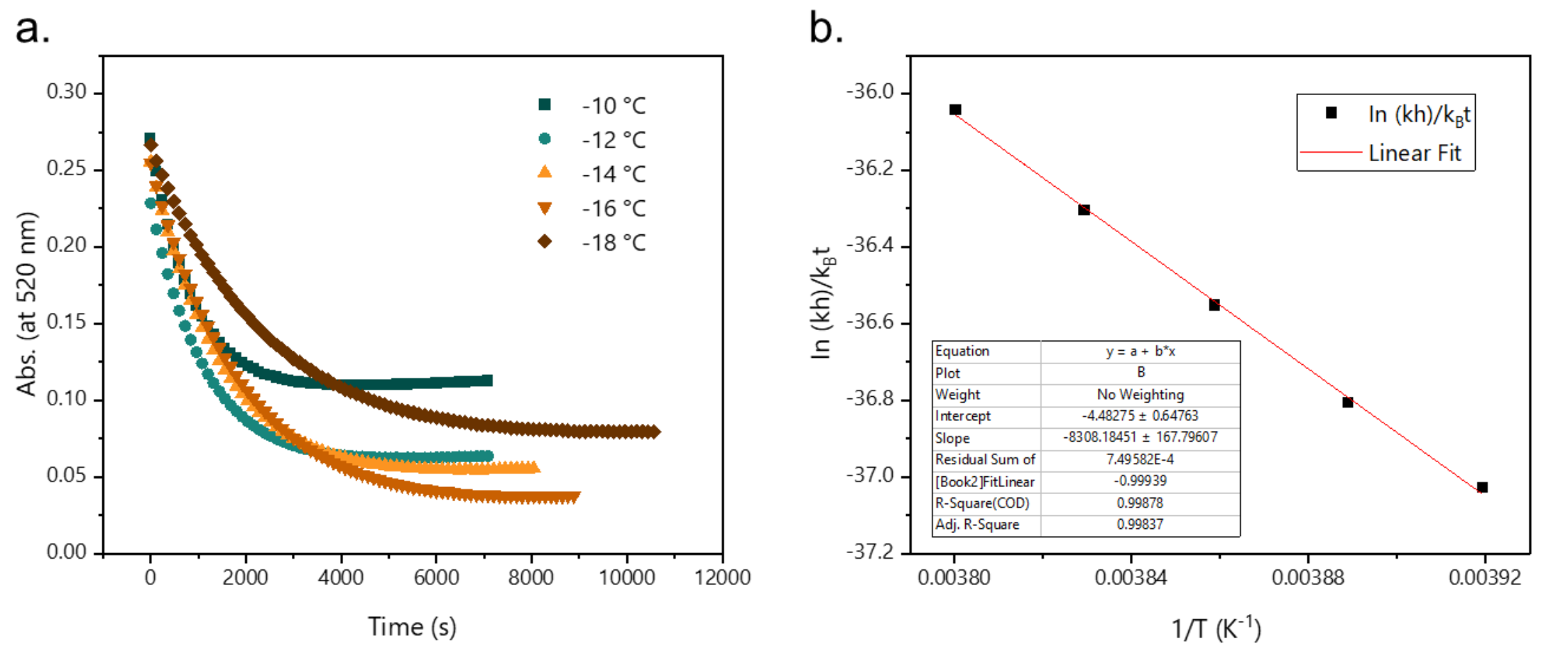

Figure S7. Kinetic studies of thermal helix inversion steps of PSS455-$\boldsymbol{E_{mst}}$-**CMBZO** based on UV/vis absorption spectra. **a.** Changes of the absorption values at 520 nm of (THF, 1.5*10^{-5} M) at different temperature (-18 ºC to -10 ºC), which shows the thermal helix inversion (THI) process with the formation of predominantly THI-$\boldsymbol{E_{st}}$-**CMBZO** motor. **b.** Eyring plot of THI step from $\boldsymbol{E_{mst}}$-**CMBZO** to $\boldsymbol{E_{st}}$-**CMBZO** with insertion of the linear fitting parameters of the Eyring analysis.

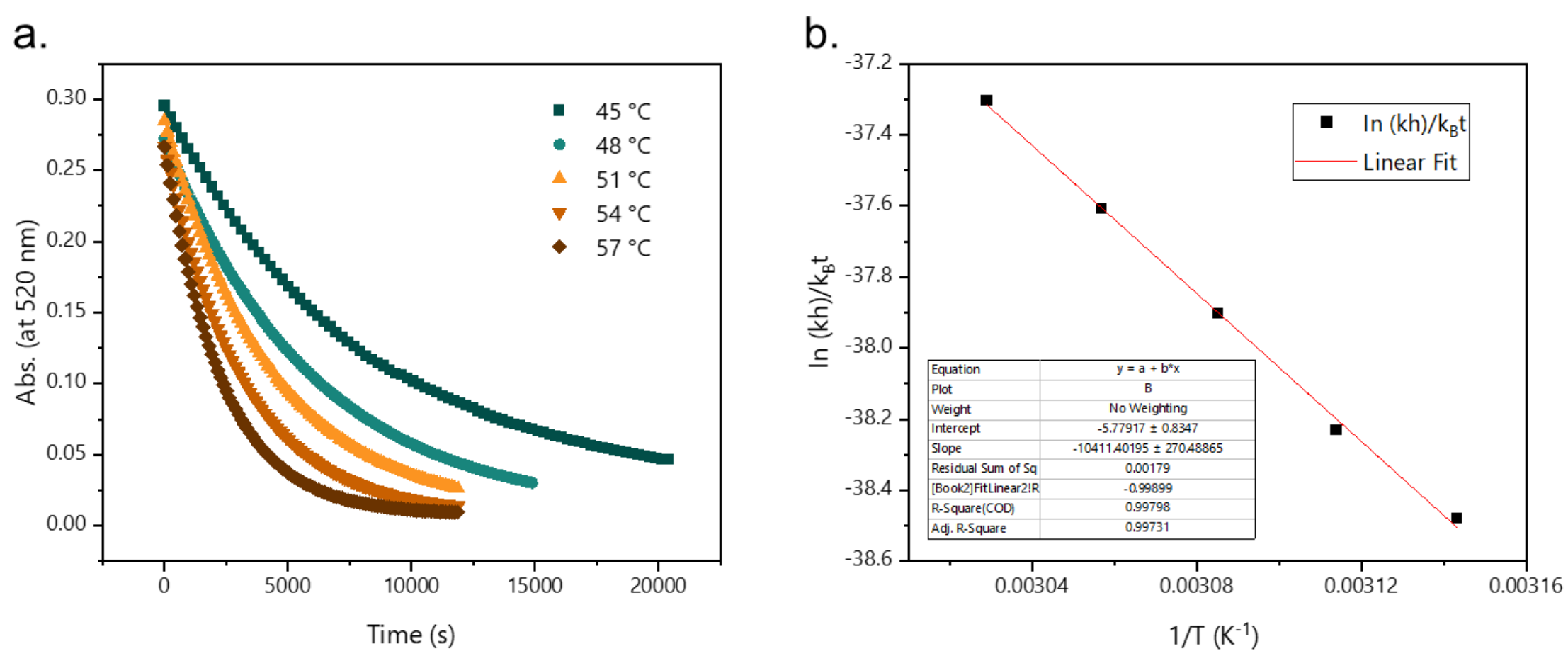

Figure S8. Kinetic studies of thermal helix inversion steps of PSS455-$\boldsymbol{Z_{mst}}$-**CMBZO** based on UV/vis absorption spectra. **a.** Changes of the absorption values at 520 nm of (THF, 1.5*10^{-5} M) at different temperature (45ºC to 57 ºC), which shows the thermal helix inversion (THI) process with the formation of predominantly THI-$\boldsymbol{Z_{st}}$-**CMBZO** motor. **b.** Eyring plot of THI step from $\boldsymbol{Z_{mst}}$-**CMBZO** to $\boldsymbol{Z_{st}}$-**CMBZO** with insertion of the linear fitting parameters of the Eyring analysis.

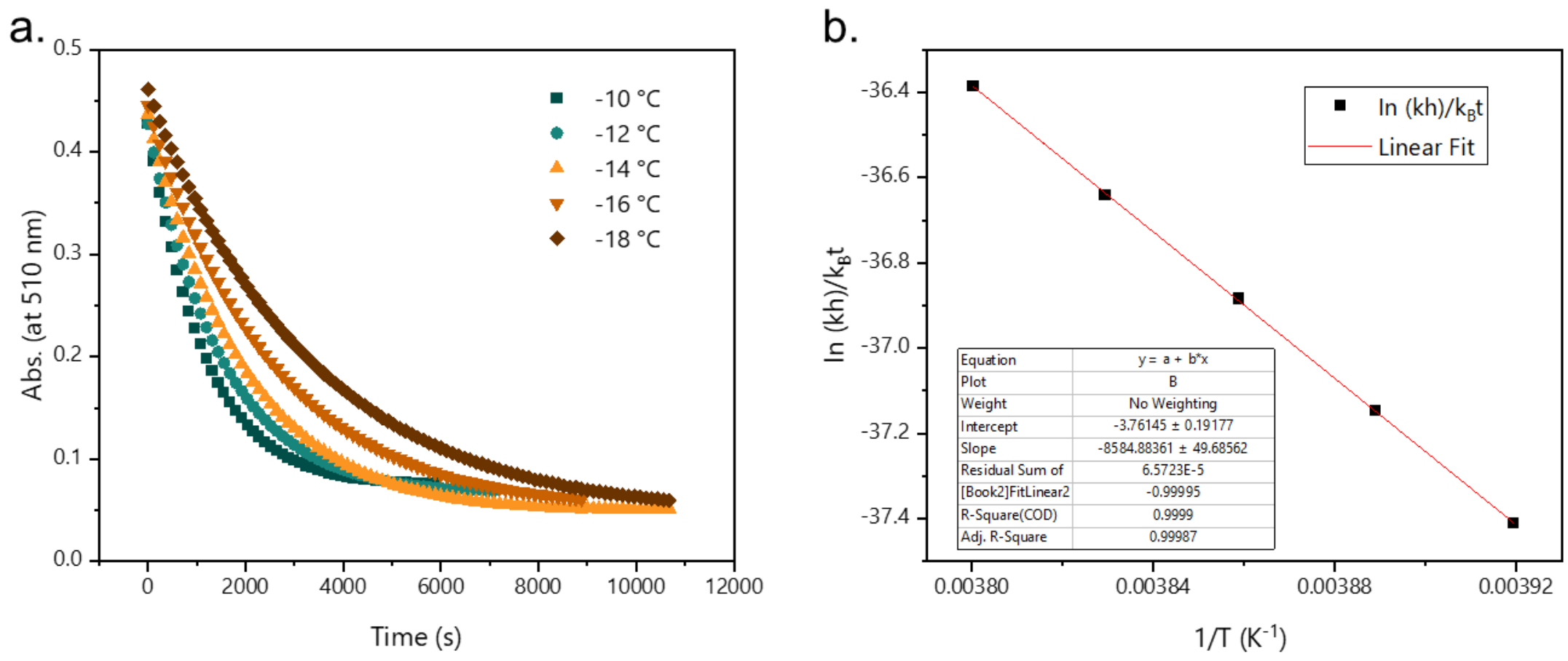

Figure S9. Kinetic studies of thermal helix inversion steps of PSS455-***E***$_{mst}$**-CMBID** based on UV/vis absorption spectra. **a.** Changes of the absorption values at 510 nm of (THF, 1.0*10^{-5} M) at different temperature (-18 ºC to -10 ºC), which shows the thermal helix inversion (THI) process with the formation of predominantly THI-***E***$_{st}$**-CMBID** motor. **b.** Eyring plot of THI step from ***E***$_{mst}$**-CMBID** to ***E***$_{st}$**-CMBID** with insertion of the linear fitting parameters of the Eyring analysis.

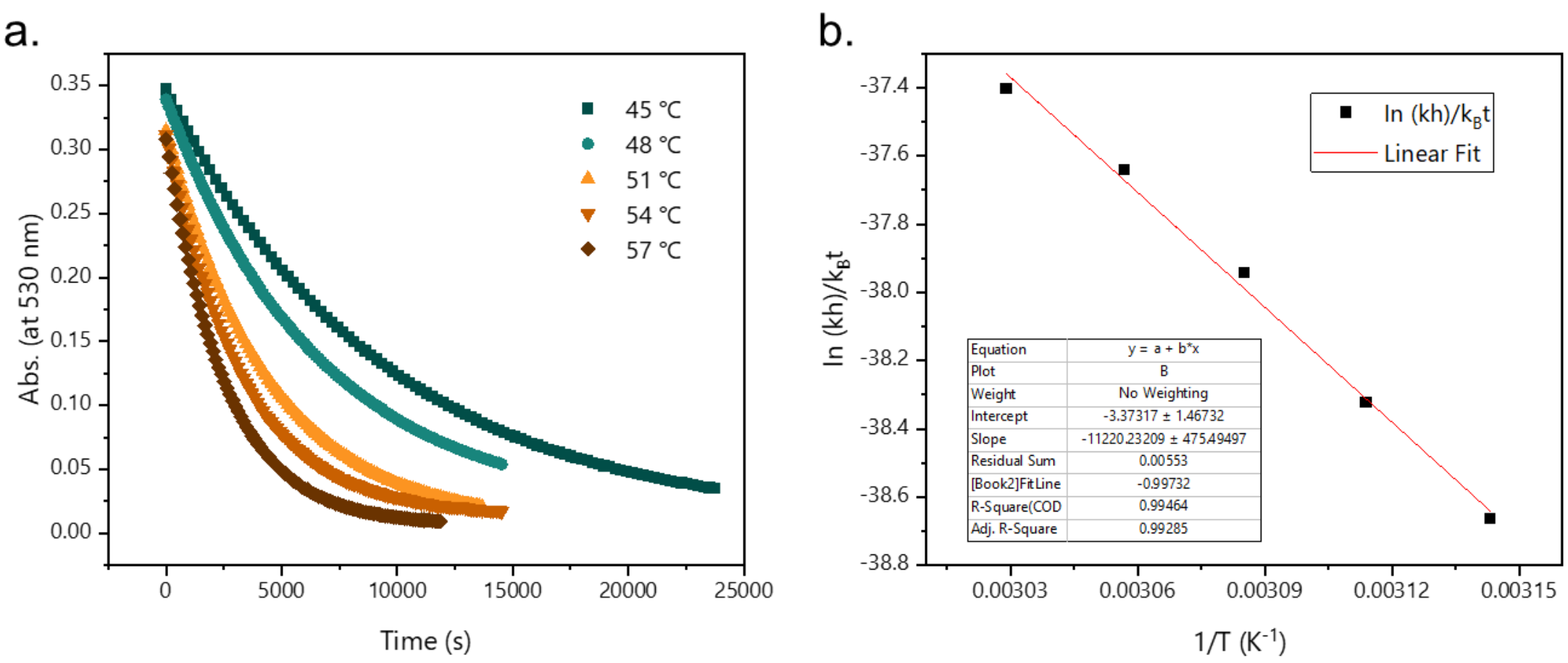

Figure S10. Kinetic studies of thermal helix inversion steps of PSS455-***Z***$_{mst}$**-CMBID** based on UV/vis absorption spectra. **a.** Changes of the absorption values at 530 nm of (THF, 1.0*10^{-5} M) at different temperature (45 ºC to 57 ºC), which shows the thermal helix inversion (THI) process with the formation of predominantly THI-***Z***$_{st}$**-CMBID** motor. **b.** Eyring plot of THI step from ***Z***$_{mst}$**-CMBID** to ***Z***$_{st}$**-CMBID** with insertion of the linear fitting parameters of the Eyring analysis.

6. Protonation of CMR using TFA

The coumarin benzazoles analogues displayed different pKa according to the literature.[5] To achieve the efficient protonation/deprotonation process, trifluoroacetic acid (TFA) and triethyl amine (TEA) were applied. To assess the extent of protonation under equilibrium conditions, dichloromethane was selected as the solvent due to its compatibility with both the **CMR** motors and the effective ionization of TFA.[6-8] In order to demonstrate the feasibility and identify the products of the TFA adducts, we selected the compound **R1-3** together with coumarin benzazoles analogues (***Z$_{st}$*-CMR**) and confirmed the efficient protonation and reversible mechanism of the acid-base reaction using ^{1}H-NMR spectroscopy.

For the titration experiment conducted in the UV/Vis spectroscopy, the protonation behavior of the motors in the ***Z$_{st}$***, ***E$_{st}$*** and ***Z$_{mst}$*** isomeric states were explored, as these isomers exhibit sufficient stability at room temperature. For clarity, the **CMR** motors after protonation were named as **CMR-2H^{2+}** .

General note on protonation/deprotonation conditions.

The amount of TFA required to reach protonation equilibrium depends on the molecular structure, substituent, solvent, concentration, and measurement conditions. For the NMR experiments, where relatively concentrated samples were used, 50–250 equiv. of TFA were generally sufficient to achieve complete protonation. In contrast, larger amounts of TFA, typically 100–1000 equiv., were required for the more diluted UV/vis and ECD measurements to ensure that the protonation equilibrium was fully reached under the spectroscopic conditions. Deprotonation was achieved by subsequent addition of TEA, with the amount adjusted according to the TFA used in each experiment.

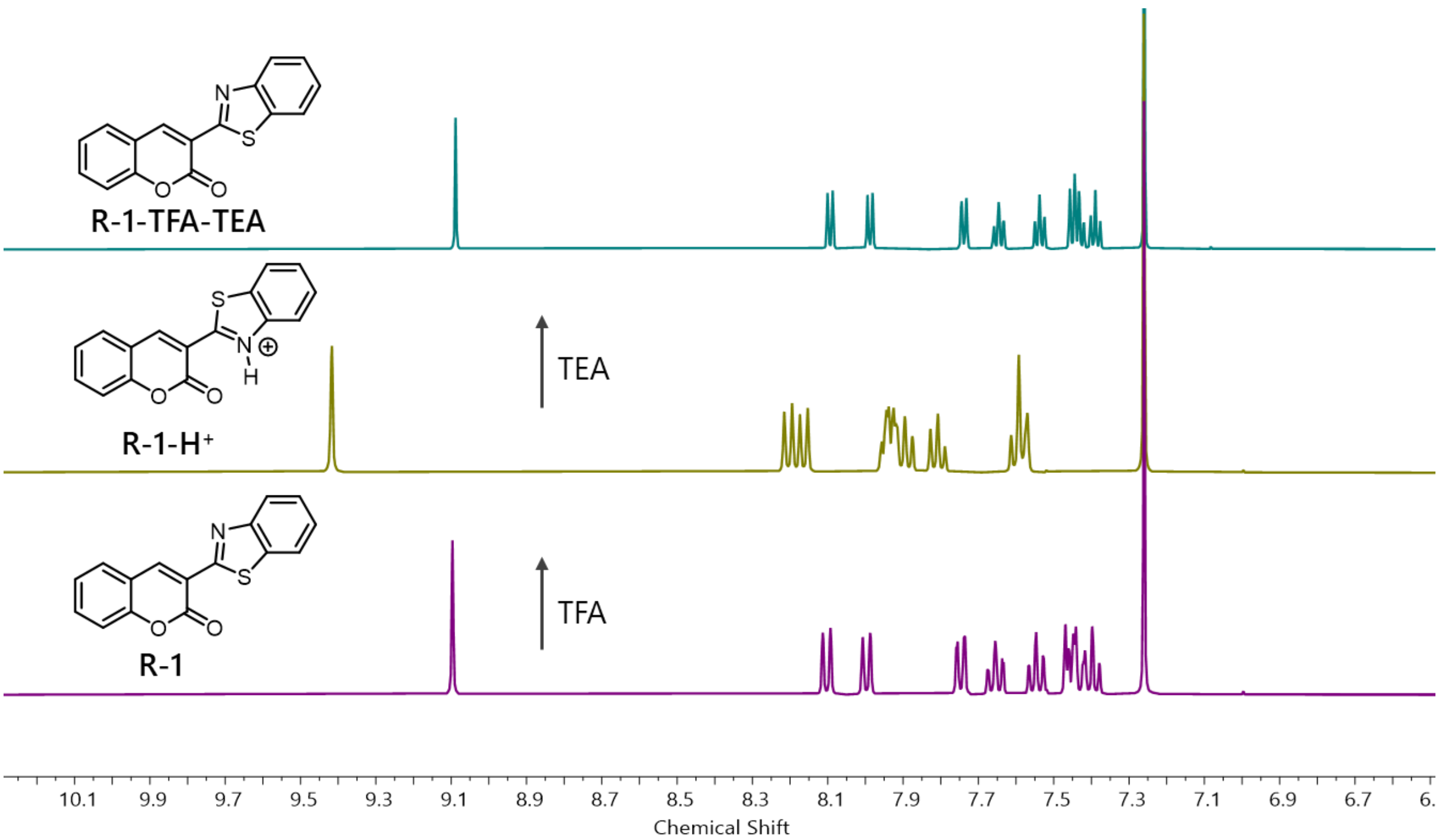

Figure S11. Structural changes and partial ^{1}H-NMR of **R1** (5 mM in CD_3Cl) showing reversible protonation process with the addition of TFA (80 equiv.) and TEA (120 equiv.).

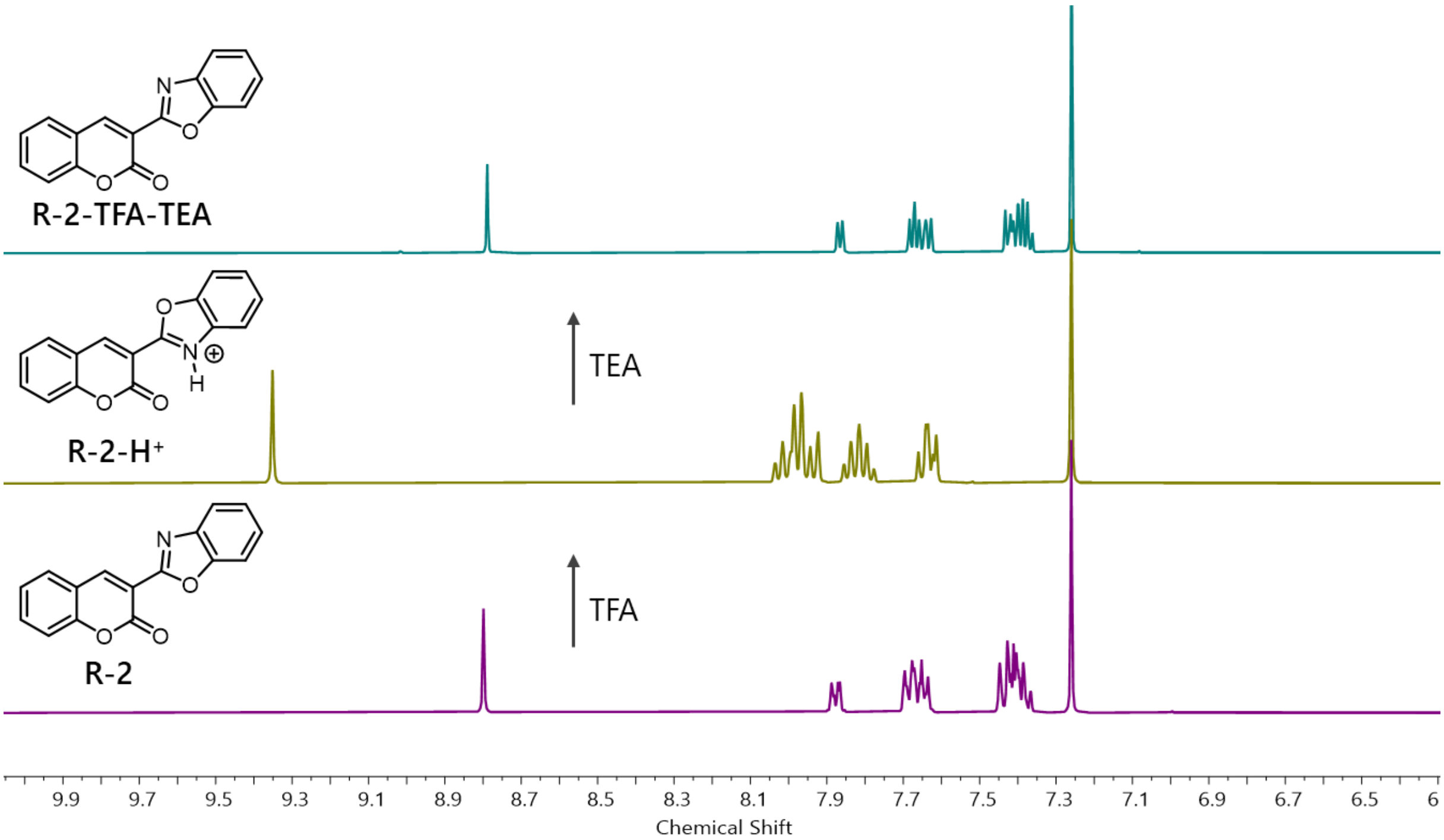

Figure S12. Structural changes and partial ^{1}H-NMR of **R2** (5 mM in CD_3Cl) showing reversible protonation process with the addition of TFA (100 equiv.) and TEA (150 equiv.).

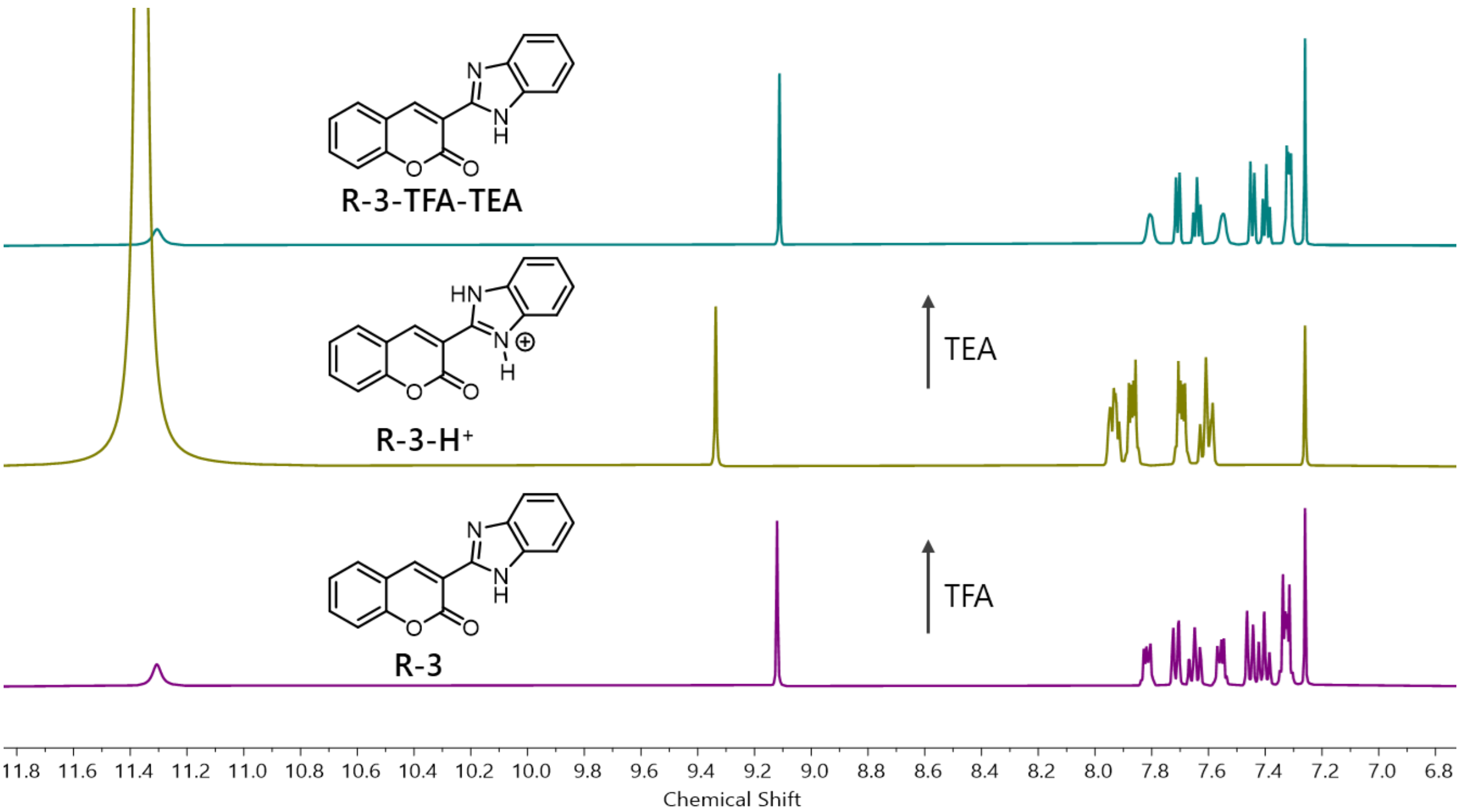

Figure S13. Structural changes and partial ^{1}H-NMR of **R3** (5 mM in CD_3Cl) showing reversible protonation process with the addition of TFA (20 equiv.) and TEA (30 equiv.).

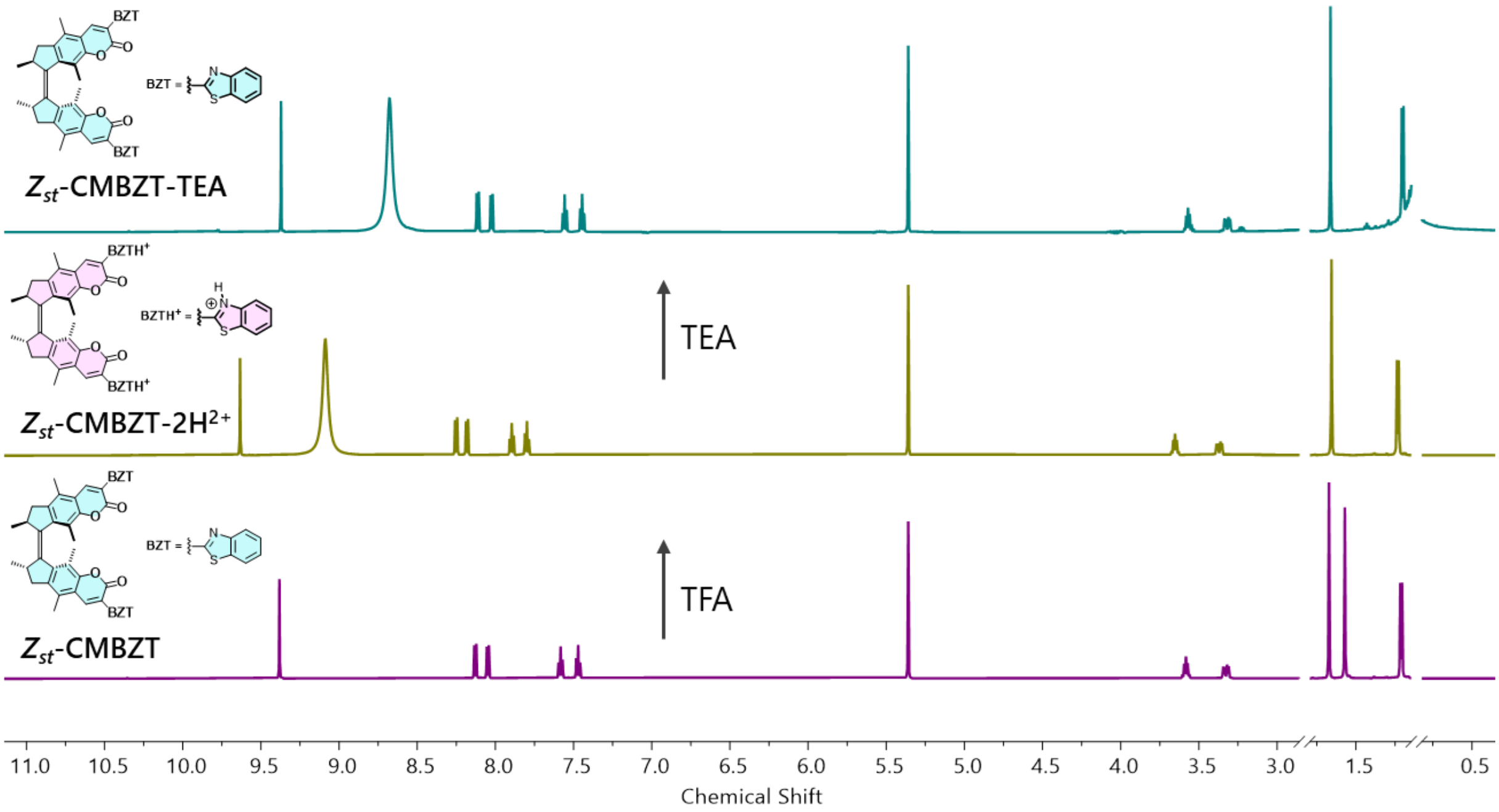

Figure S14. Structural changes and partial ^{1}H-NMR of ***Z**$_{st}$**-CMBZT*** (2 mM in CD_2Cl_2) showing reversible protonation process with the addition of TFA (150 equiv.) and TEA (200 equiv.).

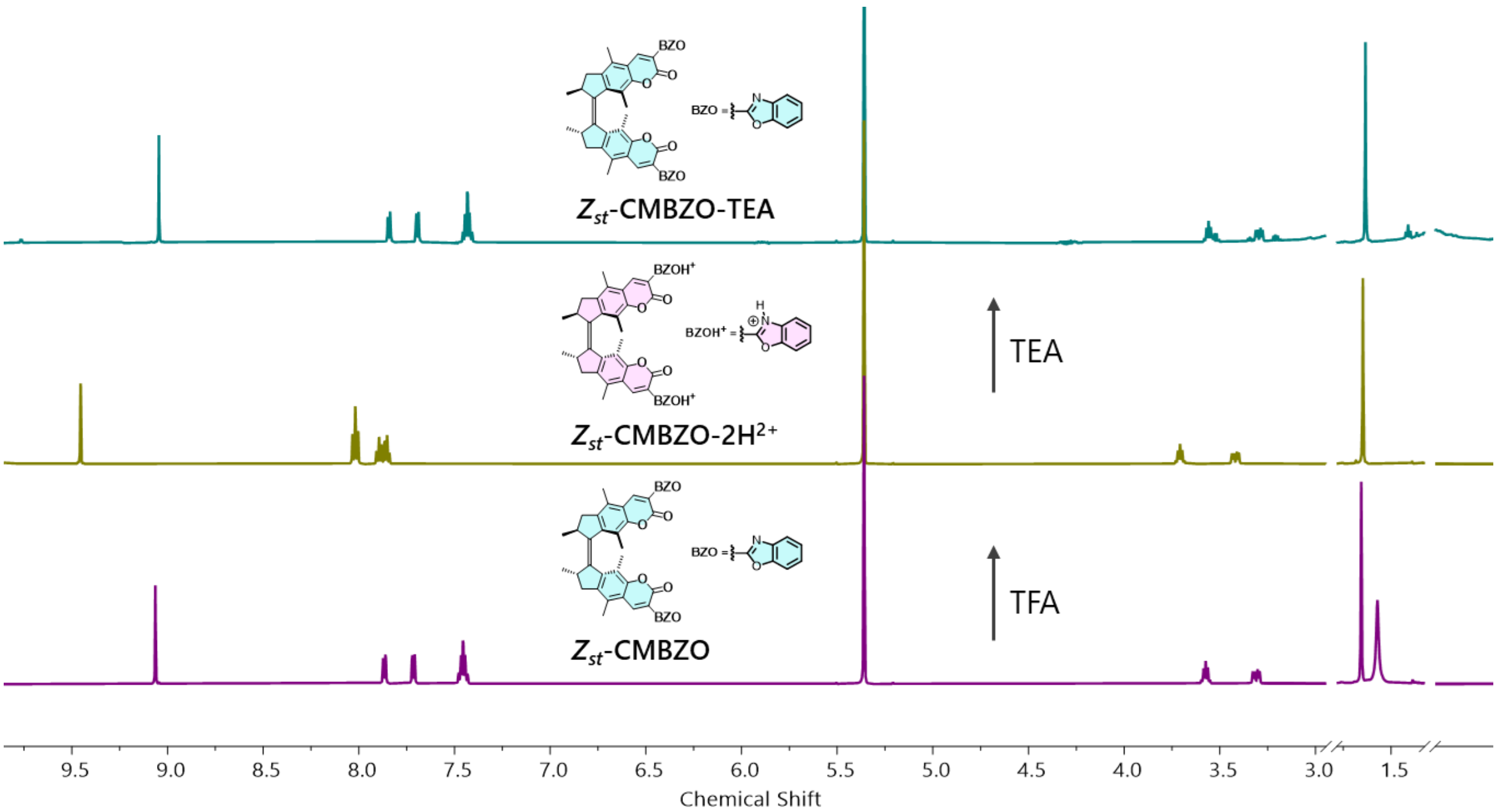

Figure S15. Structural changes and partial ^{1}H-NMR of ***Z**$_{st}$**-CMBZO*** (2 mM in CD_2Cl_2) showing reversible protonation process with the addition of TFA (250 equiv.) and TEA (400 equiv.).

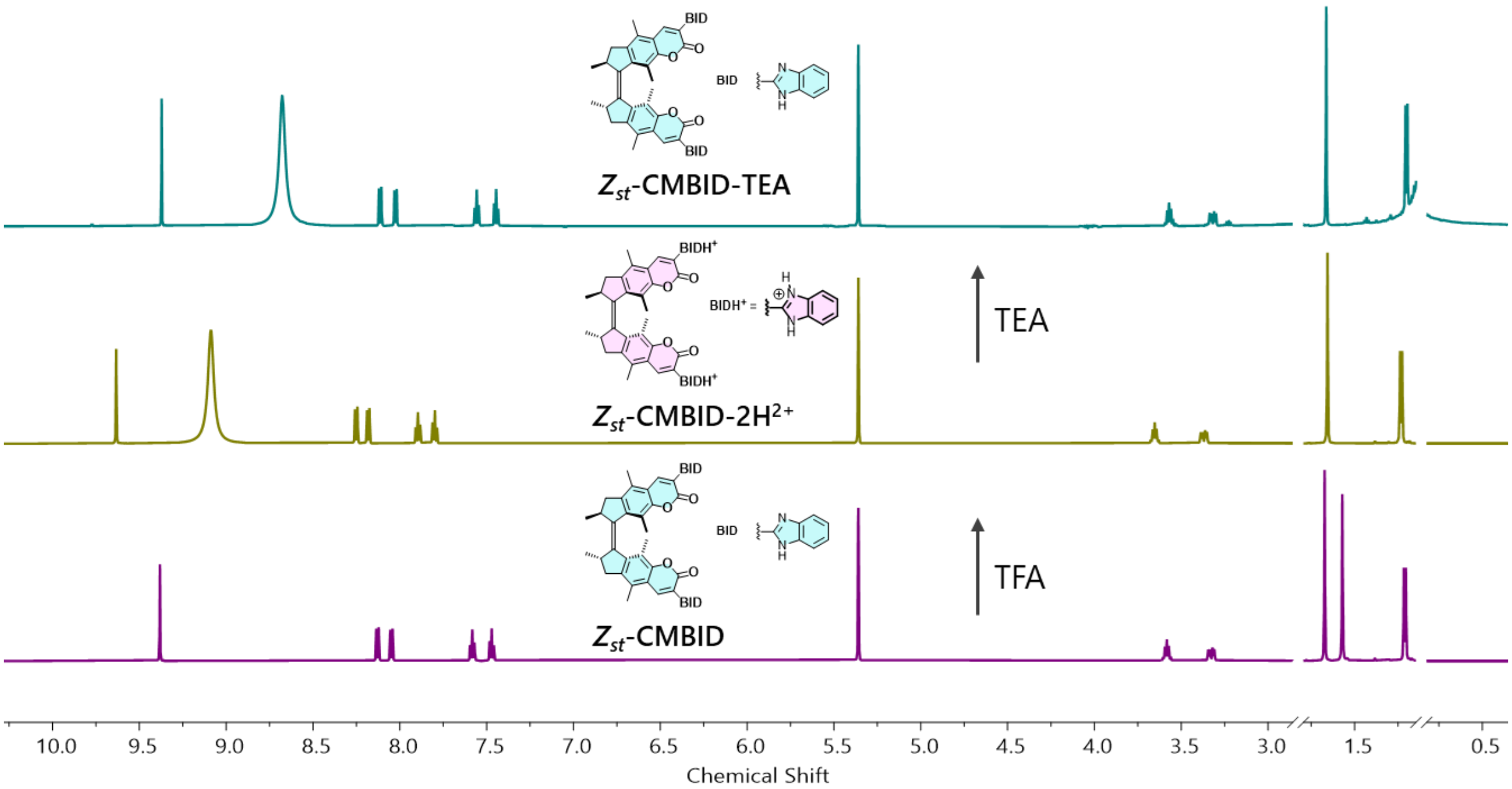

Figure S16. Structural changes and partial ^{1}H-NMR of ***Z_{st}*-CMBID** (2 mM in CD_2Cl_2) showing reversible protonation process with the addition of TFA (50 equiv.) and TEA (100 equiv.).

Table S1. Maximum absorption wavelengths of the motors **CMR** and **CMR-2H^{2+}** before and after the protonation process and the corresponding shifts of motors at different states.

Compound	*States*	λ_{max} (nm)	λ_{max}-2H^{2+} (nm)	$\Delta\lambda_{max}$ (nm)
**CMBID**	***Z_{st}***	457	505	48
	E_{st}	466	486	20
	Z_{mst}	500	547	47
**CMBZO**	***Z_{st}***	456	544	88
	E_{st}	459	526	67
	Z_{mst}	494	600	106
**CMBZT**	***Z_{st}***	470	542	72
	E_{st}	471	526	55
	Z_{mst}	522	598	76

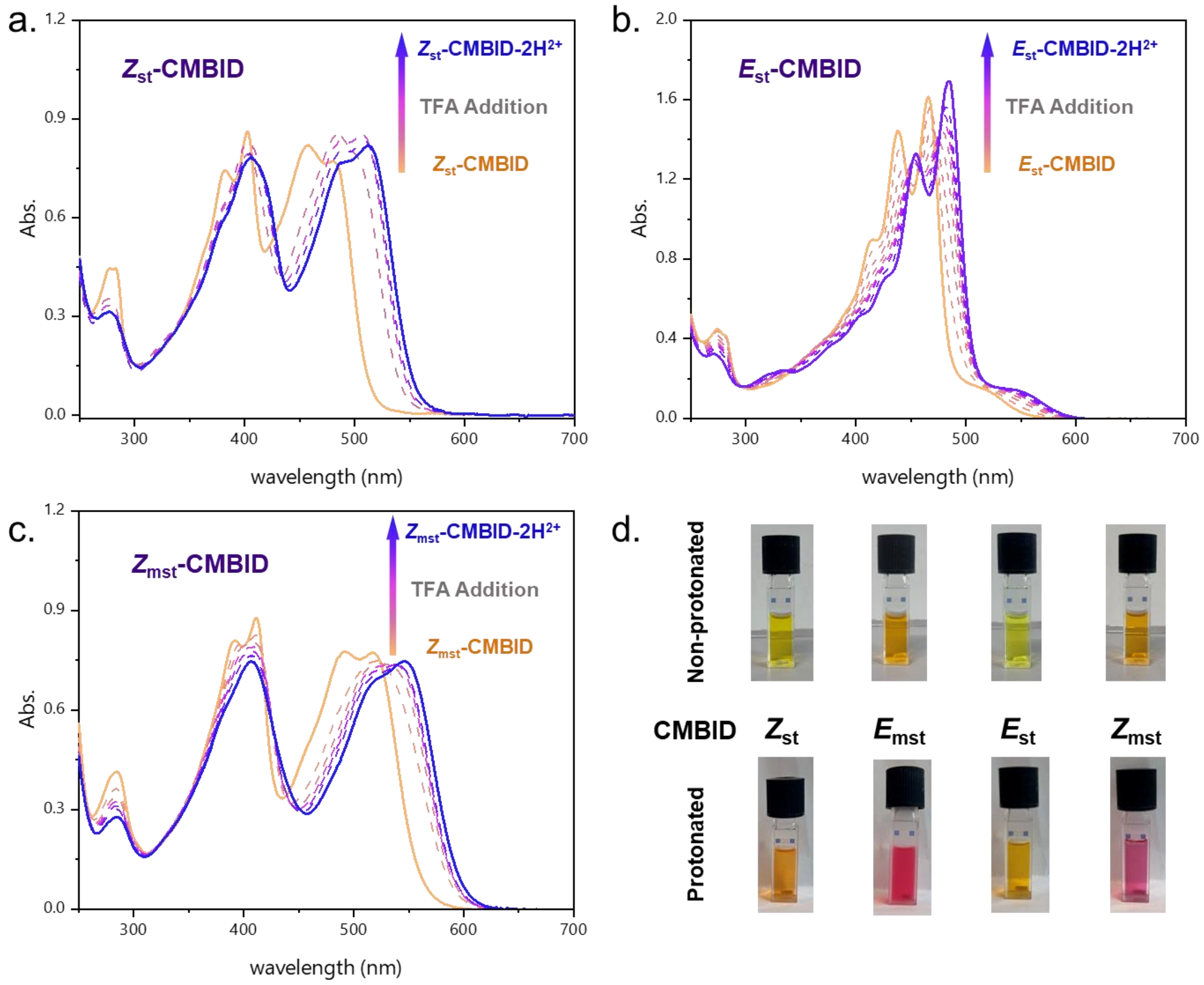

Figure S17. Protonation process of (a). **(*S*,*S*)-(*M*,*M*)-*Z*$_{st}$-CMBID**, (b). **(*S*,*S*)-(*M*,*M*)-*E*$_{st}$-CMBID** and (c). **(*S*,*S*)-(*M*,*M*)-*Z*$_{mst}$-CMBID** (2×10^{-5} M, 2 mL in DCM) with the addition of TFA (0 to 100 equiv, 0.1 M diluted in DCM) in DCM solution recorded by UV/vis spectroscopy. (d). The color changes corresponding to the protonation process.

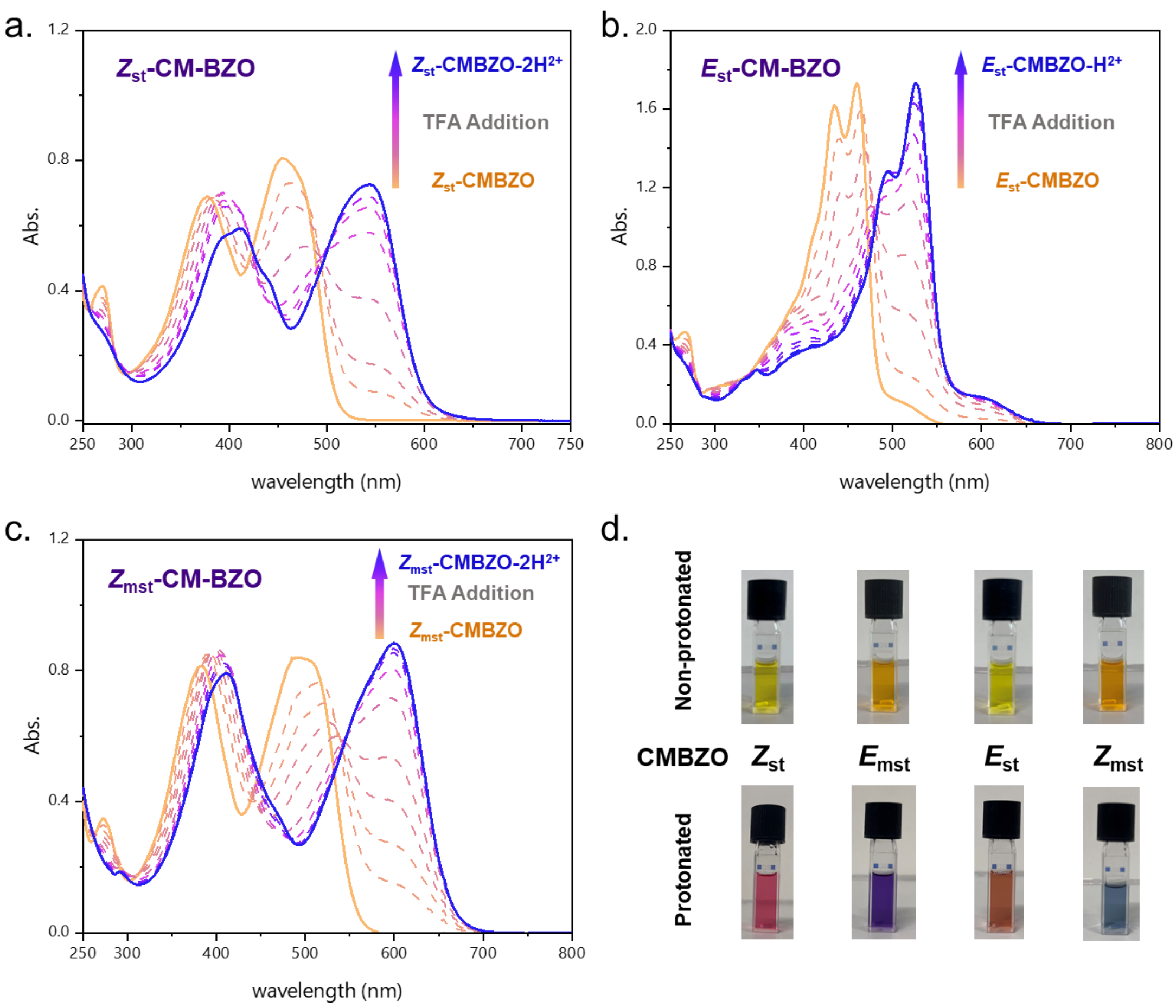

Figure S18. Protonation process of (a). **(*S*,*S*)-(*M*,*M*)-*Z*$_{st}$-CMBZO**, (b). **(*S*,*S*)-(*M*,*M*)-*E*$_{st}$-CMBZO** and (c). **(*S*,*S*)-(*M*,*M*)-*Z*$_{mst}$-CMBZO** with the addition of TFA (0 to 1000 equiv, 0.1 M, diluted in DCM) in DCM solution recorded by UV/vis spectroscopy. (d). The color changes corresponding to the protonation process.

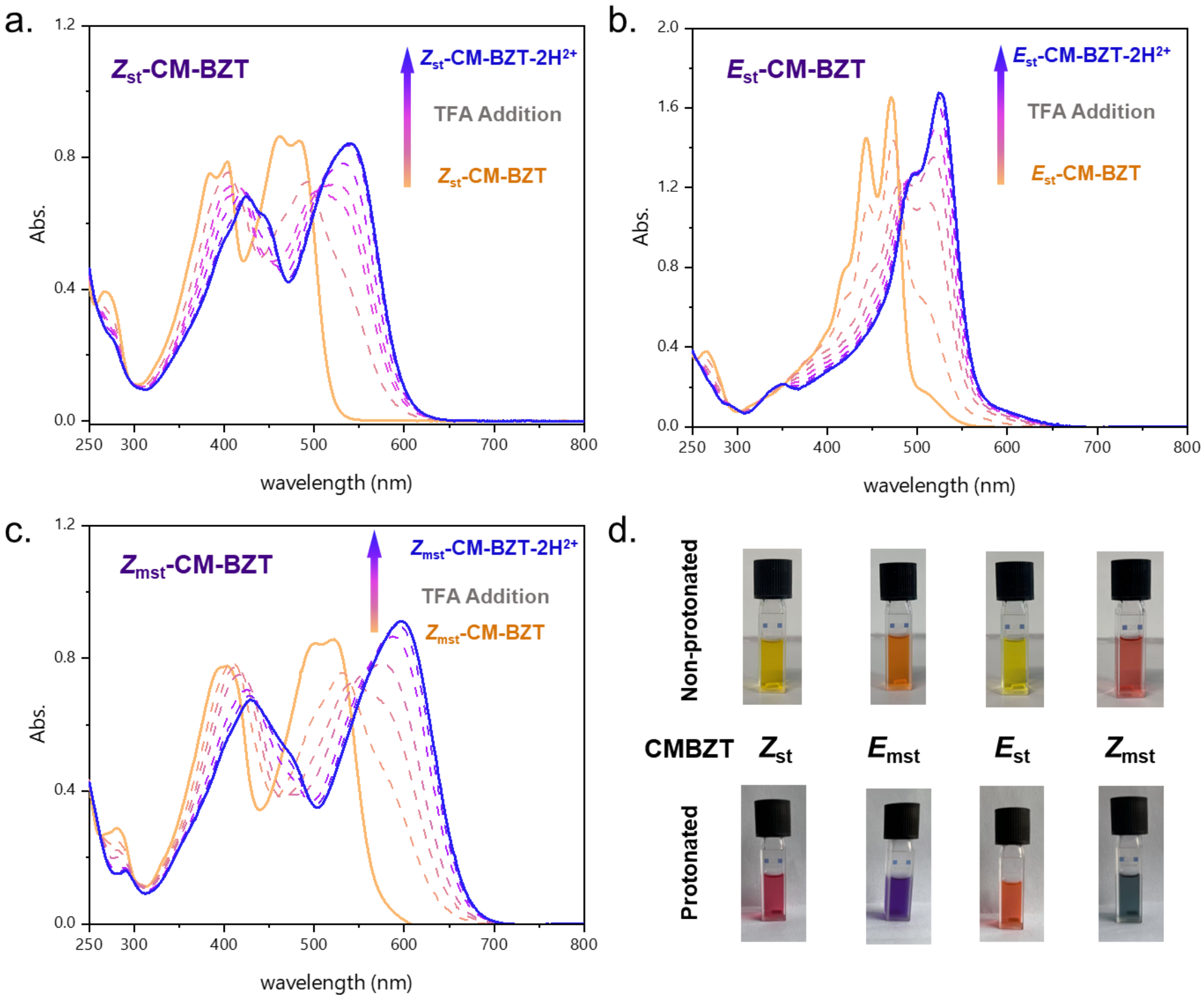

Figure S19. Protonation process of (a). **(*S*,*S*)-(*M*,*M*)-Z_{st}-CMBZT**, (b). **(*S*,*S*)-(*M*,*M*)-E_{st}-CMBZT** and (c). **(*S*,*S*)-(*M*,*M*)-Z_{mst}-CMBZT** with the addition of TFA (0 to 800 equiv, 0.1 M, diluted in DCM) in DCM solution recorded by UV/vis spectroscopy. (d). The color changes corresponding to the protonation process.

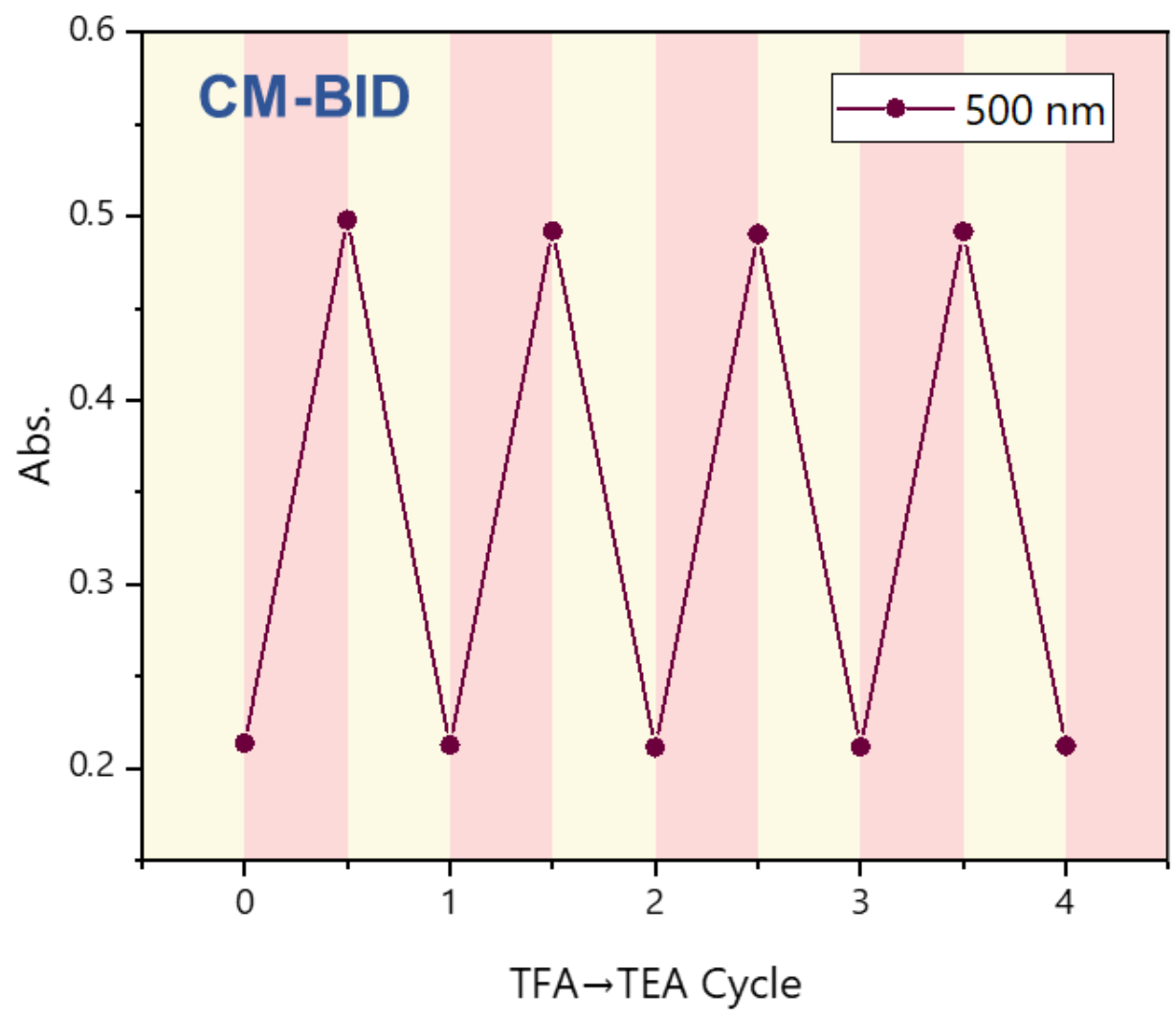

Figure S20. Fatigue studies of **(*S*,*S*)-(*M*,*M*)-*Z*$_{st}$-CMBID** (DCM, 1.0*10^{-5} M) before and after addition of TFA and TEA (1.5 equiv with respect to TFA).

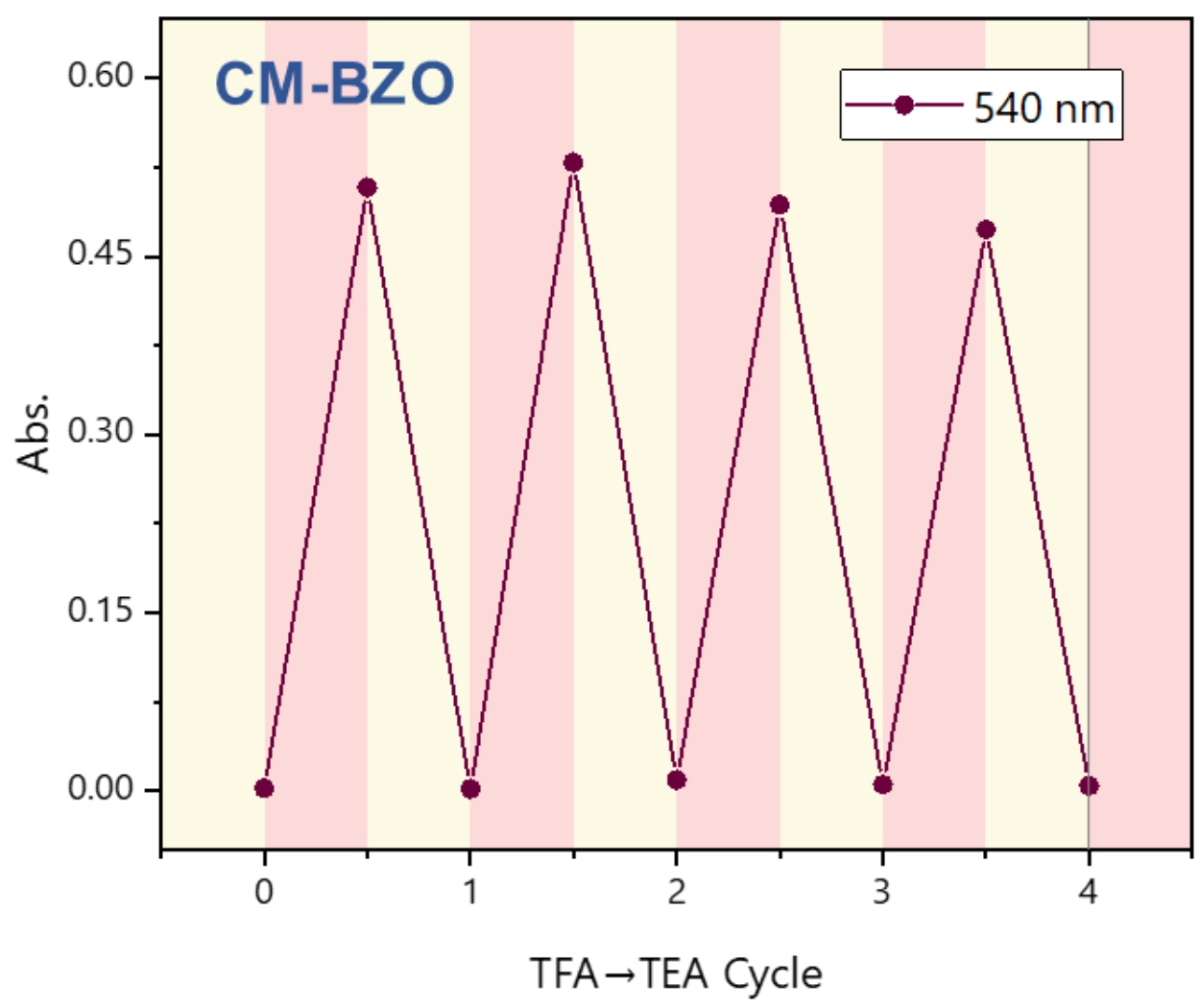

Figure S21. Fatigue studies of **(*S*,*S*)-(*M*,*M*)-*Z*$_{st}$-CMBZO** (DCM, 1.0*10^{-5} M) before and after addition of TFA and TEA (1.5 equiv with respect to TFA).

7. Motor rotatory cycles recorded by UV/vis absorption spectroscopy after protonation

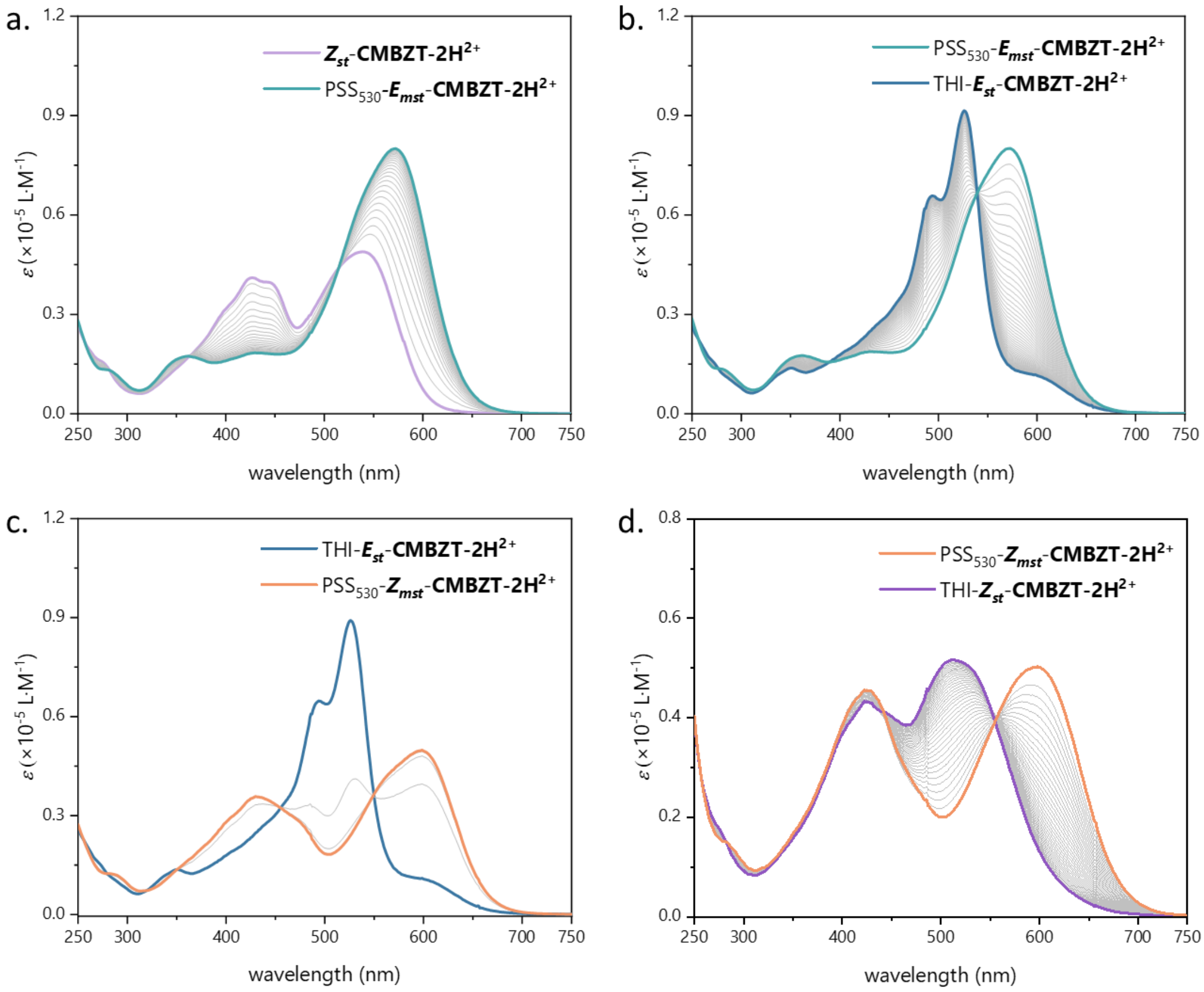

Figure S22. Unidirectional processes of **CMBZT-2H^{2+}** recorded by UV/vis absorption spectroscopy. **a.** UV-vis spectra of ***Z$_{st}$*-CMBZT-2H^{2+}** (light purple, 1.5*10^{-5} M in DCM) upon 530 nm light irradiation to get PSS$_{530}$-***E$_{mst}$*-CMBZT-2H^{2+}** (light green line, −12 ºC, 1.5*10^{-5} M in DCM). **b.** maintaining in the dark for 2 h to reach THI-***E$_{st}$*-CMBZT-2H^{2+}** (blue line, −12 ºC, 1.5*10^{-5} M in DCM). **c.** subsequent irradiating with 530 nm light to get PSS$_{530}$-***Z$_{mst}$*-CMBZT-2H^{2+}** (orange line, −12 ºC, 1.5*10^{-5} M in DCM). **d.** finally keeping in the dark for 2 h to recover THI-***Z$_{st}$*-CMBZT-2H^{2+}** **(**purple line, 70 ºC, 1.5*10^{-5} M in DCE**)**.

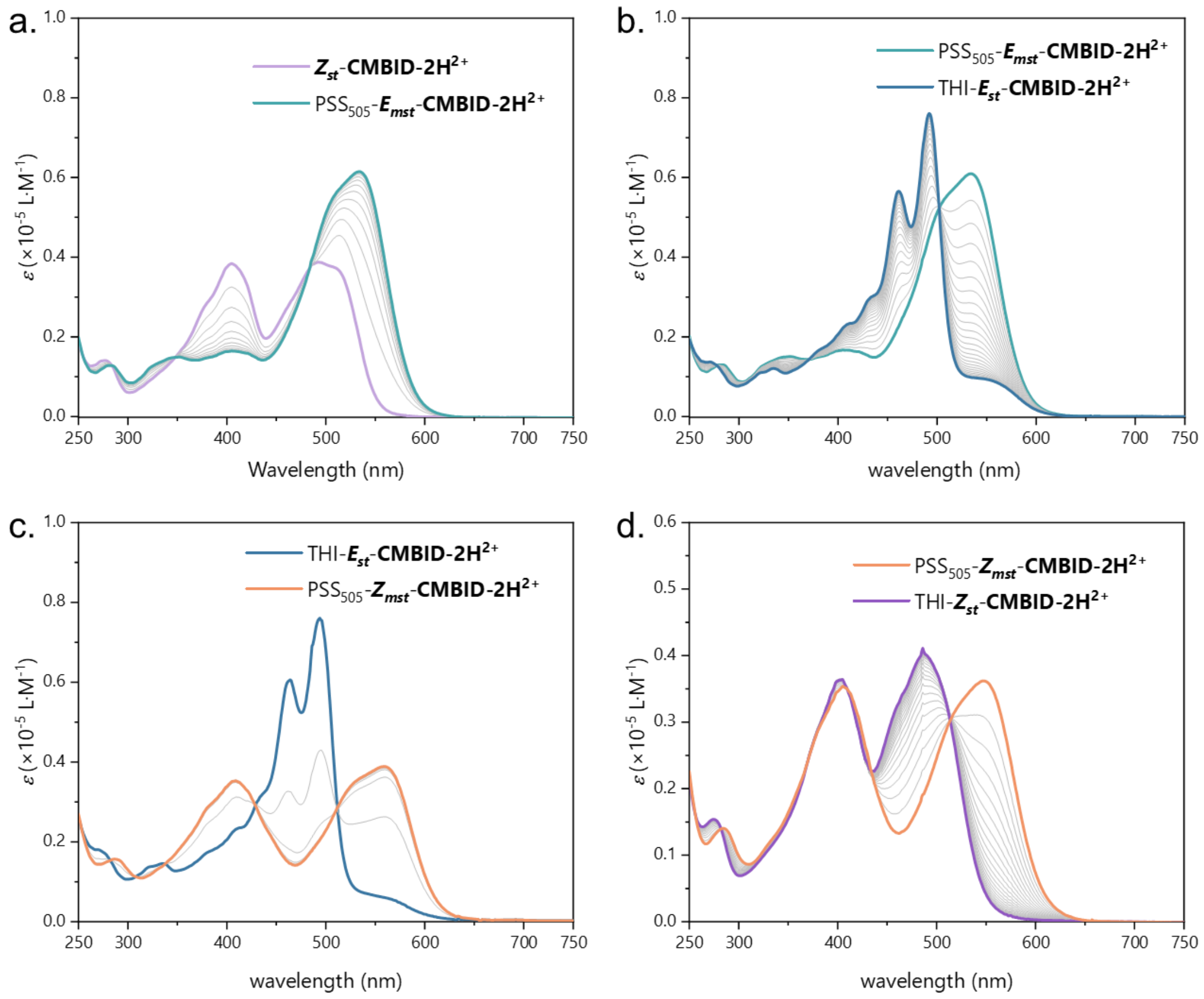

Figure S23. Unidirectional processes of **CMBID-2H^{2+}** recorded by UV/vis absorption spectroscopy. **a.** UV-vis spectra of ***Z$_{st}$*-CMBID-2H^{2+}** (light purple, 1.0*10^{-5} M in DCM) upon 505 nm light irradiation to get PSS$_{505}$-***E$_{mst}$*-CMBID-2H^{2+}** (light green line, − 12 ºC, 1.0*10^{-5} M in DCM). **b.** keeping in the dark for 2 h to reach THI-***E$_{st}$*-CMBID-2H^{2+}** (blue line, − 12 ºC, 1.0*10^{-5} M in DCM). **c.** subsequent irradiating with 505 nm light to get PSS$_{505}$-***Z$_{mst}$*-CMBID-2H^{2+}** (orange line, − 12 ºC, 1.0*10^{-5} M in DCM). **d.** finally keeping in the dark for 2 h to recover THI-***Z$_{st}$*-CMBID-2H^{2+}** **(**purple line, 70 ºC, 1.0*10^{-5} M in DCE**)**.

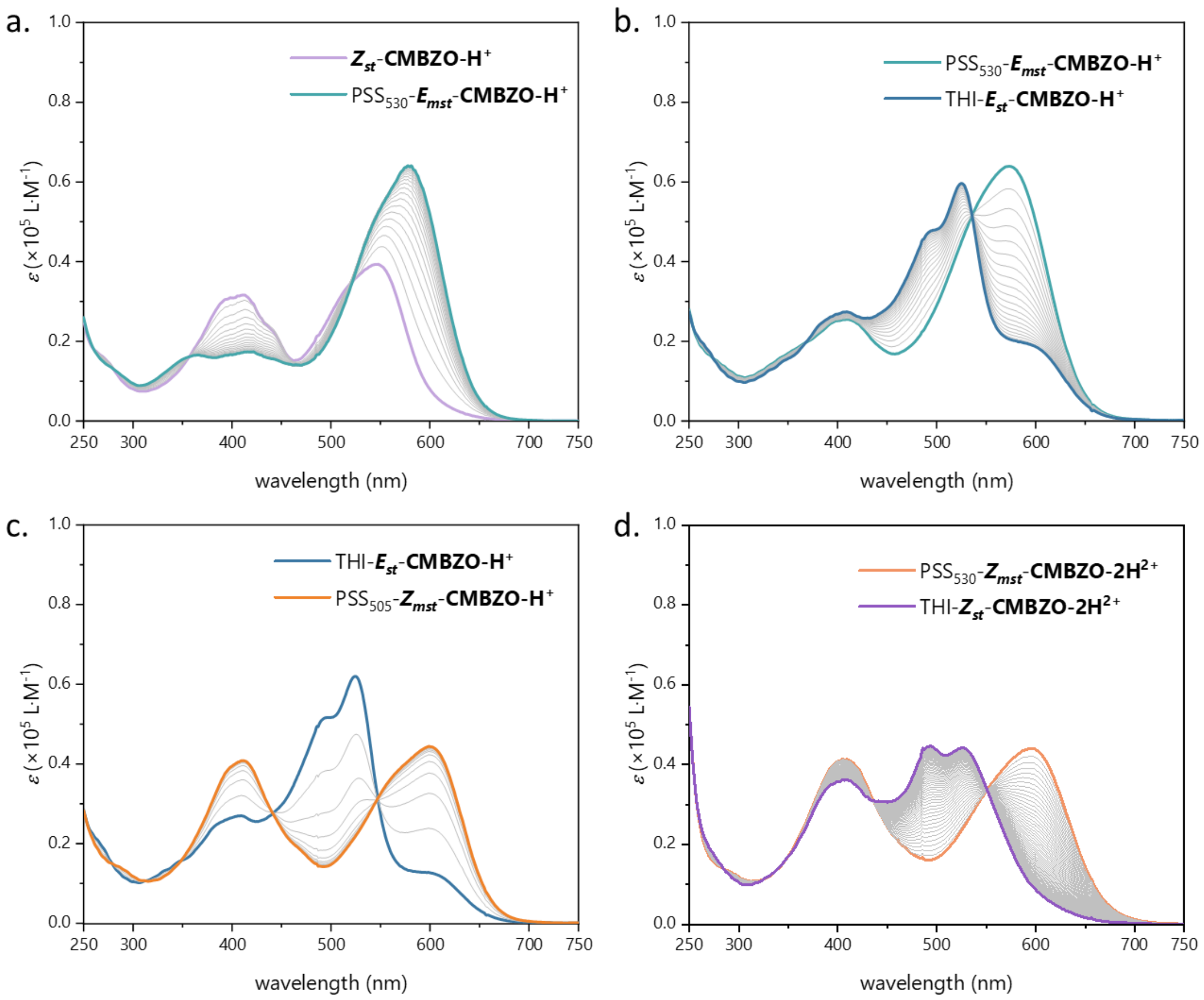

Figure S24. Unidirectional processes of **CMBZO-2H^{2+}** recorded by UV/vis absorption spectroscopy. **a.** UV-vis spectra of ***Z$_{st}$*-CMBZO-2H^{2+}** (light purple, 1.5*10^{-5} M in DCM) upon 530 nm light irradiation to get PSS$_{530}$-***E$_{mst}$*-CMBZT-2H^{2+}** (light green line, − 12 ºC, 1.5*10^{-5} M in DCM). **b.** keeping in the dark for 2 h to reach THI-***E$_{st}$*-CMBZT-2H^{2+}** (blue line, − 12 ºC, 1.5*10^{-5} M in DCM). **c.** subsequent irradiating with 530 nm light to get PSS$_{530}$-***Z$_{mst}$*-CMBZT-2H^{2+}** (orange line, − 12 ºC, 1.5*10^{-5} M in DCM). **d.** finally keeping in the dark for 2 h to recover THI-***Z$_{st}$*-CMBZT-2H^{2+}** **(**purple line, 70 ºC, 1.5*10^{-5} M in DCE**)**.

8. Kinetic studies of thermal helix inversion steps of motors after protonation

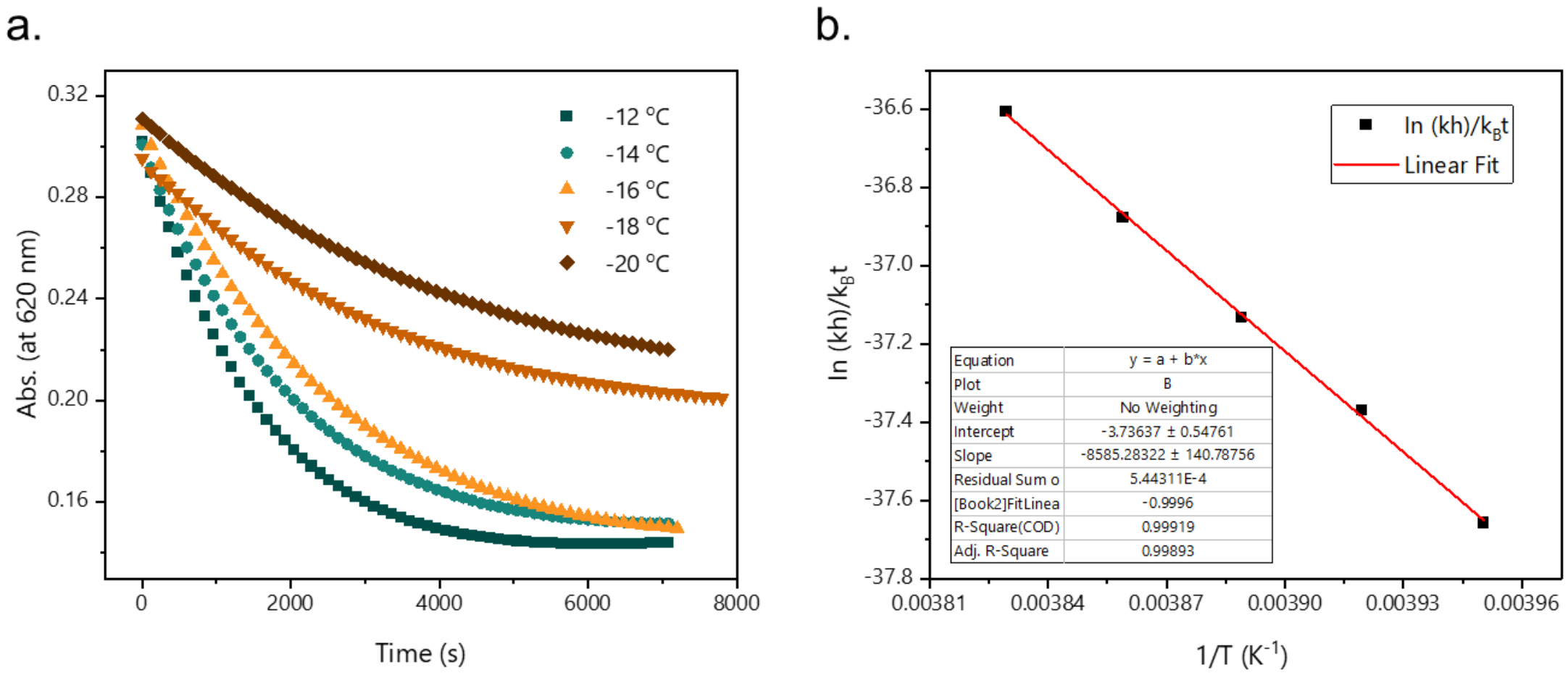

Figure S25. Kinetic studies of thermal helix inversion steps of PSS_{530}-***E****mst*-**CMBZT-2H**$^{2+}$. **a.** Changes of the absorption values at 620 nm of (DCM, 1.5*10^{-5} M) at different temperature (-20 ºC to -12 ºC), which shows the thermal helix inversion (THI) process with the formation of predominantly THI-***E****st*-**CMBZT-2H**$^{2+}$ motor. **b.** Eyring plot of THI step from ***E****mst*-**CMBZT-2H**$^{2+}$ to ***E****st*-**CMBZT-2H**$^{2+}$ with insertion of the linear fitting parameters of the Eyring analysis.

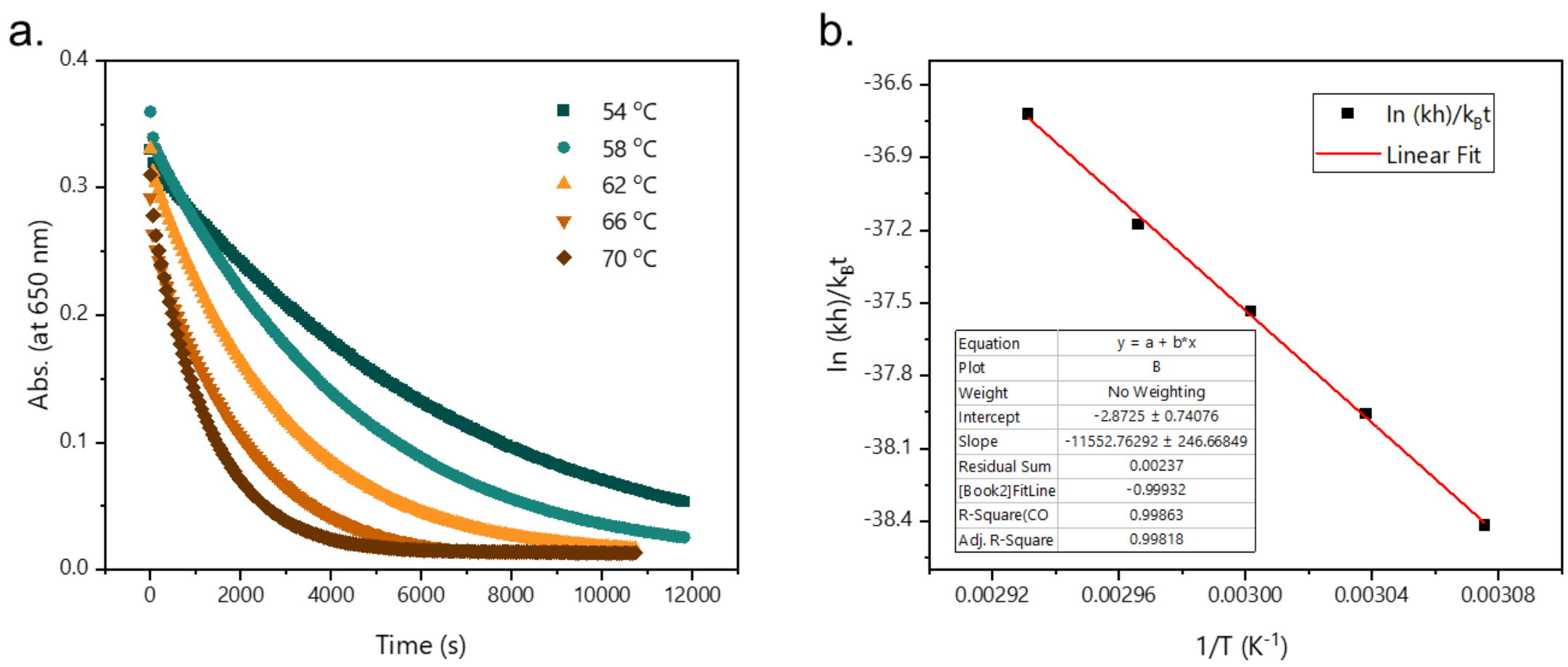

Figure S26. Kinetic studies of thermal helix inversion steps of PSS_{530}-***Z****mst*-**CMBZT-2H**$^{2+}$. **a.** Changes of the absorption values at 650 nm of (DCE, 1.5*10^{-5} M) at different temperature (54 ºC to 70 ºC), which shows the thermal helix inversion (THI) process with the formation of predominantly THI-***Z****st*-**CMBZT-2H**$^{2+}$ motor. **b.** Eyring plot of THI step from ***Z****mst*-**CMBZT-2H**$^{2+}$ to ***Z****st*-**CMBZT-2H**$^{2+}$ with insertion of the linear fitting parameters of the Eyring analysis.

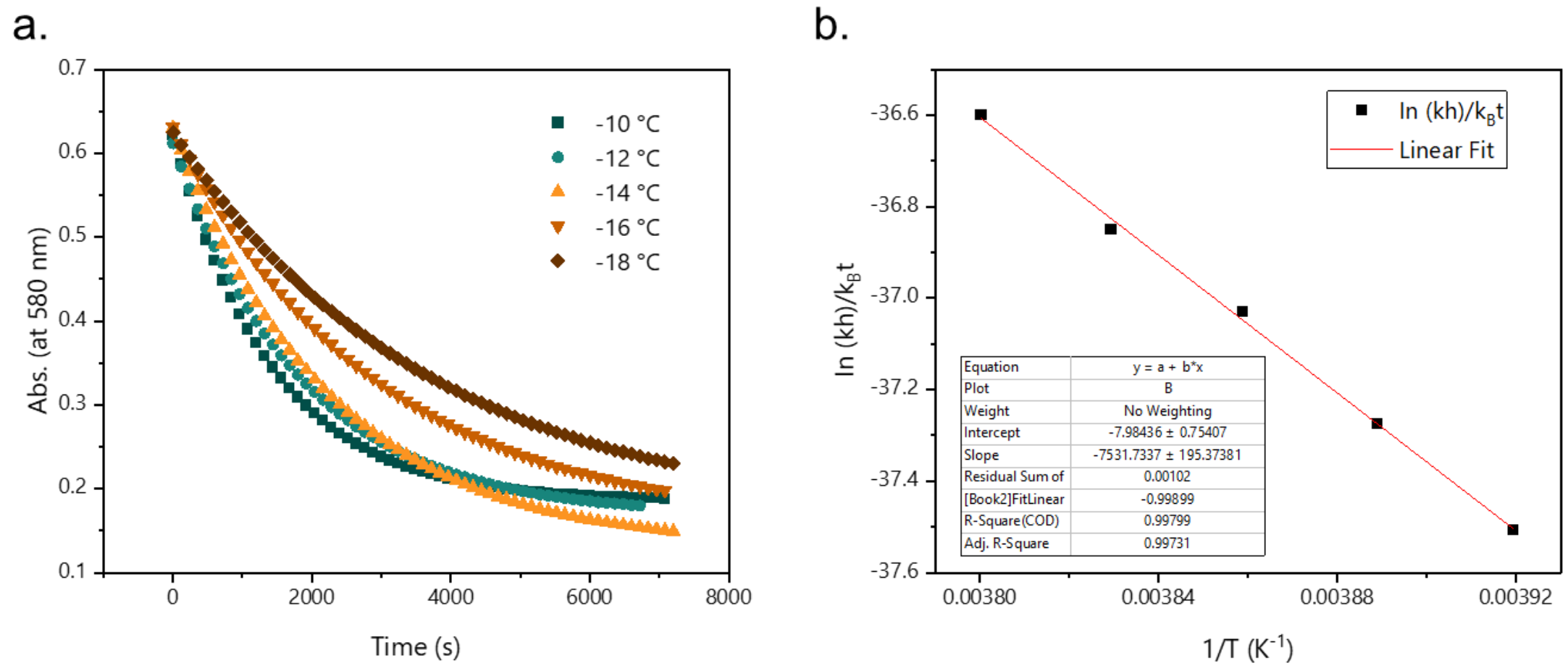

Figure S27. Kinetic studies of thermal helix inversion steps of PSS$_{530}$-***E$_{mst}$*-CMBZO-2H^{2+}**. **a.** Changes of the absorption values at 580 nm of (DCM, 1.5*10^{-5} M) at different temperature (-18 ºC to -10 ºC), which shows the thermal helix inversion (THI) process with the formation of predominantly THI-***E$_{st}$*-CMBZO-2H^{2+}** motor. **b.** Eyring plot of THI step from ***E$_{mst}$*-CMBZO-2H^{2+}** to ***E$_{st}$*-CMBZO-2H^{2+}** with insertion of the linear fitting parameters of the Eyring analysis.

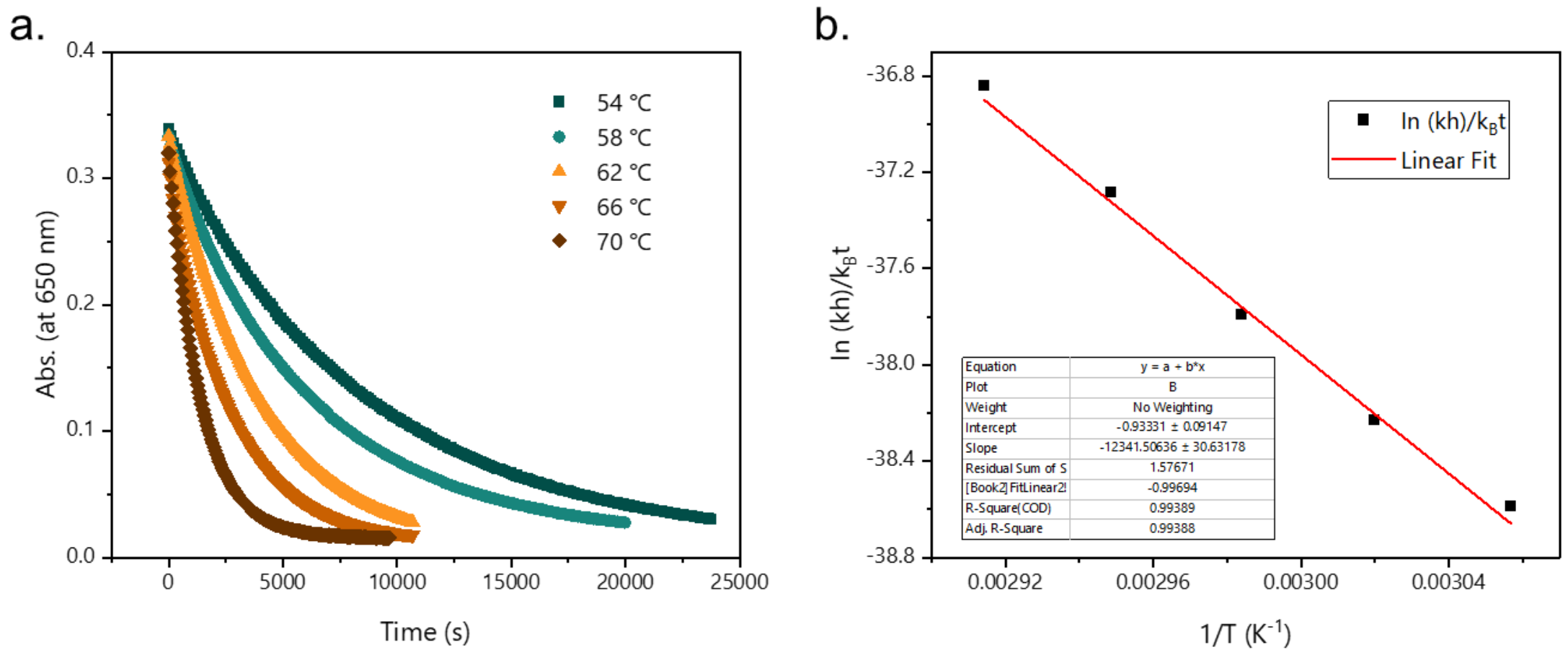

Figure S28. Kinetic studies of thermal helix inversion steps of PSS$_{530}$-***Z$_{mst}$*-CMBZO-2H^{2+}**. **a.** Changes of the absorption values at 650 nm of (DCE, 1.5*10^{-5} M) at different temperature (54 ºC to 70 ºC), which shows the thermal helix inversion (THI) process with the formation of predominantly THI-***Z$_{st}$*-CMBZO-2H^{2+}** motor. **b.** Eyring plot of THI step from ***Z$_{mst}$*-CMBZO-2H^{2+}** to ***Z$_{st}$*-CMBZO-2H^{2+}** with insertion of the linear fitting parameters of the Eyring analysis.

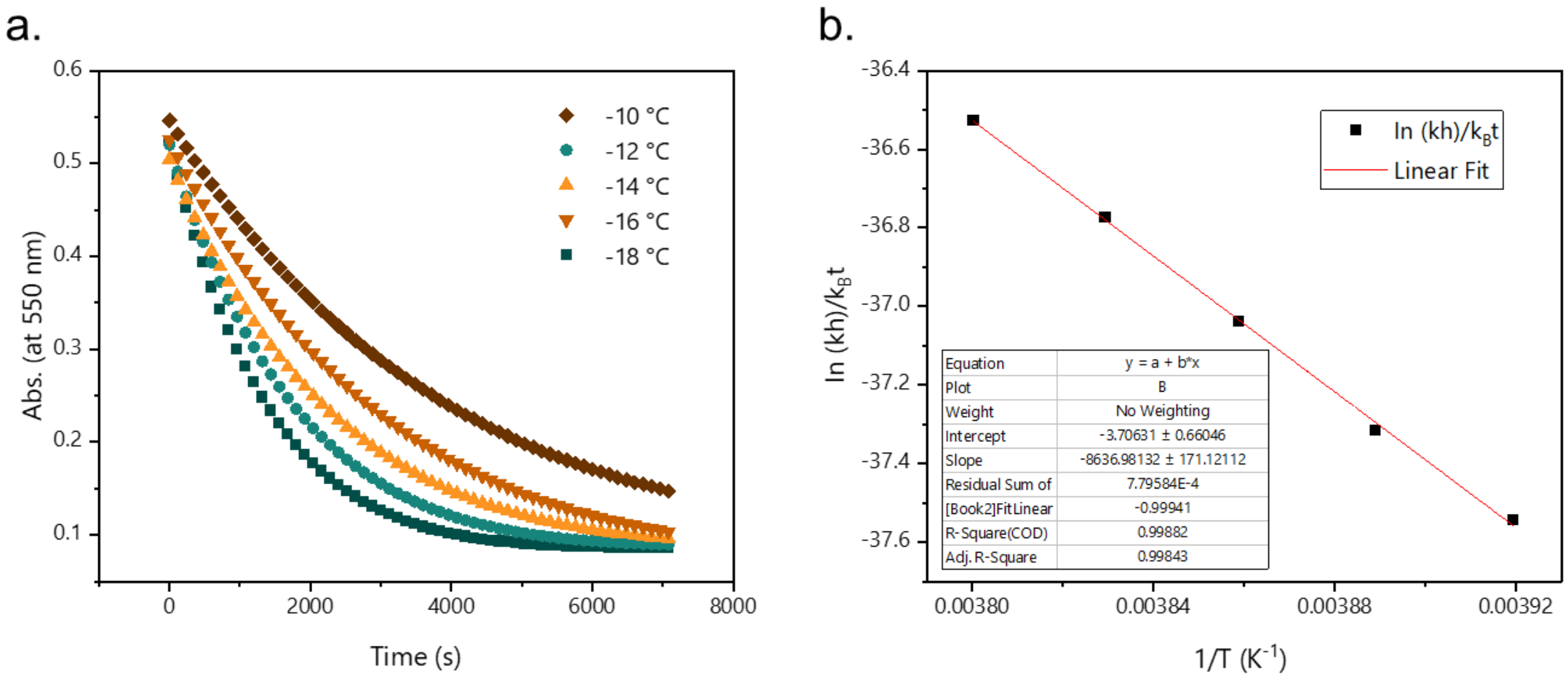

Figure S29. Kinetic studies of thermal helix inversion steps of PSS$_{505}$-***E**$_{mst}$*-**CMBID-2H**$^{2+}$. **a.** Changes of the absorption values at 550 nm of (DCM, 1.5*10^{-5} M) at different temperature (-18 ºC to -10 ºC), which shows the thermal helix inversion (THI) process with the formation of predominantly THI-***E**$_{st}$*-**CMBID-2H**$^{2+}$ motor. **b.** Eyring plot of THI step from ***E**$_{mst}$*-**CMBID-2H**$^{2+}$ to ***E**$_{st}$*-**CMBID-2H**$^{2+}$ with insertion of the linear fitting parameters of the Eyring analysis.

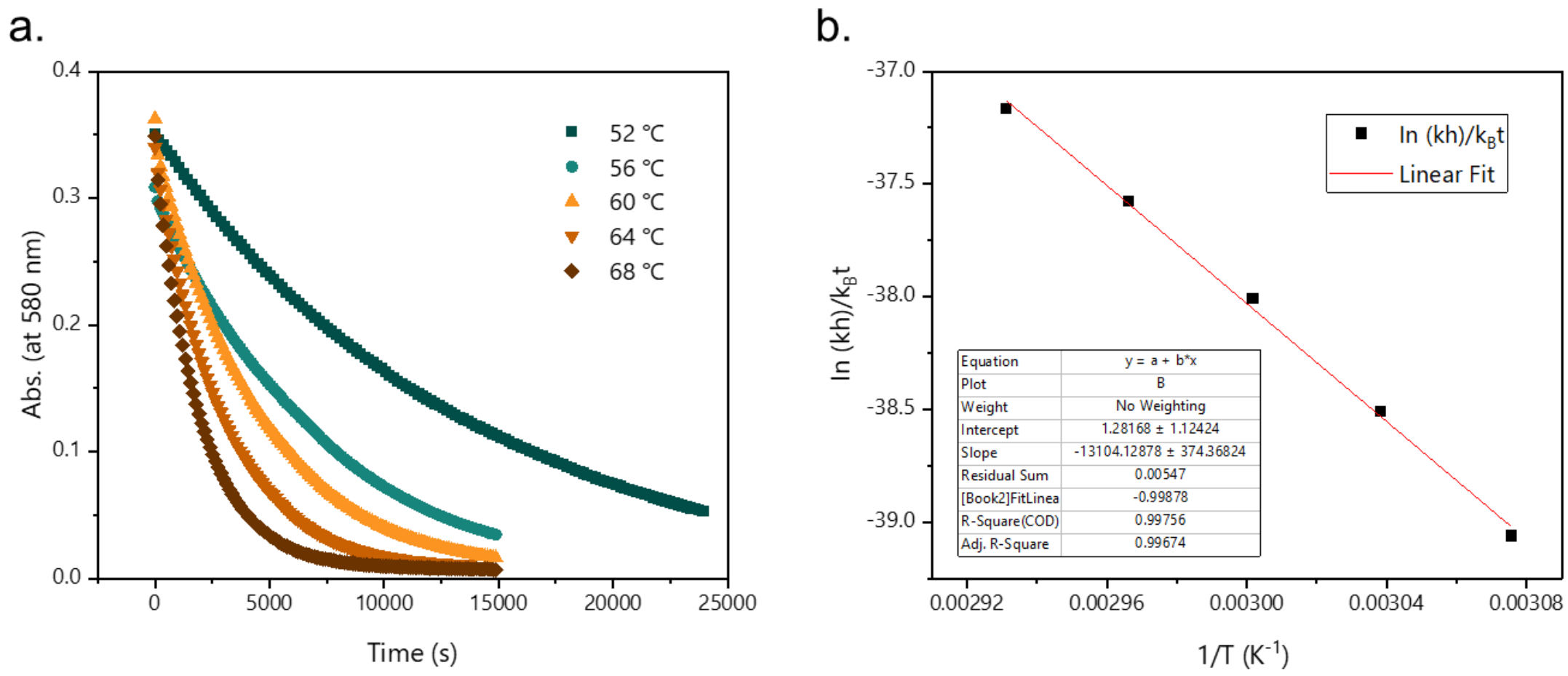

Figure S30. Kinetic studies of thermal helix inversion steps of PSS$_{505}$-***Z**$_{mst}$*-**CMBID-2H**$^{2+}$. **a.** Changes of the absorption values at 580 nm of (DCE, 1.5*10^{-5} M) at different temperature (52 ºC to 68 ºC), which shows the thermal helix inversion (THI) process with the formation of predominantly THI-***Z**$_{st}$*-**CMBID-2H**$^{2+}$ motor. **b.** Eyring plot of THI step from ***Z**$_{mst}$*-**CMBID-2H**$^{2+}$ to ***Z**$_{st}$*-**CMBID-2H**$^{2+}$ with insertion of the linear fitting parameters of the Eyring analysis.

9. Unidirectional rotation and PSS ratio determination of motors

To estimate the photo-stationary state ratios of the motors at each photochemical reaction process, in-situ NMR irradiation experiments were conducted using an optical fiber connecting with the corresponding lamps. The ratio was by finally calculated by comparison the integration of the generated meta-stable isomers ($\boldsymbol{Z_{st}}$ to $\boldsymbol{E_{mst}}$ and $\boldsymbol{E_{st}}$ to $\boldsymbol{Z_{mst}}$, respectively). It should be noted that during the generation of the second photostationary states (from $\boldsymbol{E_{st}}$ to $\boldsymbol{Z_{mst}}$), the unconverted $\boldsymbol{Z_{st}}$ (less than 5%) isomers from the last step would also conduct the photochemical reaction, with the generation of the $\boldsymbol{E_{mst}}$ isomers. The calculation of the secondary PSS ratio was then conducted only based on the ratio of $\boldsymbol{Z_{mst}}$ to $\boldsymbol{E_{st}}$. All the in-situ irradiation experiments were performed at – 45 °C to avoid the potential THI process during the irradiation process.

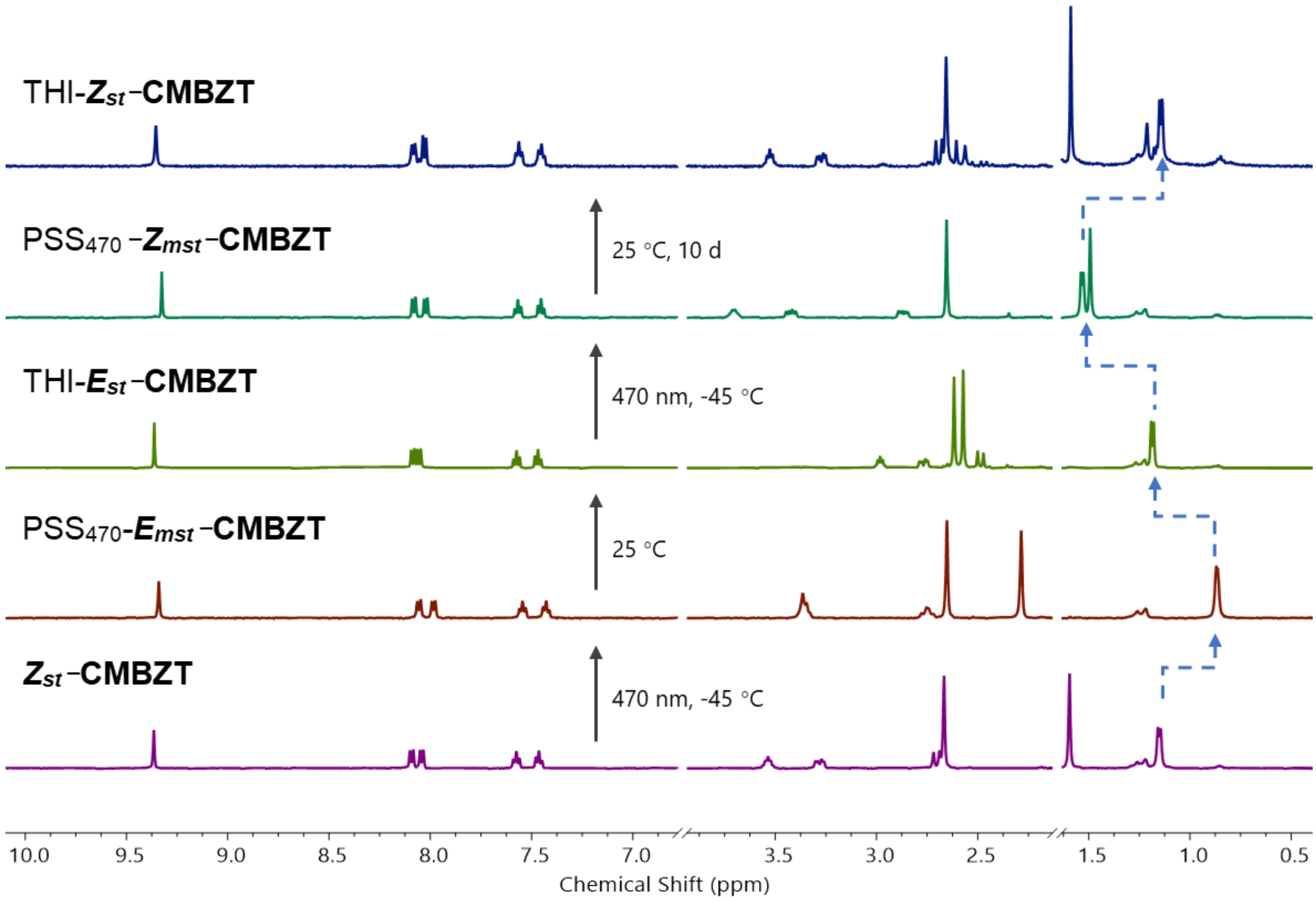

Figure S31. Rotary cycle of motor **CMBZT** followed by ^{1}H NMR (500 MHz, CD_2Cl_2, – 45 °C) starting from ***Z**$_{st}$***-CMBZT** (bottom purple line) to PSS$_{470}$-***E**$_{mst}$***-CMBZT** (red line) with 470 nm light irradiation at – 45 °C, followed by heating to room temperature for THI process to generate THI-***E**$_{st}$***-CMBZT** (green line); and subsequent irradiation with 470 nm light at – 45 °C to PSS$_{470}$-***Z**$_{mst}$***-CMBZT** (teal line). Finally, the sample was kept at room temperature for 10 d to regenerate ***Z**$_{st}$***-CMBZT** (THI-***Z**$_{st}$***-CMBZT** top blue line). All ^{1}H NMR measurements were performed at –45 °C to ensure the chemical shifts were not influenced by temperature. Dashed arrows were used to direct attention to the changes in the chemical shifts (*δ*, ppm) of the characteristic resonances.

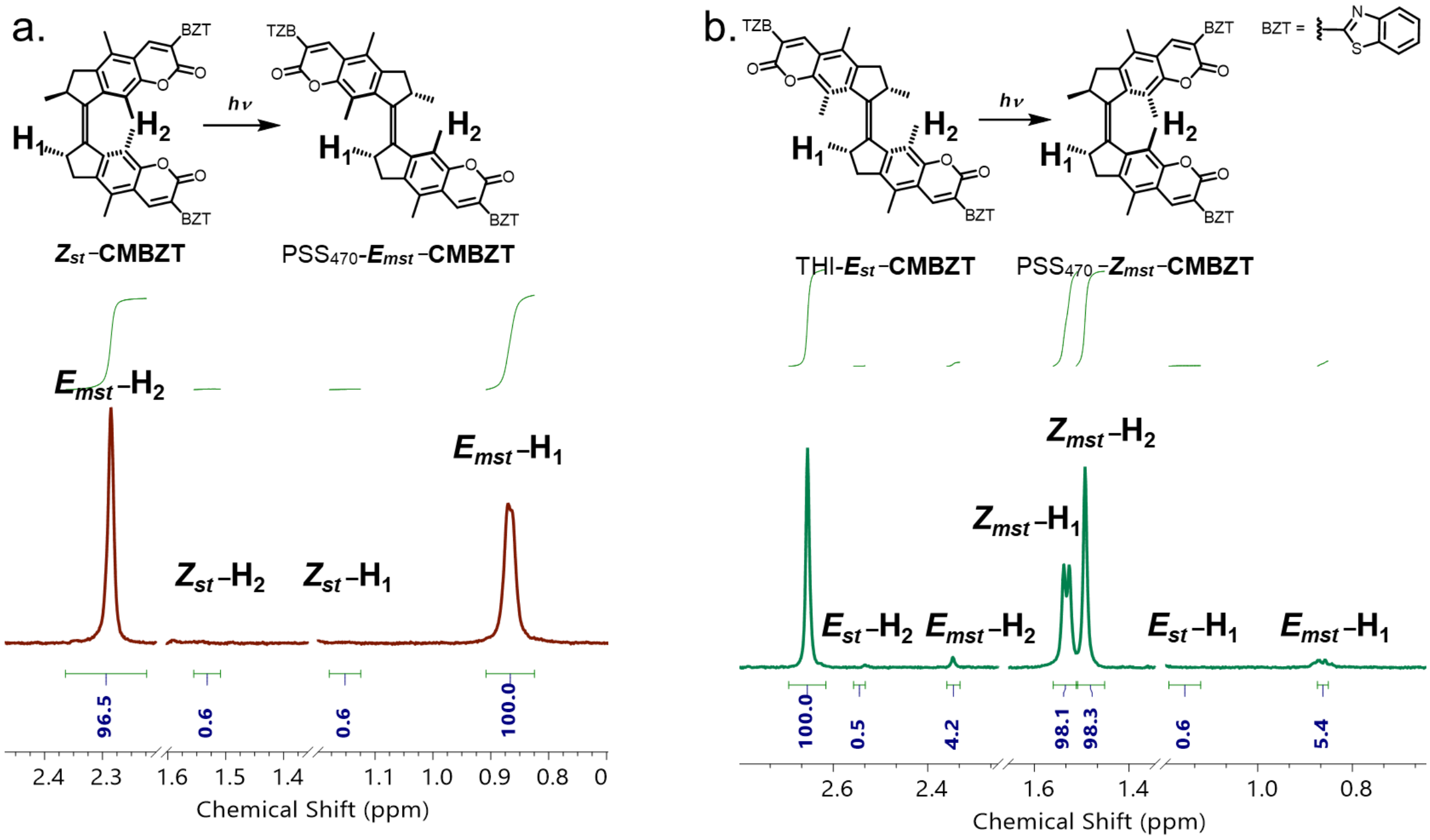

Figure S32. Photo-stationary states (PSS) generation with partially zoomed in ^{1}H NMR (500 MHz, CD_2Cl_2, – 45 °C) of motor **CMBZT** with 470 nm light. a. Generation of PSS$_{470}$-$\boldsymbol{E_{mst}}$-**CMBZT**, with integration of the signal $\mathbf{H_1}$ and $\mathbf{H_2}$, indicating a ratio of 98:2 ($\boldsymbol{E_{mst}}$: $\boldsymbol{Z_{st}}$). b. Generation of PSS$_{470}$-$\boldsymbol{Z_{mst}}$-**CMBZT**, with integration of the signal $\mathbf{H_1}$ and $\mathbf{H_2}$, indicating a ratio of 99:1 ($\boldsymbol{Z_{mst}}$: $\boldsymbol{E_{st}}$).

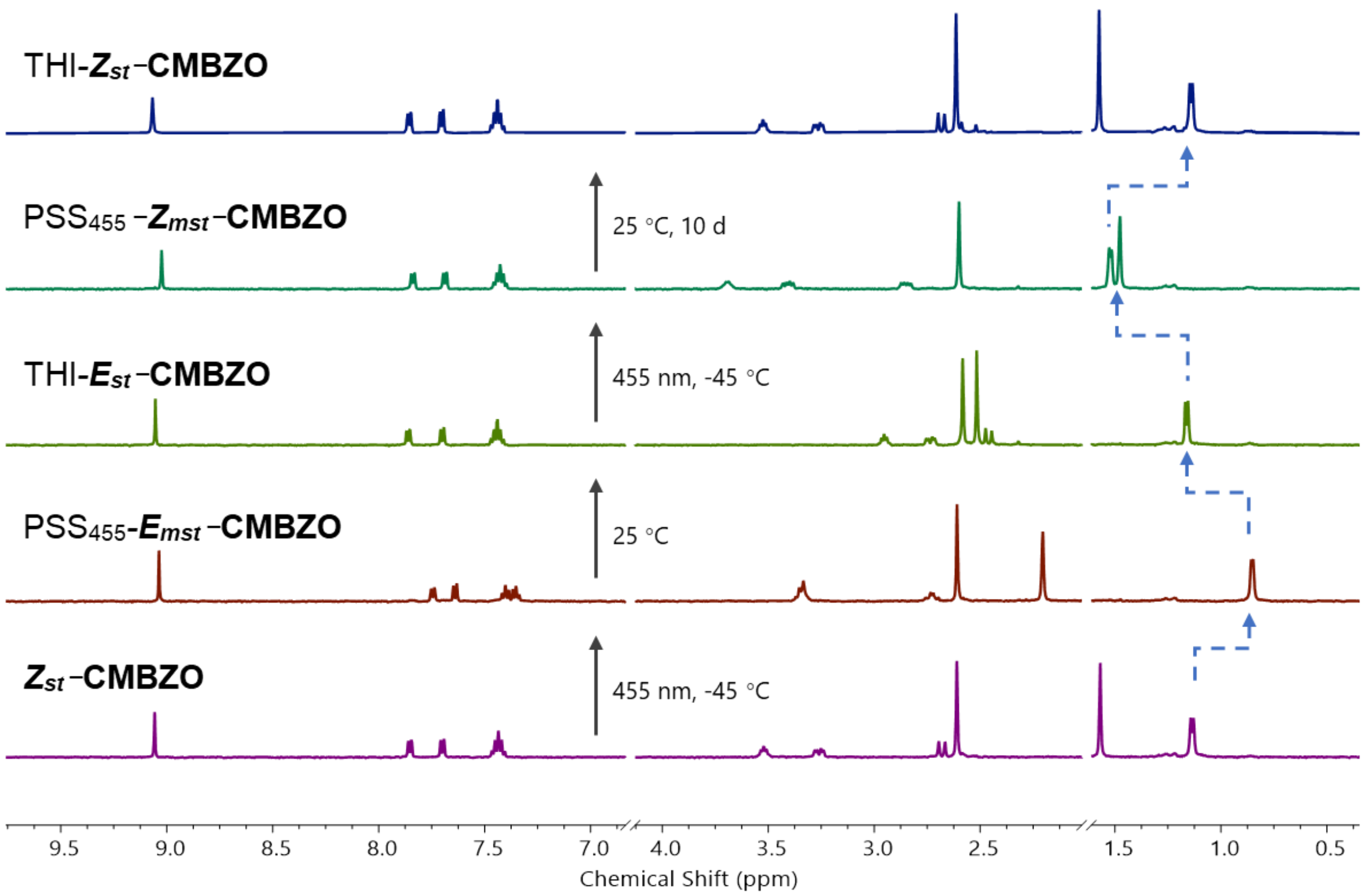

Figure S33. Rotary cycle of motor **CMBZO** followed by ^{1}H NMR (500 MHz, CD_2Cl_2, – 45 °C) starting from ***Z****st*-**CMBZO** (bottom purple line) to PSS455-***E****mst*-**CMBZO** (red line) with 455 nm light irradiation at – 45 °C, followed by heating to room temperature for THI process to generate THI-***E****st*-**CMBZO** (green line); and subsequent irradiation with 455 nm light at – 45 °C to PSS455-***Z****mst*-**CMBZO** (teal line). Finally, the sample was kept at room temperature for 10 d to regenerate ***Z****st*-**CMBZO** (THI-***Z****st*-**CMBZO**, top blue line). All ^{1}H NMR measurements were performed at –45 °C to ensure the chemical shifts were not influenced by temperature. Dashed arrows were used to direct attention to the changes in the chemical shifts (*δ*, ppm) of the characteristic resonances.

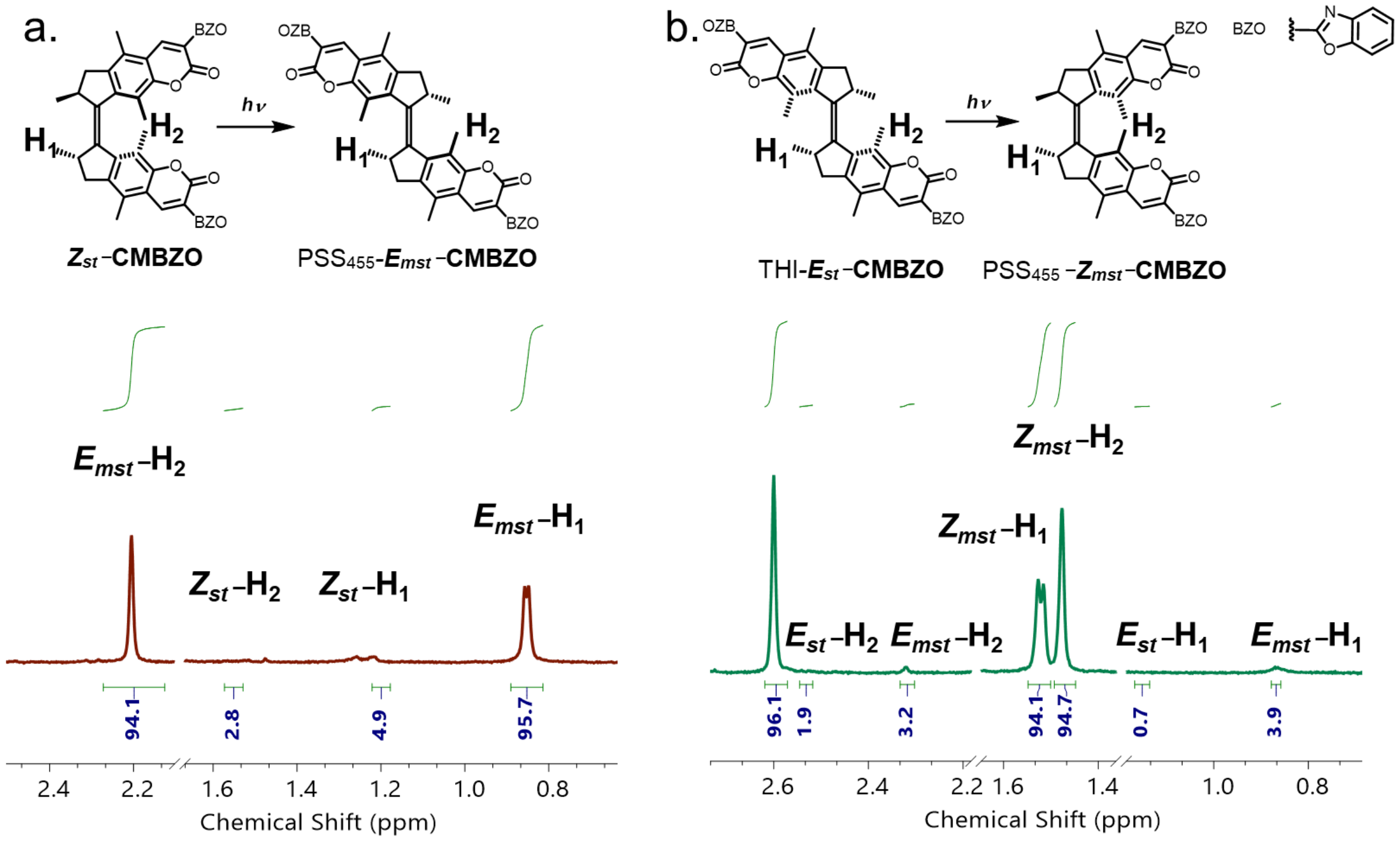

Figure S34. Photo stationary states (PSS) generation with partially zoomed in ^{1}H NMR (500 MHz, CD_2Cl_2, – 45 °C) of motor **CMBZO** with 455 nm light. a. Generation of PSS$_{455}$-***E$_{mst}$***-**CMBZO**, with integration of the signal $\mathbf{H_1}$ and $\mathbf{H_2}$, indicating a ratio of 96:4 (***E$_{mst}$***: ***Z$_{st}$***). b. Generation of PSS$_{455}$-***Z$_{mst}$***-**CMBZO**, with integration of the signal $\mathbf{H_1}$ and $\mathbf{H_2}$, indicating a ratio of 99:1 (***Z$_{mst}$***: ***E$_{st}$***).

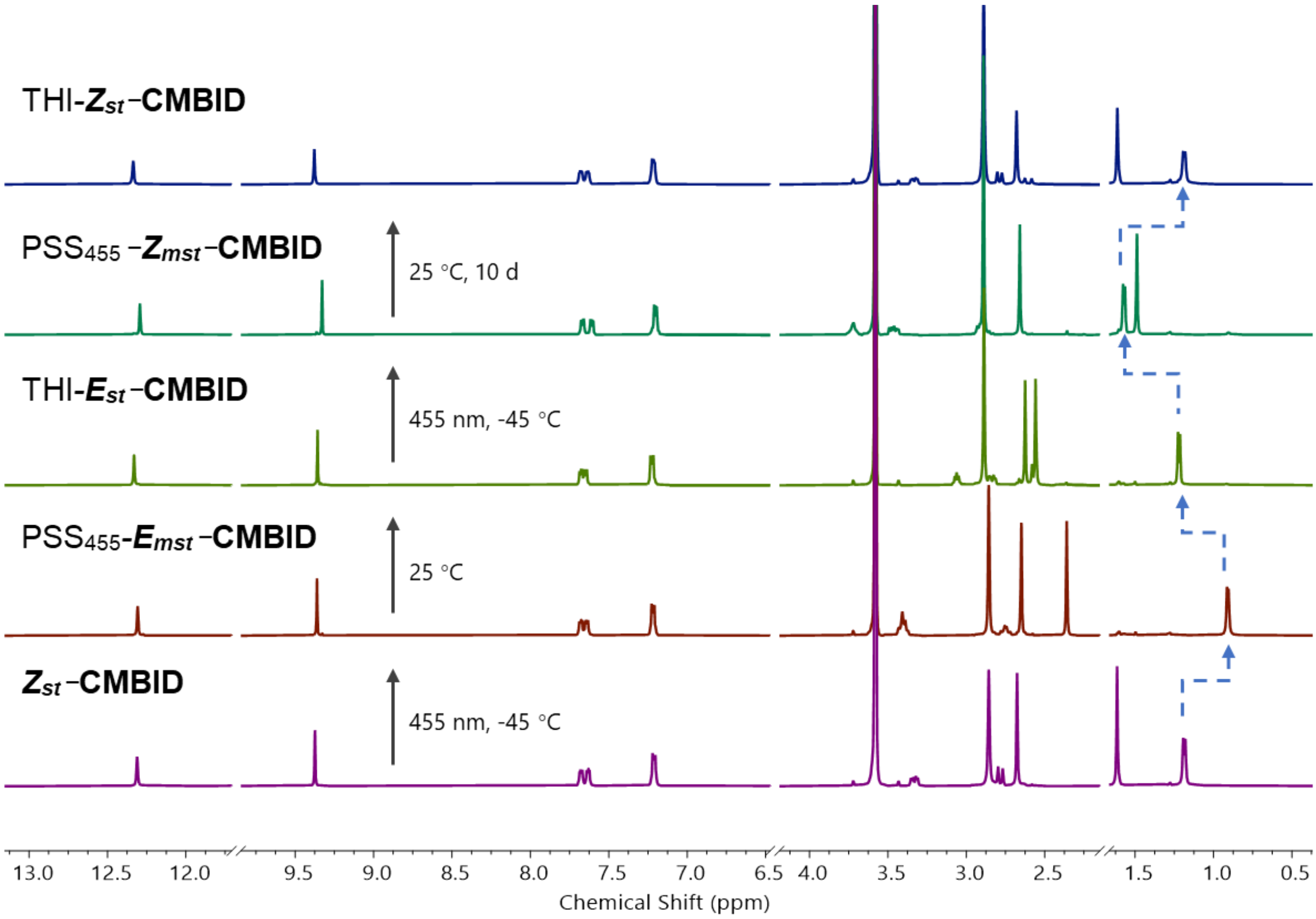

Figure S35. Rotary cycle of motor **CMBID** followed by ^{1}H NMR (500 MHz, THF-d_8, – 45 °C) starting from ***Z**$_{st}$*-**CMBID** (bottom purple line) to PSS_{455}-***E**$_{mst}$*-**CMBID** (red line) with 455 nm light irradiation at – 45 °C, followed by heating to room temperature for THI process to generate THI-***E**$_{st}$*-**CMBID** (green line); and subsequent irradiation with 455 nm light at – 45 °C to PSS_{455}-***Z**$_{mst}$*-**CMBID** (teal line). Finally, the sample was kept at room temperature for 10 d to regenerate ***Z**$_{st}$*-**CMBID** (THI-***Z**$_{st}$*-**CMBID** top blue line). All ^{1}H NMR measurements were performed at – 45 °C to ensure the chemical shifts were not influenced by temperature. Dashed arrows were used to direct attention to the changes in the chemical shifts (δ, ppm) of the characteristic resonances.

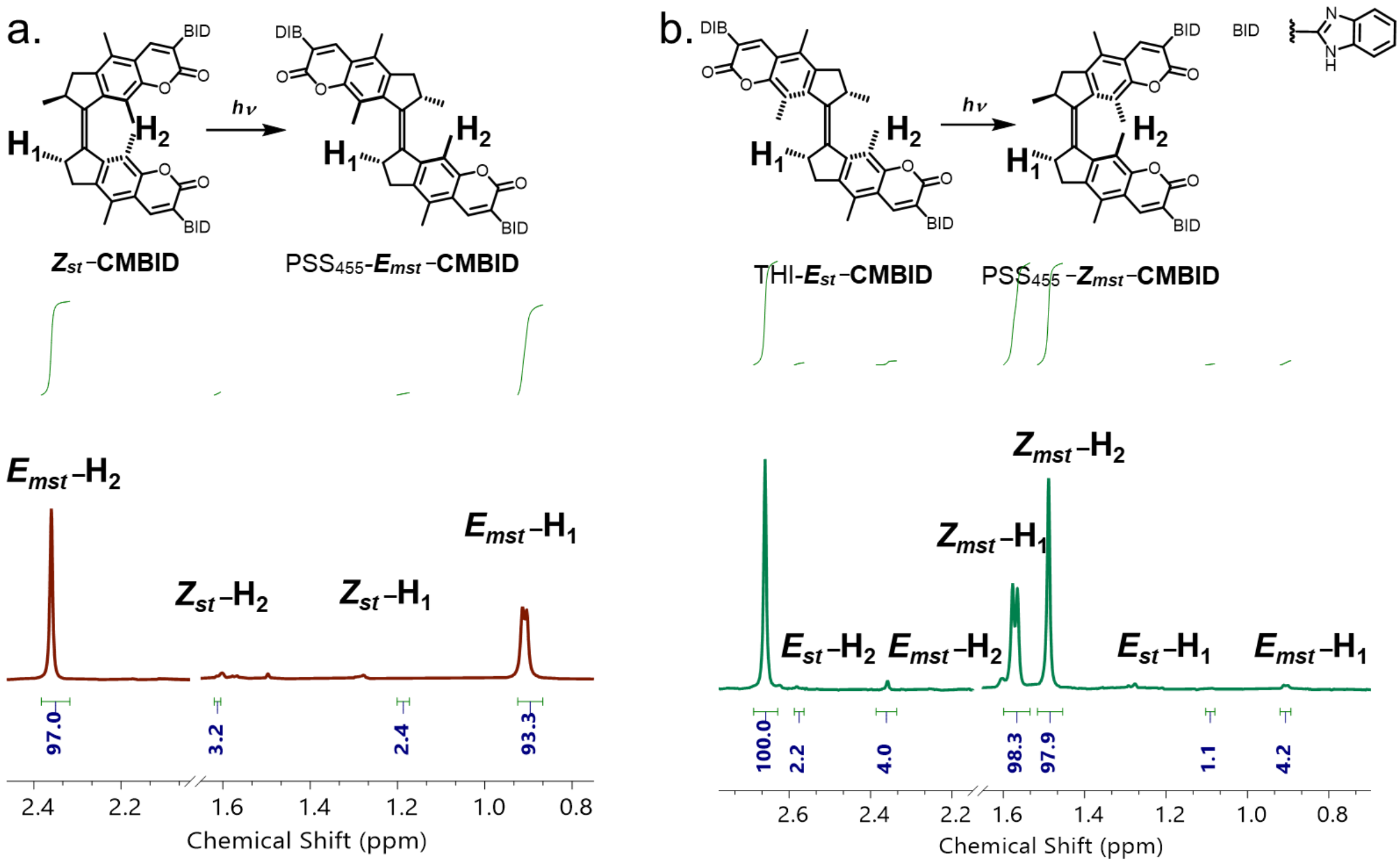

Figure S36. Photo stationary states (PSS) generation with partially zoomed in ^{1}H NMR (500 MHz, THF-d_8, – 45 °C) of motor **CMBID** with 455 nm light. a. Generation of PSS$_{455}$-***E**$_{mst}$*-**CMBID**, with integration of the signal $\mathbf{H_1}$ and $\mathbf{H_2}$, indicating a ratio of 97:3 (***E**$_{mst}$*: ***Z**$_{st}$*). b. Generation of PSS$_{455}$-***Z**$_{mst}$*-**CMBID**, with integration of the signal $\mathbf{H_1}$ and $\mathbf{H_2}$, indicating a ratio of 98:2 (***Z**$_{mst}$*: ***E**$_{st}$*).

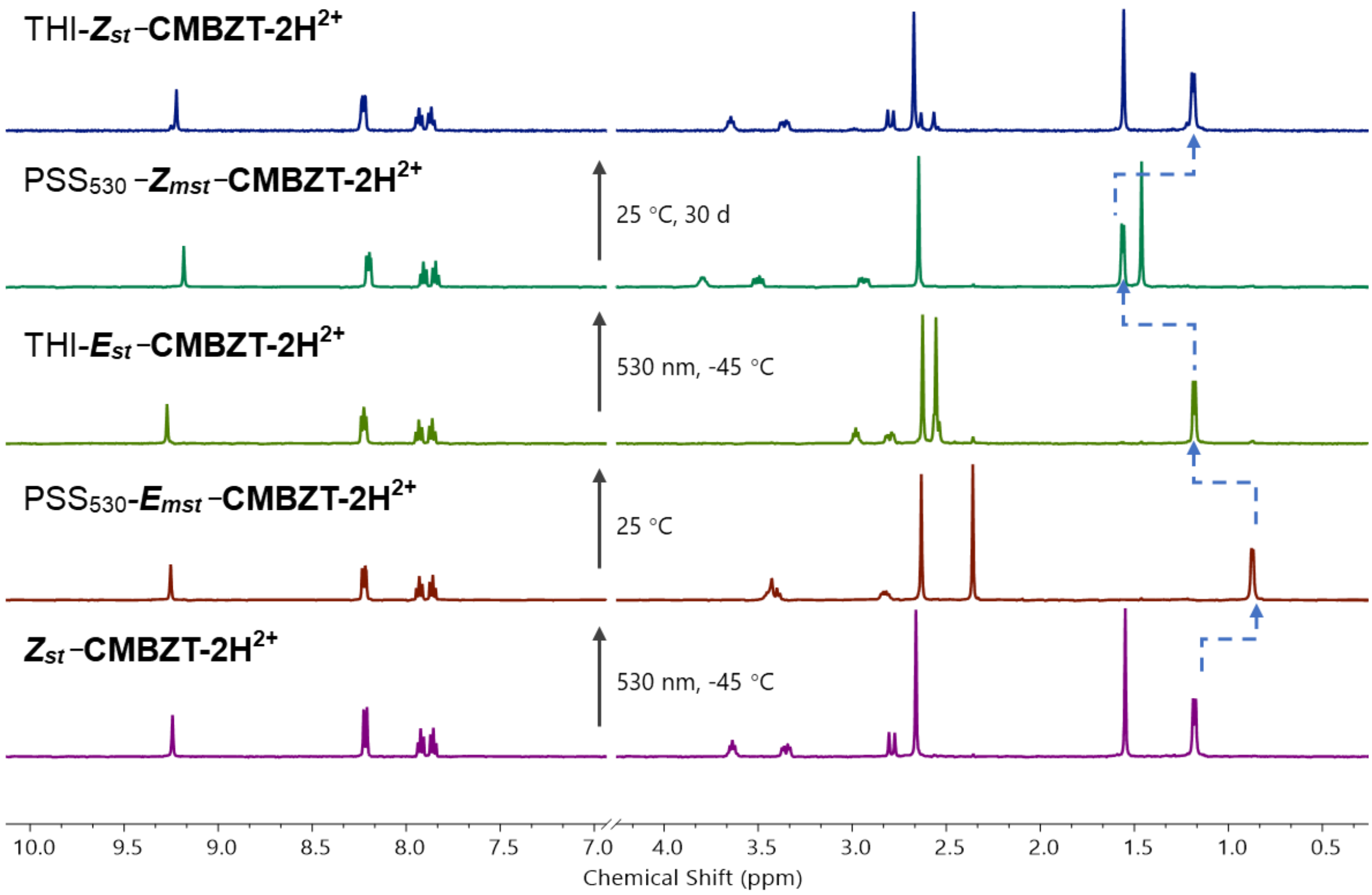

Figure S37. Rotary cycle of motor **CMBZT-2H^{2+}** followed by ^{1}H NMR (500 MHz, 2mM in CD_2Cl_2 + 100 eq. of TFA-d_1) starting from ***Z**$_{st}$***-CMBZT-2H^{2+}** (bottom purple line) to PSS$_{530}$-***E**$_{mst}$***-CMBZT-2H^{2+}** (red line) with 530 nm light irradiation at – 45 °C, followed by heating to room temperature for THI process to generate THI-***E**$_{st}$***-CMBZT-2H^{2+}** (green line); and subsequent irradiation with 530 nm light at – 45 °C to PSS$_{530}$-***Z**$_{mst}$***-CMBZT-2H^{2+}** (teal line). Finally, the sample was kept at room temperature for 30 d to regenerate ***Z**$_{st}$***-CMBZT-2H^{2+}** (THI-***Z**$_{st}$***-CMBZT-2H^{2+}** top blue line). All ^{1}H NMR measurements were performed at – 45 °C to ensure the chemical shifts were not influenced by temperature. Dashed arrows were used to direct attention to the changes in the chemical shifts (δ, ppm) of the characteristic resonances.

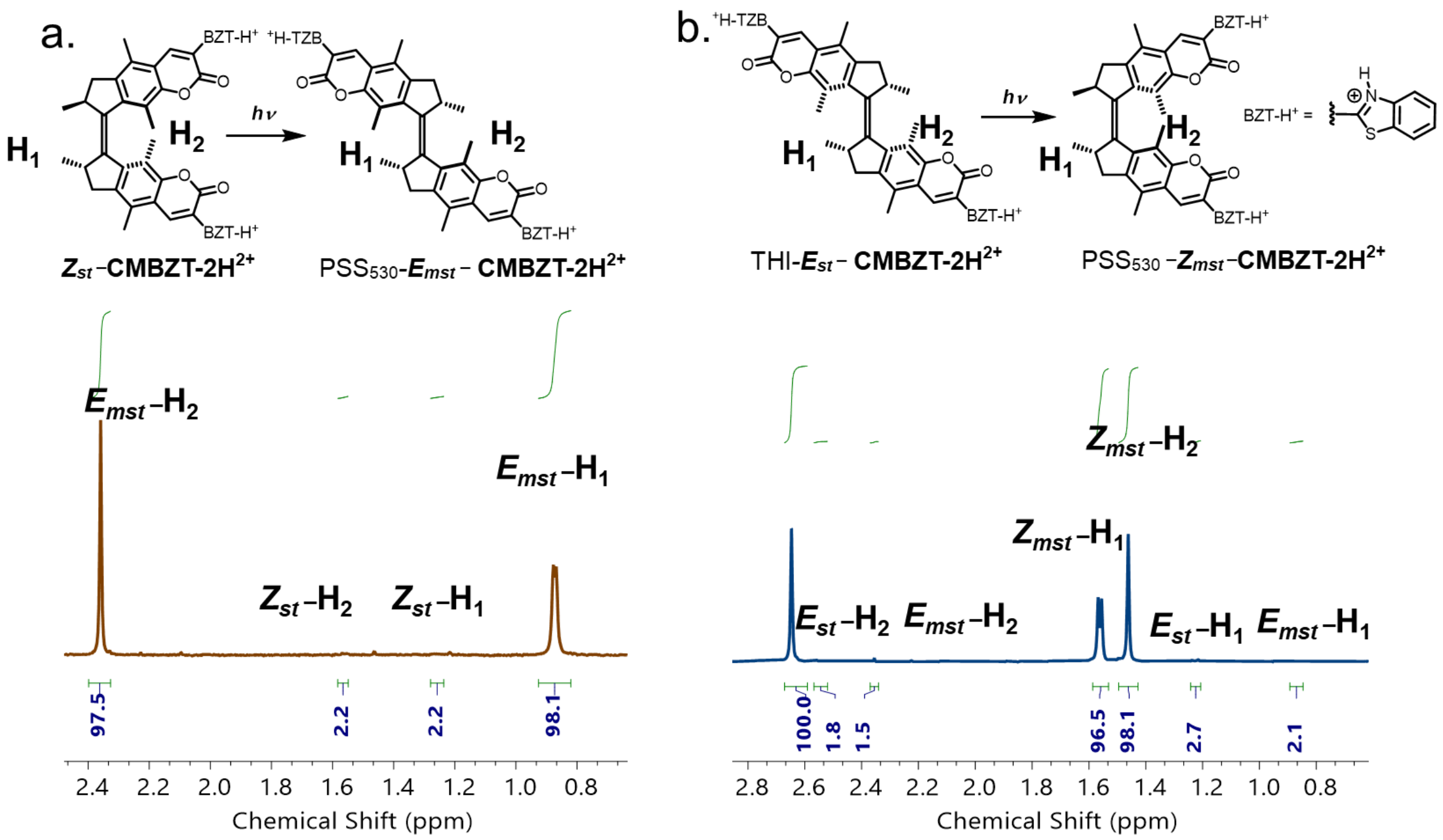

Figure S38. Photo stationary states (PSS) generation with partially zoomed in ^{1}H NMR (500 MHz, 2mM in CD_2Cl_2 + 100 eq. of TFA-d_1) of motor **CMBZT-2H^{2+}** with 530 nm light. a. Generation of PSS$_{530}$-***E**$_{mst}$*-**CMBZT-2H^{2+}**, with integration of the signal **H$_1$** and **H$_2$**, indicating a ratio of 98:2 (***E**$_{mst}$*: ***Z**$_{st}$*). b. Generation of PSS$_{530}$-***Z**$_{mst}$*-**CMBZT-2H^{2+}**, with integration of the signal **H$_1$** and **H$_2$**, indicating a ratio of 98:2 (***Z**$_{mst}$*: ***E**$_{st}$*).

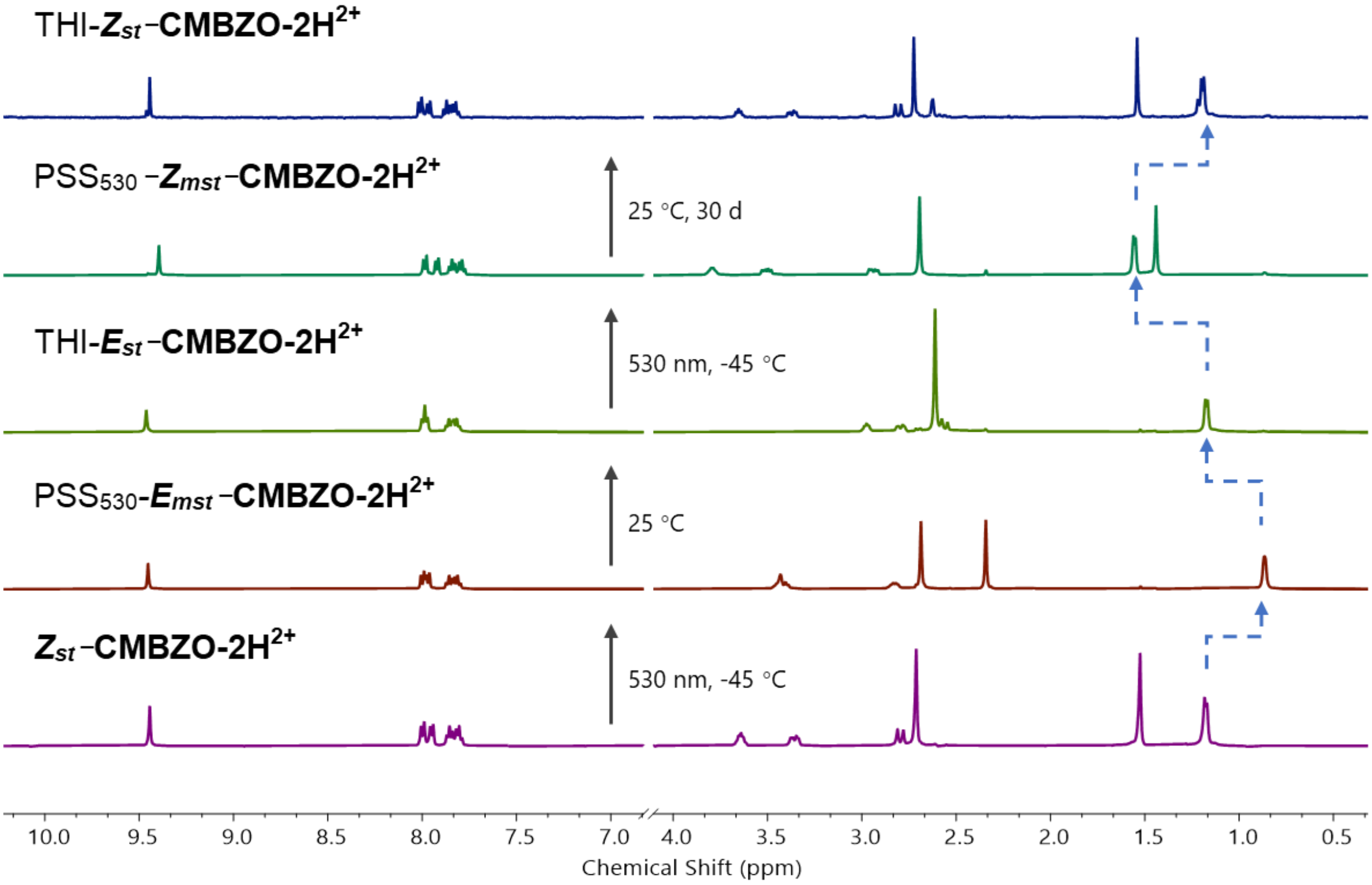

Figure S39. Rotary cycle of motor **CMBZO-2H**$^{2+}$ followed by ^{1}H NMR (500 MHz, 2mM in CD_2Cl_2 + 100 eq. of TFA-d_1) starting from ***Z**$_{st}$***-CMBZT-2H**$^{2+}$ (bottom purple line) to PSS$_{530}$-***E**$_{mst}$***-CMBZO-2H**$^{2+}$ (red line) with 530 nm light irradiation at – 45 °C, followed by heating to room temperature for THI process to generate THI-***E**$_{st}$***-CMBZO-2H**$^{2+}$ (green line); and subsequent irradiation with 530 nm light at – 45 °C to PSS$_{530}$-***Z**$_{mst}$***-CMBZO-2H**$^{2+}$ (teal line). Finally, the sample was kept at room temperature for 30 d to regenerate ***Z**$_{st}$***-CMBZO-2H**$^{2+}$ (THI-***Z**$_{st}$***-CMBZO-2H**$^{2+}$ top blue line). All ^{1}H NMR measurements were performed at – 45 °C to ensure the chemical shifts were not influenced by temperature. Dashed arrows were used to direct attention to the changes in the chemical shifts (δ, ppm) of the characteristic resonances.

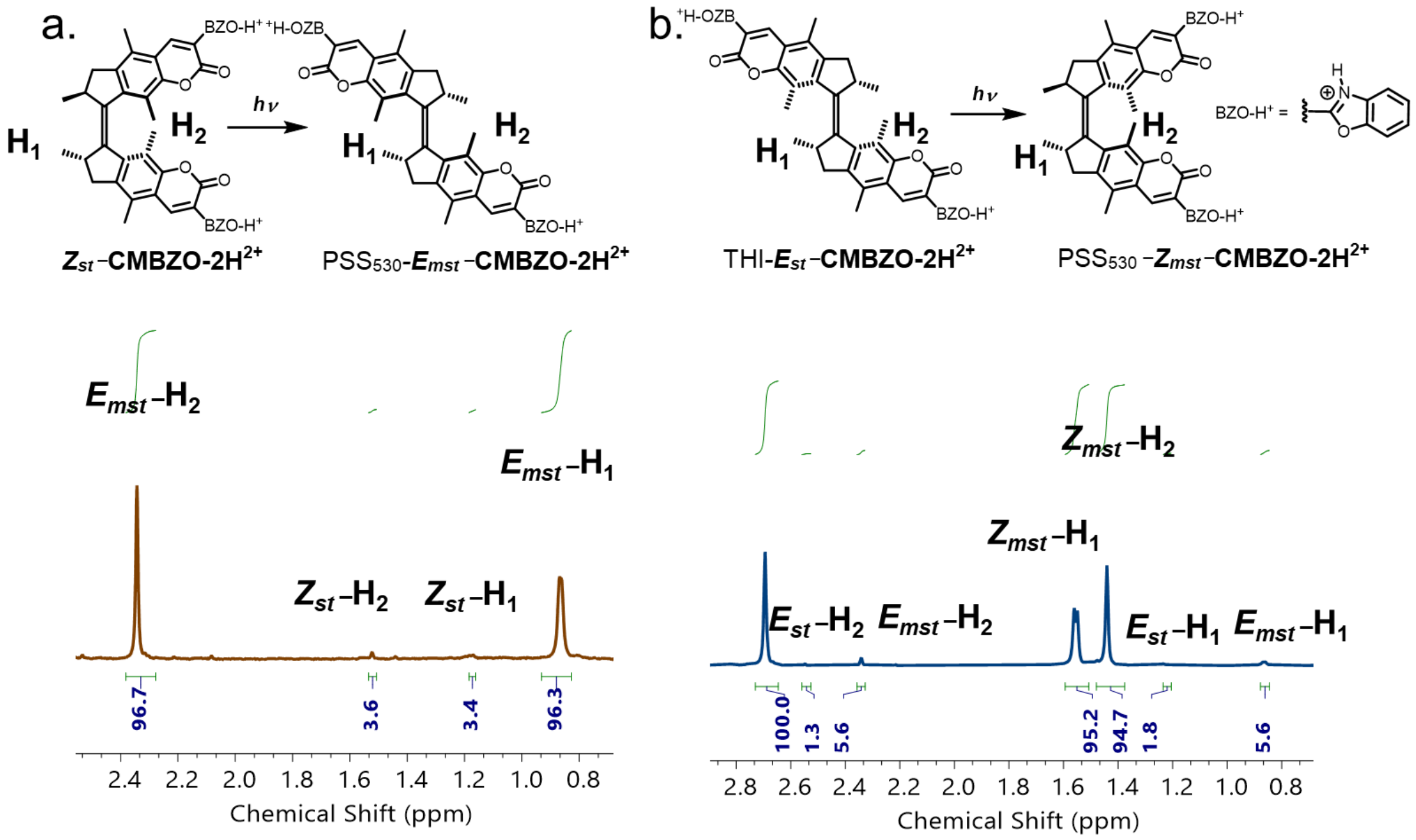

Figure S40. Photo stationary states (PSS) generation with partially zoomed in ^{1}H NMR (500 MHz, 2mM in CD_2Cl_2 + 100 eq. of TFA-d_1) of motor **CMBZO-2H^{2+}** with 530 nm light. a. Generation of PSS$_{530}$-$\boldsymbol{E_{mst}}$-**CMBZO-2H^{2+}**, with integration of the signal **H$_1$** and **H$_2$**, indicating a ratio of 97:3 ($\boldsymbol{E_{mst}}$: $\boldsymbol{Z_{st}}$). b. Generation of PSS$_{530}$-$\boldsymbol{Z_{mst}}$-**CMBZO-2H^{2+}**, with integration of the signal **H$_1$** and **H$_2$**, indicating a ratio of 98:2 ($\boldsymbol{Z_{mst}}$: $\boldsymbol{E_{st}}$).

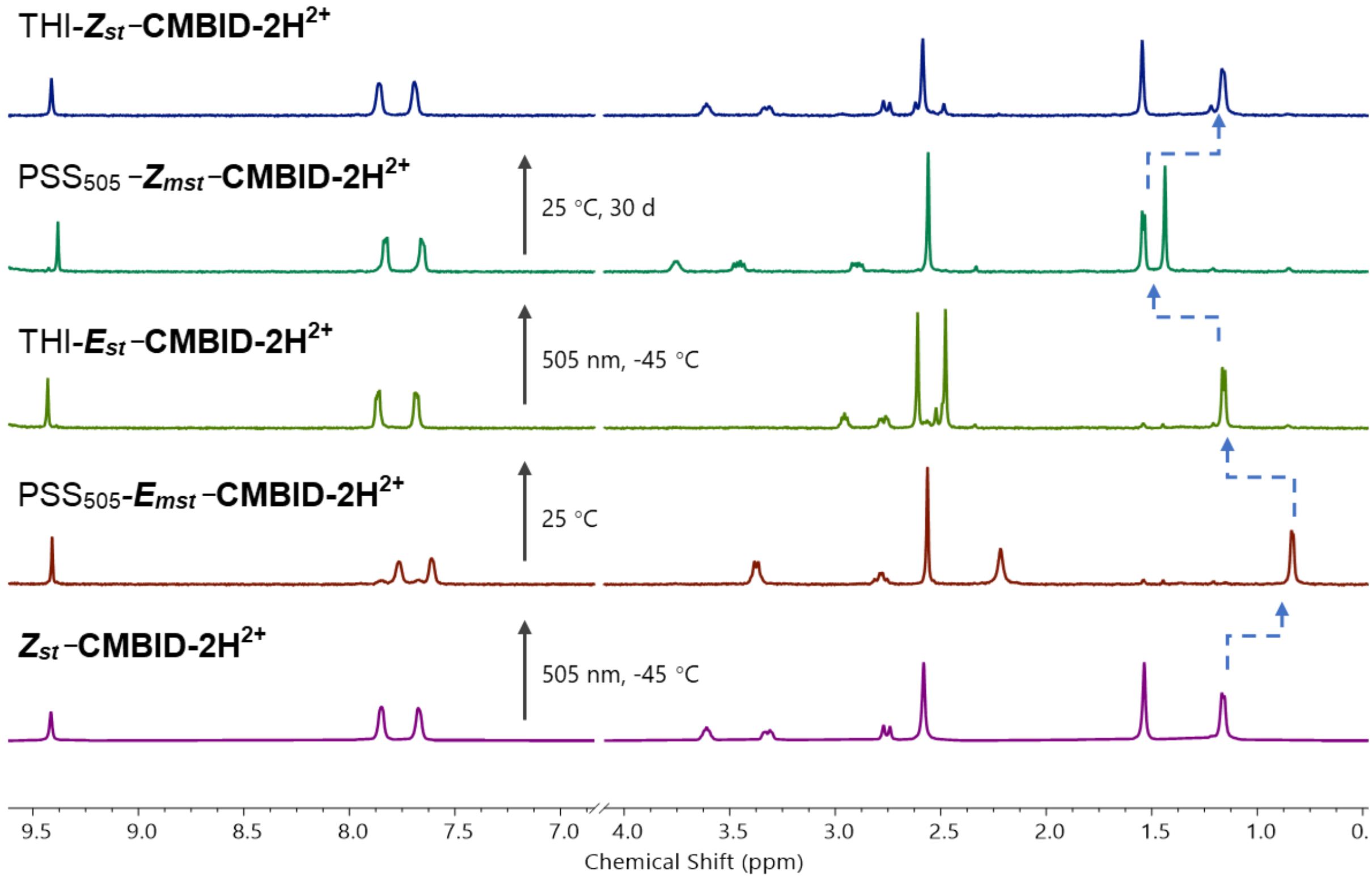

Figure S41. Rotary cycle of motor **CMBID-2H^{2+}** followed by ^{1}H NMR (500 MHz, 2mM in CD_2Cl_2 + 20 eq. of TFA-d_1) starting from ***Z$_{st}$*-CMBID-2H^{2+}** (bottom purple line) to PSS$_{505}$-***E$_{mst}$*-CMBID-2H^{2+}** (red line) with 505nm light irradiation at – 45 °C, followed by heating to room temperature for THI process to generate THI-***E$_{st}$*-CMBID-2H^{2+}** (green line); and subsequent irradiation with 505 nm light at – 45 °C to PSS$_{505}$-***Z$_{mst}$*-CMBID-2H^{2+}** (teal line). Finally, the sample was kept at room temperature for 30 d to regenerate ***Z$_{st}$*-CMBID-2H^{2+}** (THI-***Z$_{st}$*-CMBID-2H^{2+}** top blue line). All ^{1}H NMR measurements were performed at – 45 °C to ensure the chemical shifts were not influenced by temperature. Arrows were used to direct attention to the changes in the chemical shifts (δ, ppm) of the characteristic resonances.

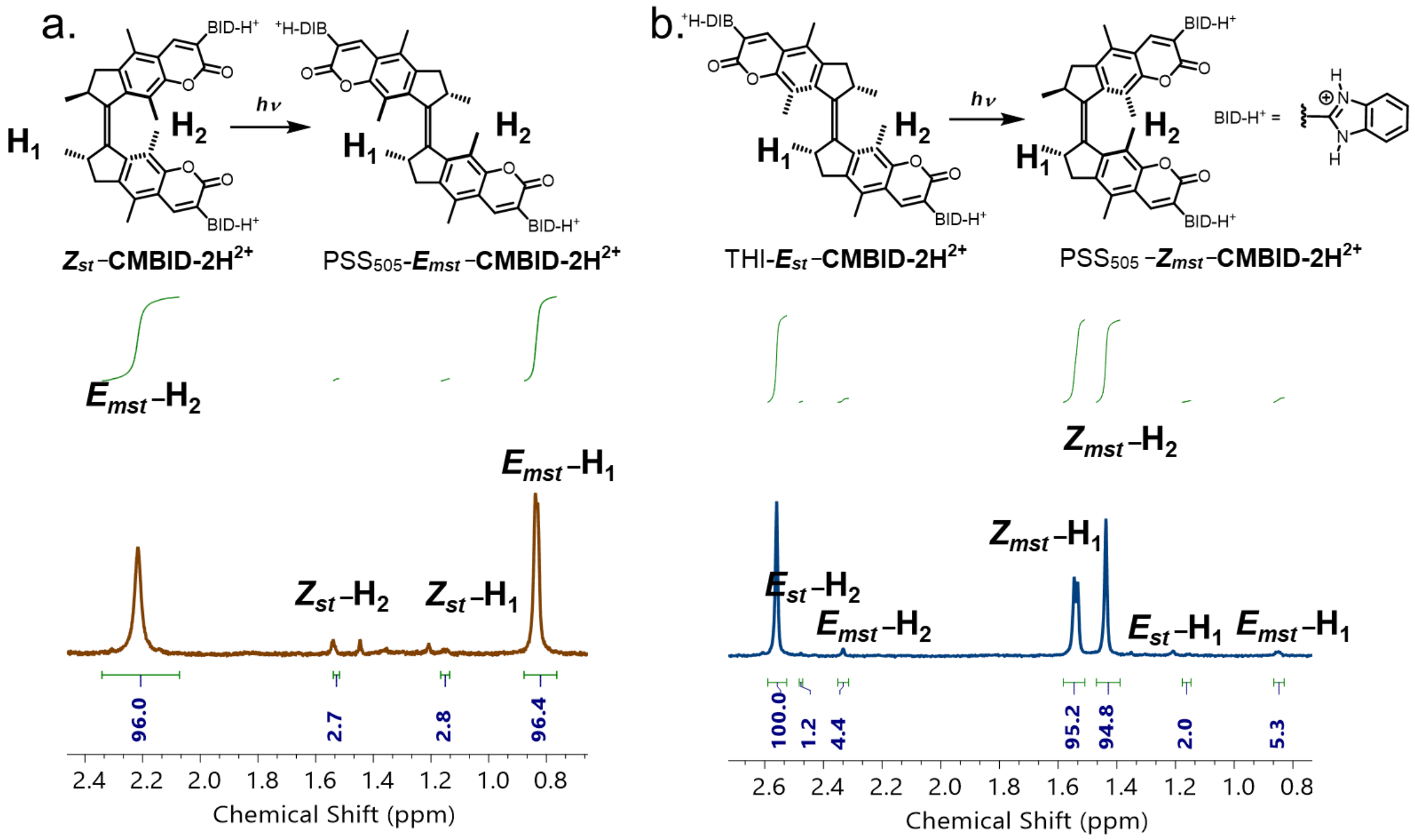

Figure S42. Photo stationary states (PSS) generation with partially zoomed in ^{1}H NMR (500 MHz, 2mM in CD_2Cl_2 + 20 eq. of TFA-d_1) of motor **CMBID-2H^{2+}** with 505 nm light. a. Generation of PSS$_{505}$-***E$_{mst}$***-**CMBID-2H^{2+}**, with integration of the signal **H$_1$** and **H$_2$**, indicating a ratio of 97:3 (***E$_{mst}$***: ***Z$_{st}$***). b. Generation of PSS$_{505}$-***Z$_{mst}$***-**CMBID-2H^{2+}**, with integration of the signal **H$_1$** and **H$_2$**, indicating a ratio of 98:2 (***Z$_{mst}$***: ***E$_{st}$***).

10. Rotation quantum yield determination

For the unprotonated states of the **CMR**, the absorption maxima of the relevant bands are located in the 455 – 470 nm range. Therefore, the photon flux of the LED source was determined using a relative method, with ferrioxalate chemical actinometry employed as the reference. Experimental details are provided below.

Ferrioxalate Chemical Actinometry

A modification of a standard protocol was applied for the determination of the photon flux.[9-10] An aqueous H_2SO_4 solution (0.05 M) containing freshly recrystallized $K_3[Fe(C_2O_4)_3]$ (41 mM, 2 mL, 1 cm quartz cuvette) was irradiated at 20 °C for a given period in the dark with a 365 nm LED. The solution was then diluted with 1.0 mL of an aqueous H_2SO_4 solution (0.5 M) containing phenanthroline (1 g/L) and NaOAc (122.5 g/L) and left to react for 10 min. The absorption at λ= 510 nm was measured and compared to an identically prepared non-irradiated sample. The concentration of $[Fe(phenanthroline)_3]^{2+}$ complex was calculated using its molar absorptivity (ε = 11100 M^{-1} cm^{-1}) and considering the dilution. The quantity of Fe^{2+} ions expressed in mol was plotted versus time (expressed in seconds) and the slope, obtained by linear fitting the data points to the equation y = ax +b, equals the rate of formation of the Fe^{2+} ion at the given wavelength. This rate can be converted into the photon flux (I) by dividing it by the quantum yield of $[Fe(phenanthroline)_3]^{2+}$ complex at the wavelength of interest (Φ^{455nm} = 1.02, Φ^{470nm} = 0.96) and by the probability of photon absorption of the Fe^{3+} complex (approximated to 1 as we were working in the total absorption regime). The obtained molar photon fluxes were $I^{455nm} = 6.9247 \cdot 10^{-5}$ mmol s^{-1}, $I^{470nm} = 5.3756 \cdot 10^{-5}$ mmol s^{-1}. These values are calculated assuming a single wavelength light source. Due to the possible wavelength dependency of the quantum yields, this point represents a source of uncertainty.

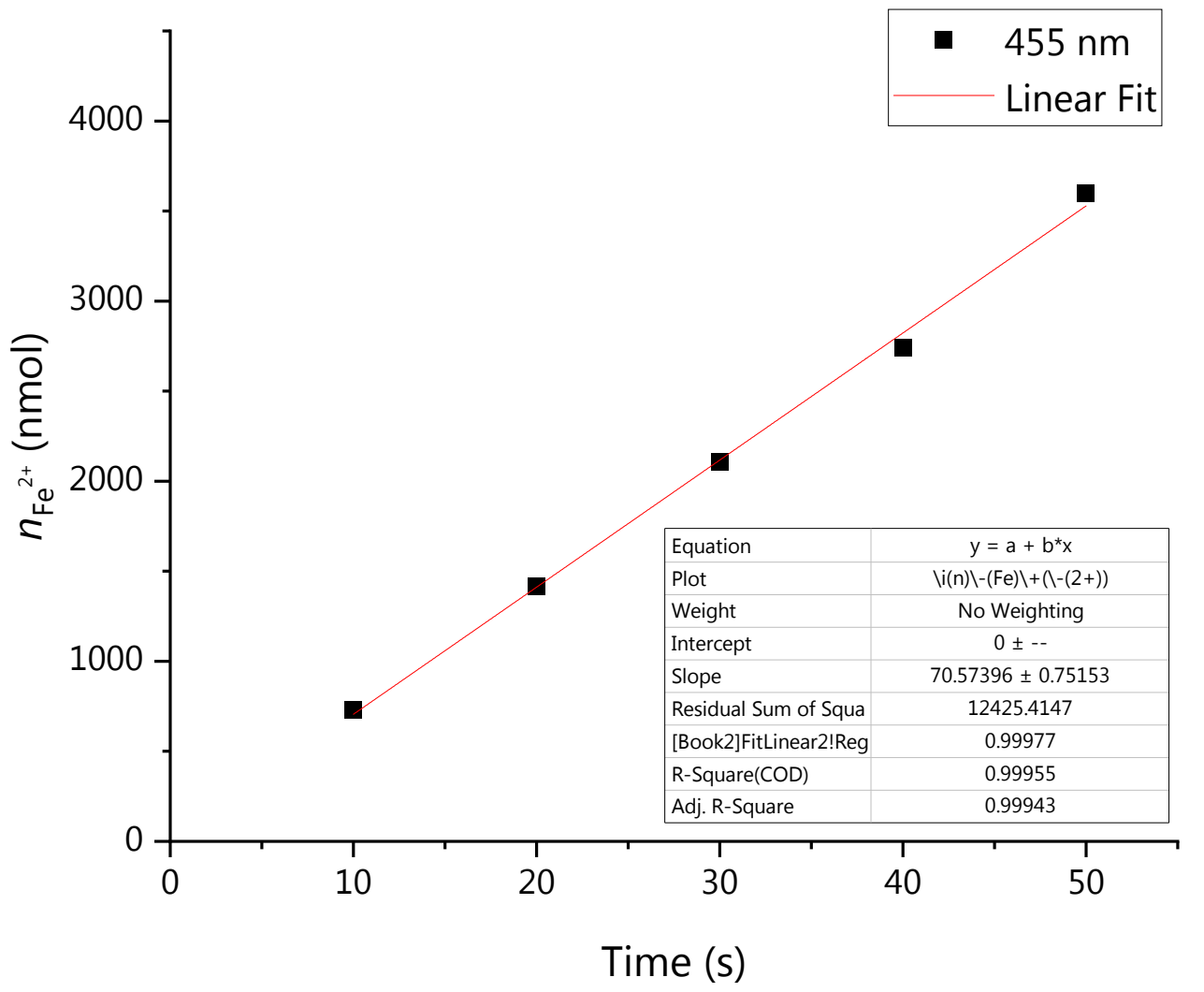

Figure S43. Linear fitting of the Fe^{2+} moles generated upon irradiation of the $[Fe(C_2O_4)_3]^{2+}$ complex 455 nm at different irradiation times.

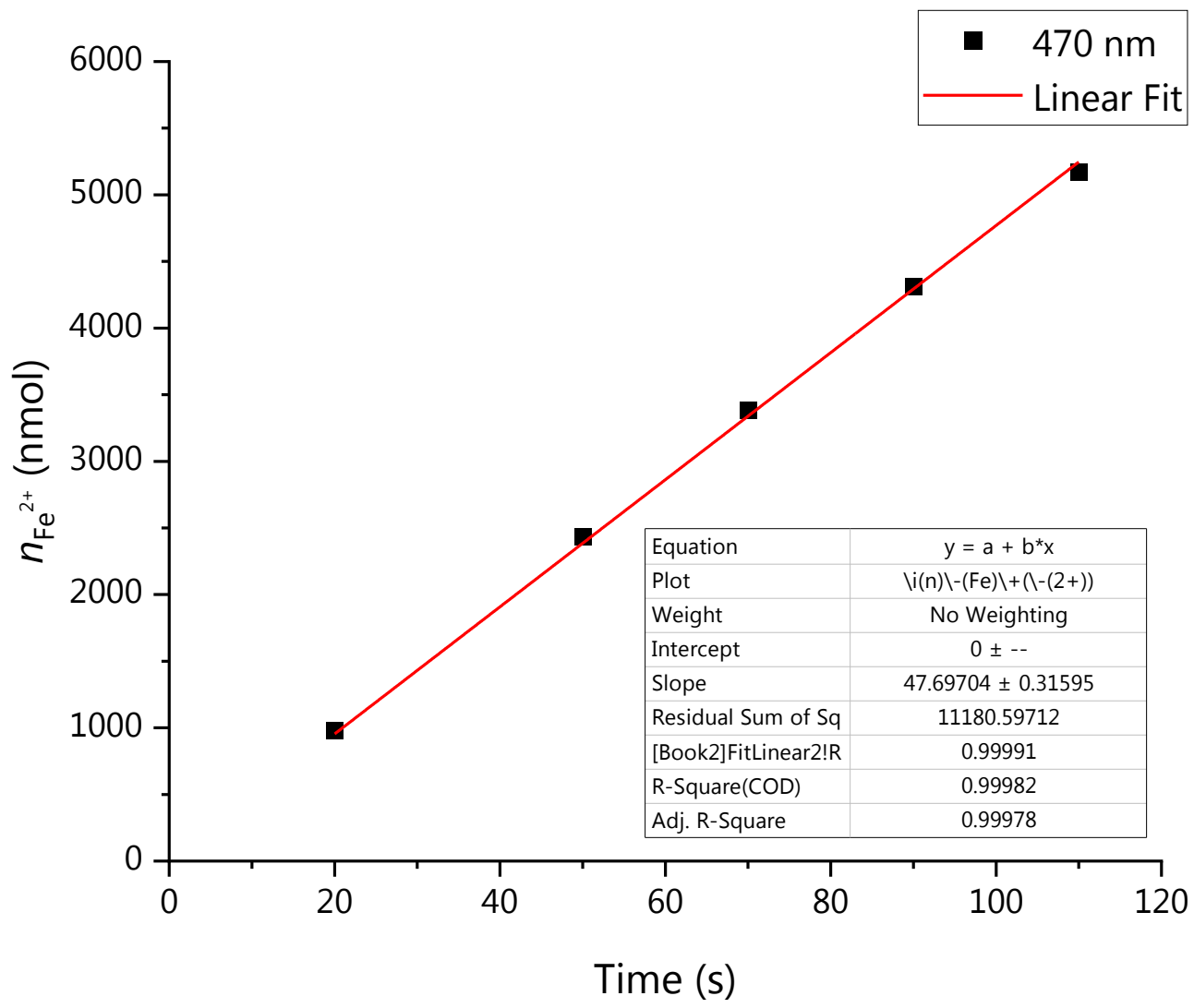

Figure S44. Linear fitting of the Fe^{2+} moles generated upon irradiation of the $[Fe(C_2O_4)_3]^{2+}$ complex 470 nm at different irradiation times.

For the protonated states **CMR-2H^{2+}**, however, there is little commonly reported chemical actinometer available in the 500–600 nm range. Consequently, the photon flux of the LED was determined by direct measurement using a power meter, with the LED treated as a monochromatic light source.[11] Additionally, to account for proton loss within the cuvettes and solvent, power meter readings were recorded both before and after insertion of the cuvettes containing solvent, and the average value was taken as the effective proton flux reaching the sample solution.

The photo fluxes were calculated according to the meter readings based on eq. 1:

$$\Phi^{\cdot}{}_{\mathrm{PH}} = \frac{P \cdot A \cdot \lambda}{h \cdot c \cdot N_A} \tag{1}$$

where $\Phi^{\cdot}{}_{\mathrm{PH}}$ (mol/s) is the photon flux, P (w/cm^{-2}) is the readings of the meters, A (cm^2) is the illuminated area, λ (nm) is the wavelength of the LED, h (J/s) is the Planck constant, c (m/s) is the speed of light, N_A (mol^{-1}) is the Avogadro constant.

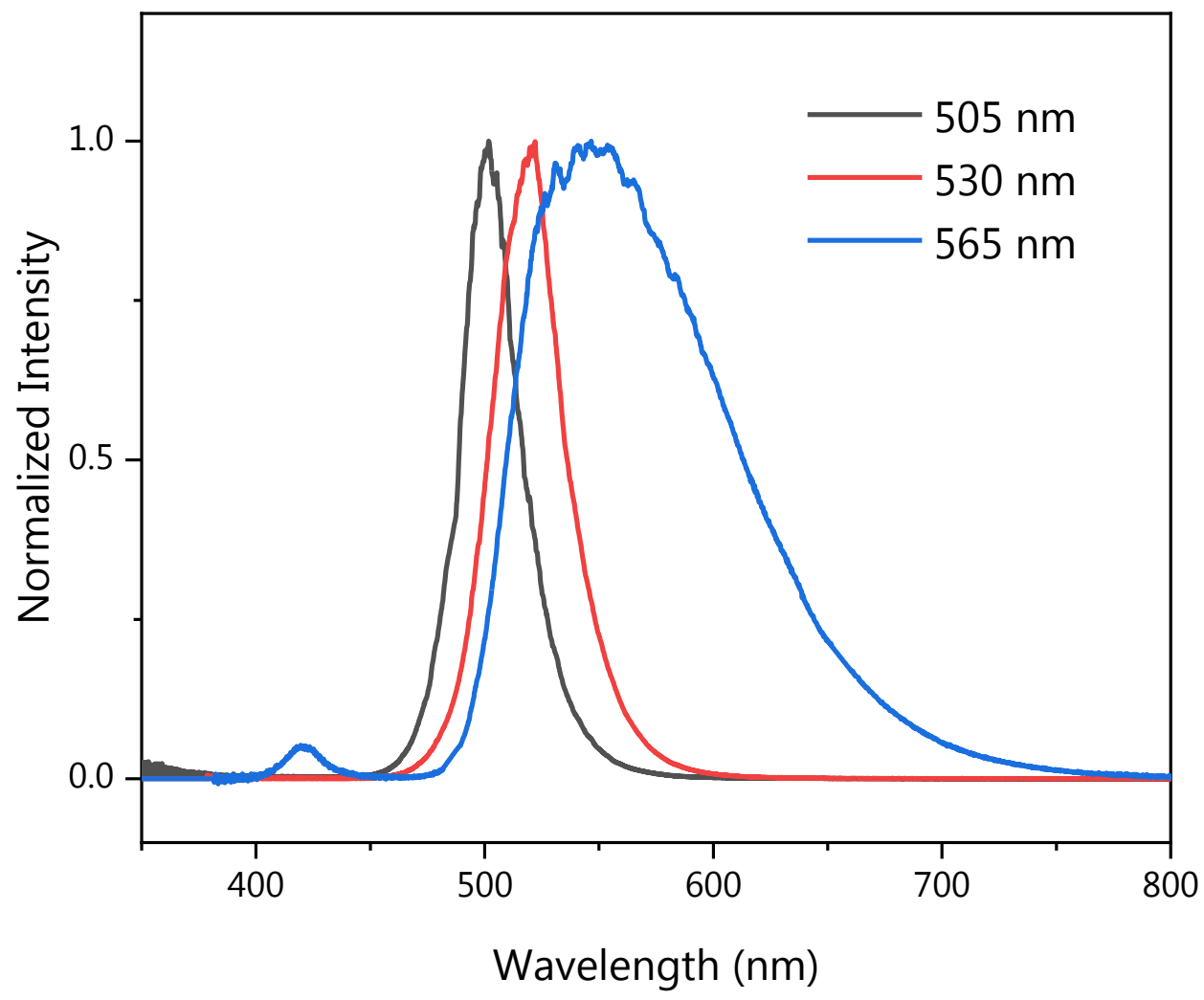

Figure S45. Emission spectra of the corresponding LED labeled as 505, 530 and 565 nm.

Table S2. Photon flux measurements of different LEDs with **CMR-2H^{2+}** motors.

Compound	Repeat	Power without cuvettes (mW)	Power with cuvettes (mW)	Power at sample (mW)	Photon Flux (mE/s)
Z_{st}-CMBZT (555 nm)	1	0.566	0.455	0.51	2.37 10^{-6}
	2	0.574	0.462	0.518	2.41 10^{-6}
	3	0.573	0.460	0.516	2.40 10^{-6}
Z_{st}-CMBZT (523 nm)	1	0.185	0.149	0.167	7.30 10^{-7}
	2	0.179	0.142	0.161	7.04 10^{-7}
	3	0.180	0.140	0.160	7.00 10^{-7}
Z_{st}-CMBZO (555 nm)	1	0.571	0.458	0.515	2.39 10^{-6}
	2	0.574	0.446	0.518	2.40 10^{-6}
	3	0.57	0.438	0.516	2.39 10^{-6}
E_{st}-CMBZO (523 nm)	1	0.191	0.154	0.172	7.52 10^{-7}
	2	0.181	0.144	0.162	7.08 10^{-7}
	3	0.181	0.143	0.162	7.08 10^{-7}
Z_{st}-CMBID/ E_{st}-CMBID (505 nm)	1	1.060	0.826	0.943	3.95 10^{-6}
	2	1.090	0.871	0.981	4.11 10^{-6}
	3	1.093	0.867	0.980	4.10 10^{-6}

Quantum Yield by UV-Vis

Solutions of the stable state of a specific motor (S) in dichloromethane (10–20 μM) were irradiated with an LED of a selected wavelength (i.e. 455, 470, 505, 530, 565 nm) to lead to the formation of the metastable state (M). The solutions were kept at -15 °C during the irradiation to avoid the thermal helix inversion from the metastable state to the next stable state. The spectra were collected following the evolution of the absorption at the wavelength of irradiation; the average absorbance value recorded between 600-800 nm was subtracted to the datapoints. The data was subsequently fitted using COPASI 4.29[12] following the same approach developed by Stranius & Börjesson.6 Equiv. 14 in the original article

$$\frac{d[S]}{dt} = -\frac{QY_{SM} \cdot I \cdot \beta_S(t)}{N_A \cdot V} + \frac{QY_{MS} \cdot I \cdot \beta_M(t)}{N_A \cdot V}$$

was used to determine both approximated QYs (QY_{SM} for the formation of the metastable state form the stable and QY_{MS} for the opposite photochemical reaction). I is the photon flux, previously determined with ferrioxalate actinometry, N_A the Avogadro number, V the total volume of the irradiated solution (2 mL) and β the fractions of photons absorbed by either the stable or the metastable state. The decay was fitted by the ODE solver present in COPASI, using a Levenberg-Marquardt algorithm with randomized initial conditions. To obtain physically sound results, the boundaries for the QYs values were fixed between 1×10^{-6} and 1. The measurements were triplicated and averaged to afford the values presented in **Table S3**.

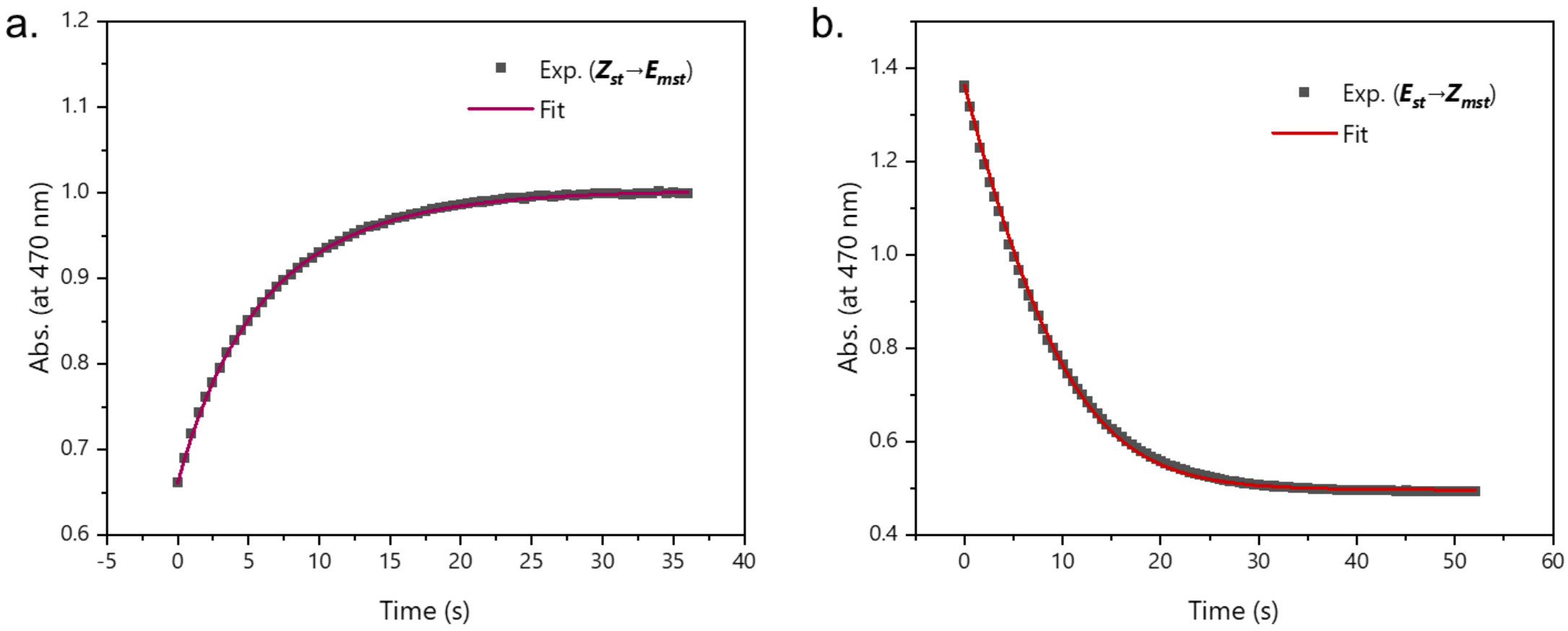

Figure S46. Evolution of the absorption at 470 nm during the irradiation of (a). ***Z**$_{st}$*-**CMBZT** and (b). ***E**$_{st}$*-**CMBZT** in THF with 470 nm light. The red line represents the fit obtained with the ODE solver from COPASI.

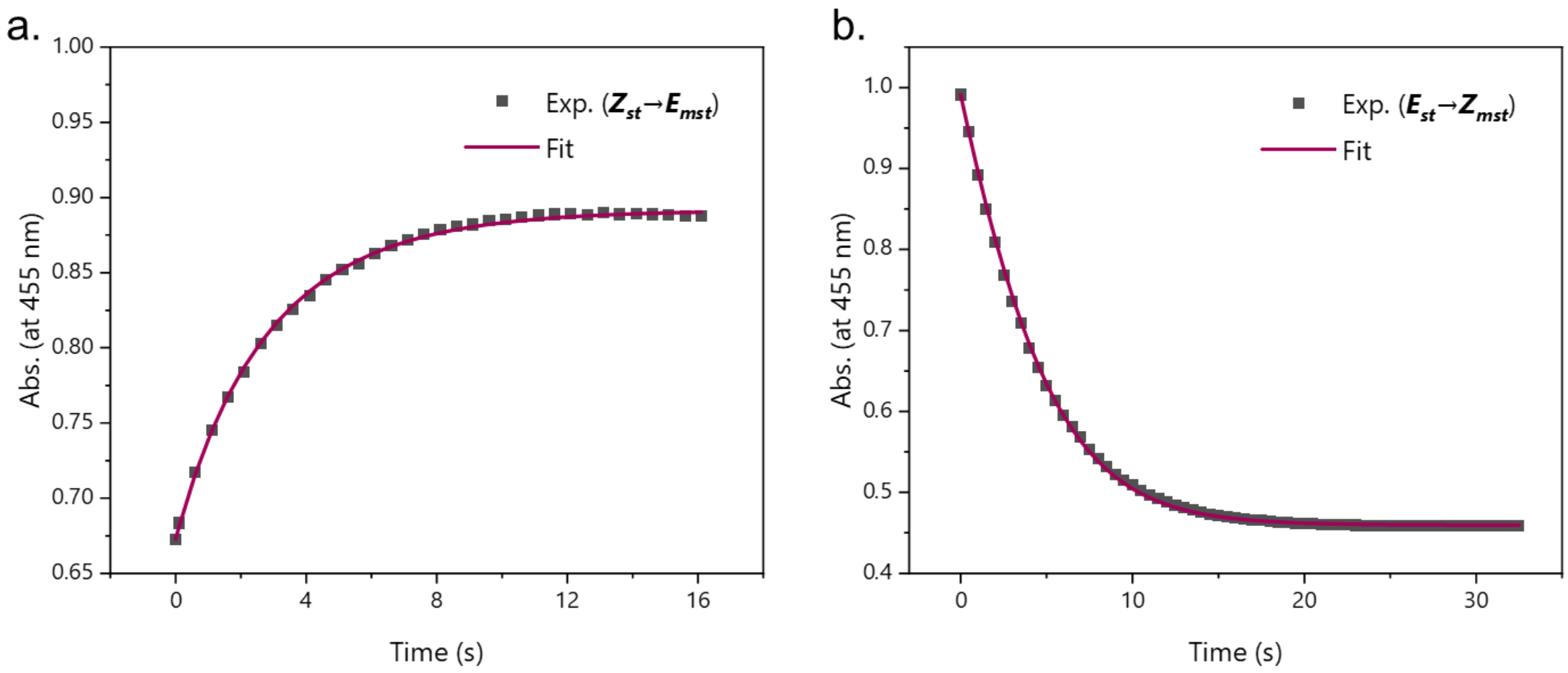

Figure S47. Evolution of the absorption at 455 nm during the irradiation of (a). ***Z**$_{st}$*-**CMBZO** and (b). ***E**$_{st}$*-**CMBZO** in THF with 455 nm light. The red line represents the fit obtained with the ODE solver from COPASI.

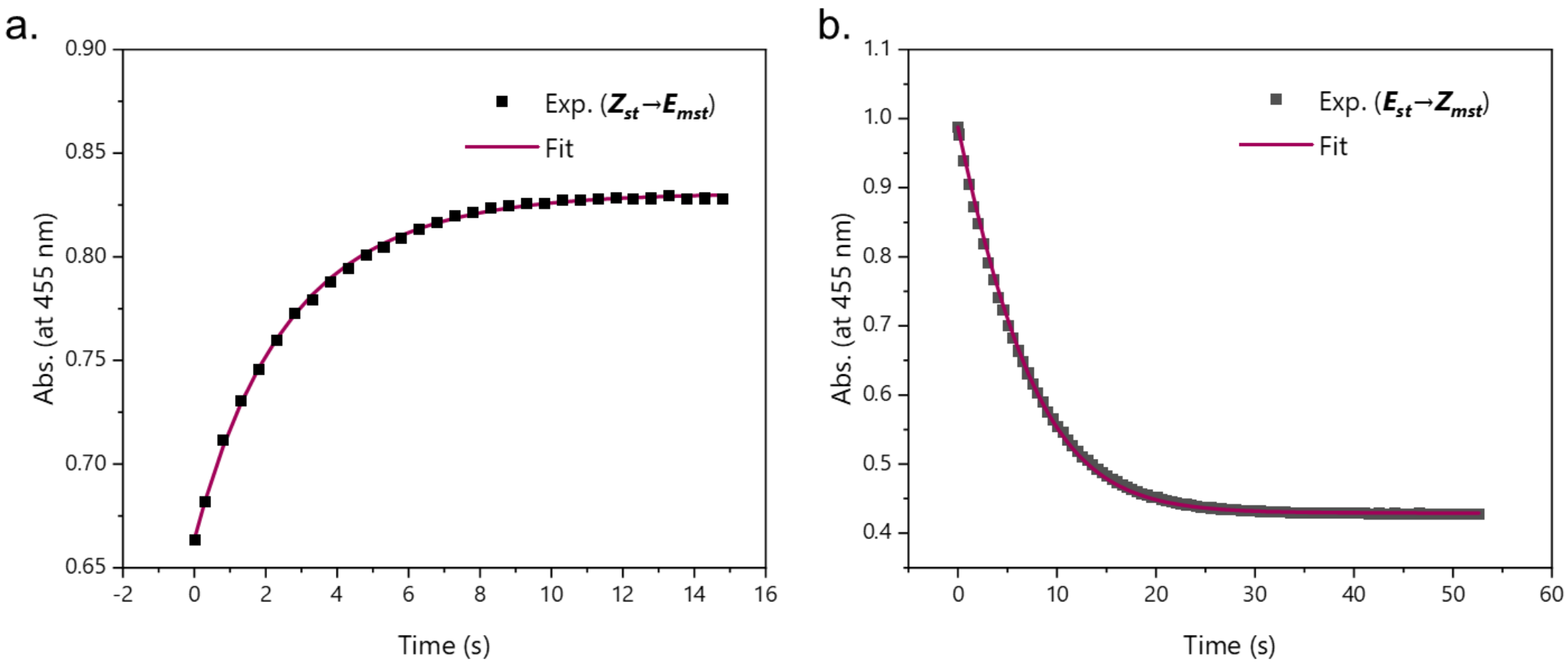

Figure S48. Evolution of the absorption at 455 nm during the irradiation of (a). ***Z**$_{st}$***-CMBID** and (b). ***E**$_{st}$***-CMBID** in THF with 455 nm light. The red line represents the fit obtained with the ODE solver from COPASI.

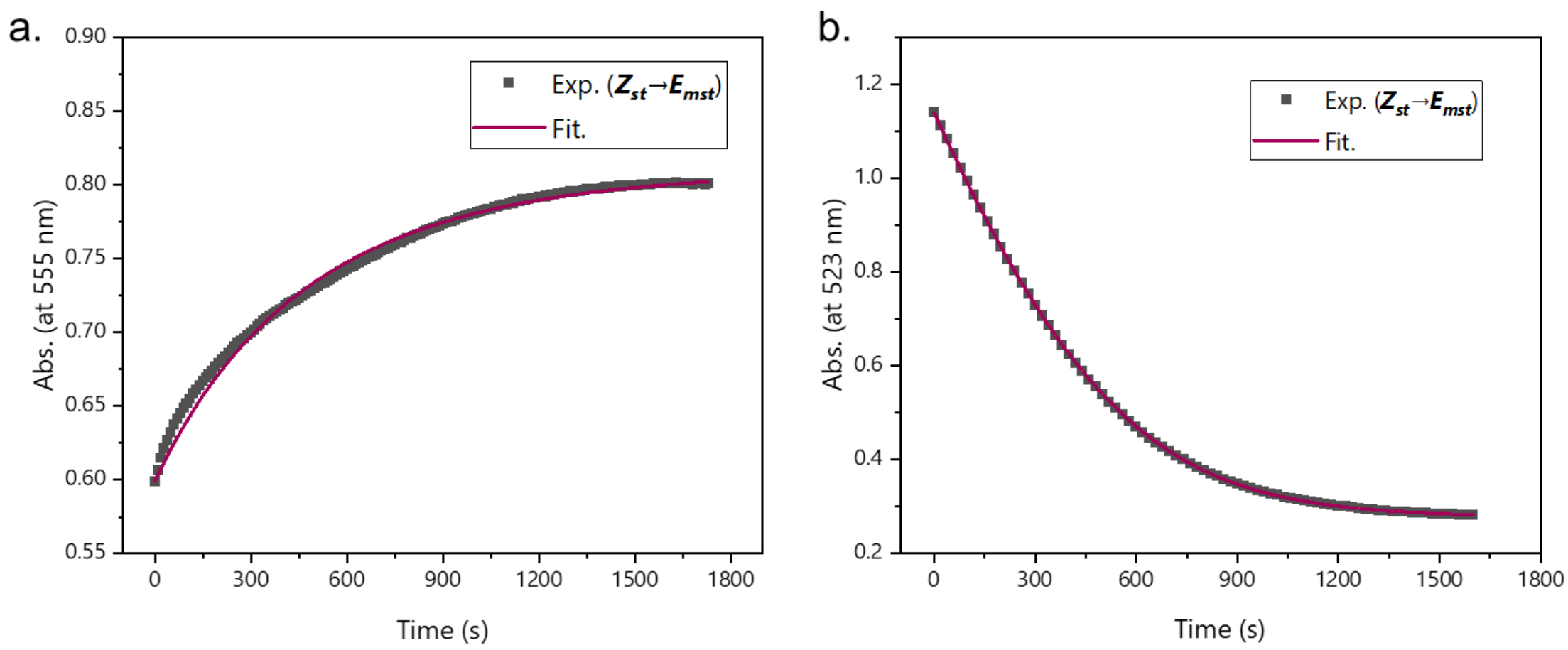

Figure S49. (a). Evolution of the absorption at 555 nm during the irradiation of ***Z**$_{st}$***-CMBZT-2H^{2+}** in DCM with 565 nm light, (b). Evolution of the absorption at 523 nm during the irradiation of ***E**$_{st}$***-CMBZT-2H^{2+}** in DCM with 530 nm light. The red line represents the fit obtained with the ODE solver from COPASI.

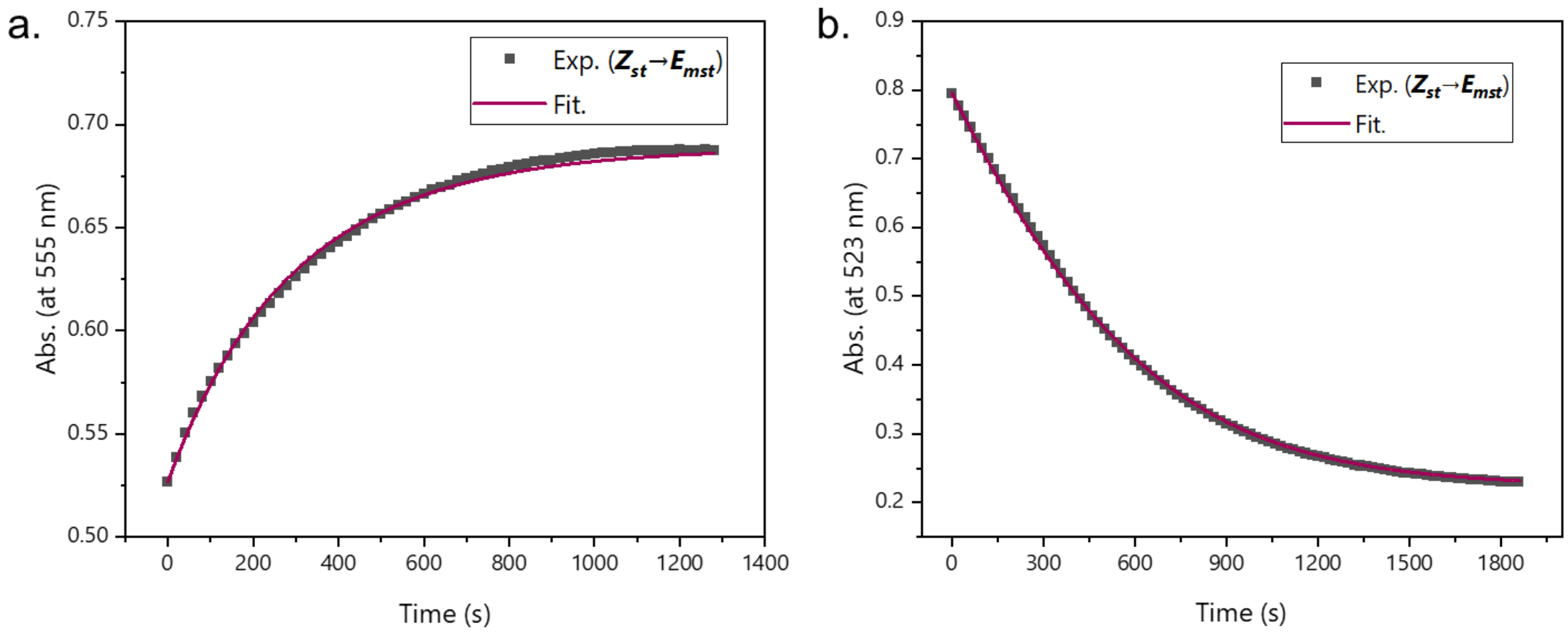

Figure S50. (a). Evolution of the absorption at 555 nm during the irradiation of ***Z**$_{st}$***-CMBZO-2H^{2+}** in DCM with 565 nm light, (b). Evolution of the absorption at 523 nm during the irradiation of ***E**$_{st}$***-CMBZO-2H^{2+}** in DCM with 530 nm light. The red line represents the fit obtained with the ODE solver from COPASI.

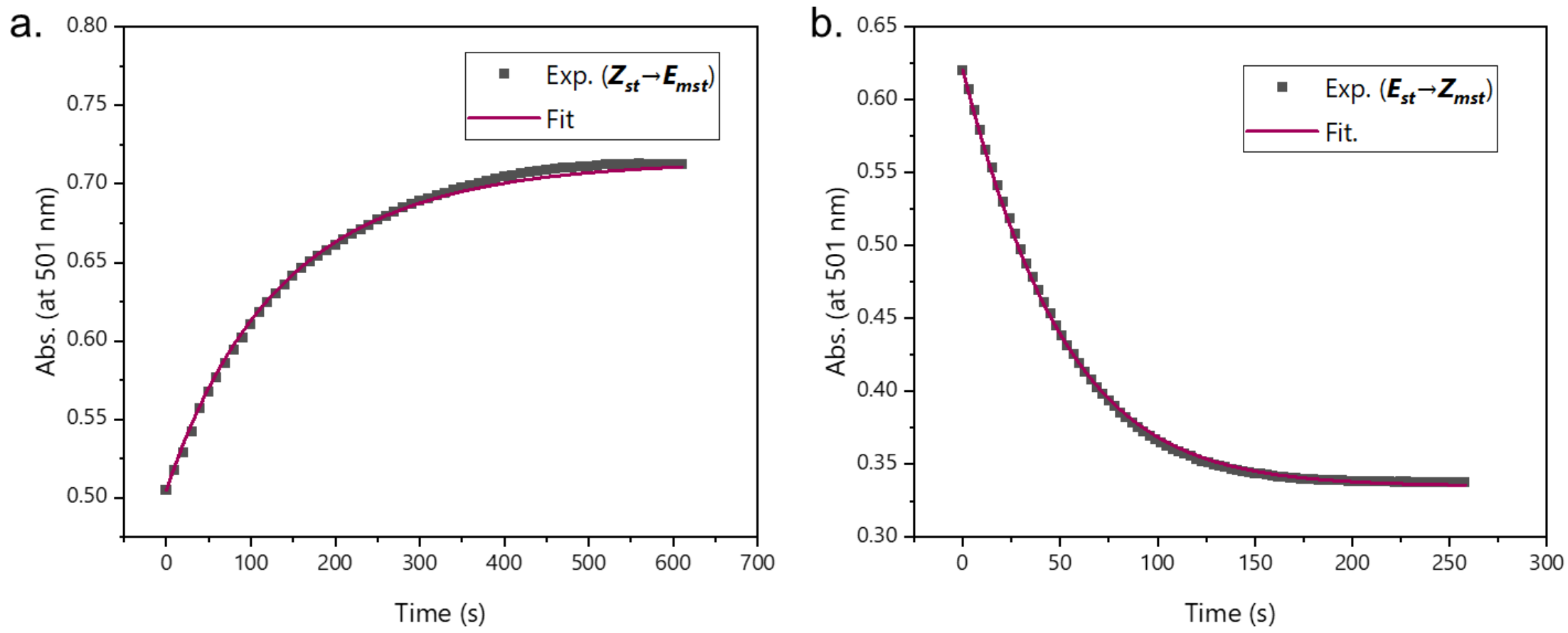

Figure S51. Evolution of the absorption at 501 nm during the irradiation of (a). ***Z**$_{st}$***-CMBID-2H^{2+}** and (b). ***E**$_{st}$***-CMBID-2H^{2+}** in DCM with 505 nm light. The red line represents the fit obtained with the ODE solver from COPASI.

Table S3. Switching quantum yield and PSS ratio of the stable and metastable diastereomers of **CMR** motors at the indicated wavelengths.

**Photo-isomerization**	$Z_{st} \rightarrow E_{mst}$			$E_{st} \rightarrow Z_{mst}$		
**CMR**[a]	Φ_{sw}[b] [%]	Φ_{ws}[b] [%]	PSS ratio[c] (mst : st)	Φ_{sw}[b] [%]	Φ_{ws}[b] [%]	PSS ratio[c] (mst : st)
**BZT**	13.5 ± 0.6	0.1	98:2	6.0 ± 0.2	0.2	> 99:1
**BZO**	19.0 ± 2.1	0.2	96:4	8.7 ± 0.2	0.2	> 99:1
**BID**	22.9 ± 2.3	0.1	97:3	6.6 ± 0.6	0.2	98:2
**BZT-2H^{2+}**	2.8 ± 0.5	0.1	98:2	8.2 ± 0.2	0.3 ± 0.2	98:2
**BZO-2H^{2+}**	5.3 ± 0.4	0.1	97:3	6.5 ± 0.3	0.1	98:2
**BID-2H^{2+}**	5.0 ± 0.1	0.1	97:3	13.8 ± 0.6	0.1	98:2

[a] Only (*S*,*S*)-(*M*, *M*) states were shown, forward switching was demonstrated in this content

[b] The error margins were calculated from experiments performed in triplicate.

[c] The molar absorption coefficients for the stable states were obtained by UV-Vis measurements. The ones for the metastable states were obtained by extracting the values from the UV-Vis spectrum at the PSS, knowing the concentration of stable and metastable species as obtained by ^{1}H-NMR.

11. Visible light-responsiveness of CMR-2H^{2+} revealed by UV/vis absorption spectra

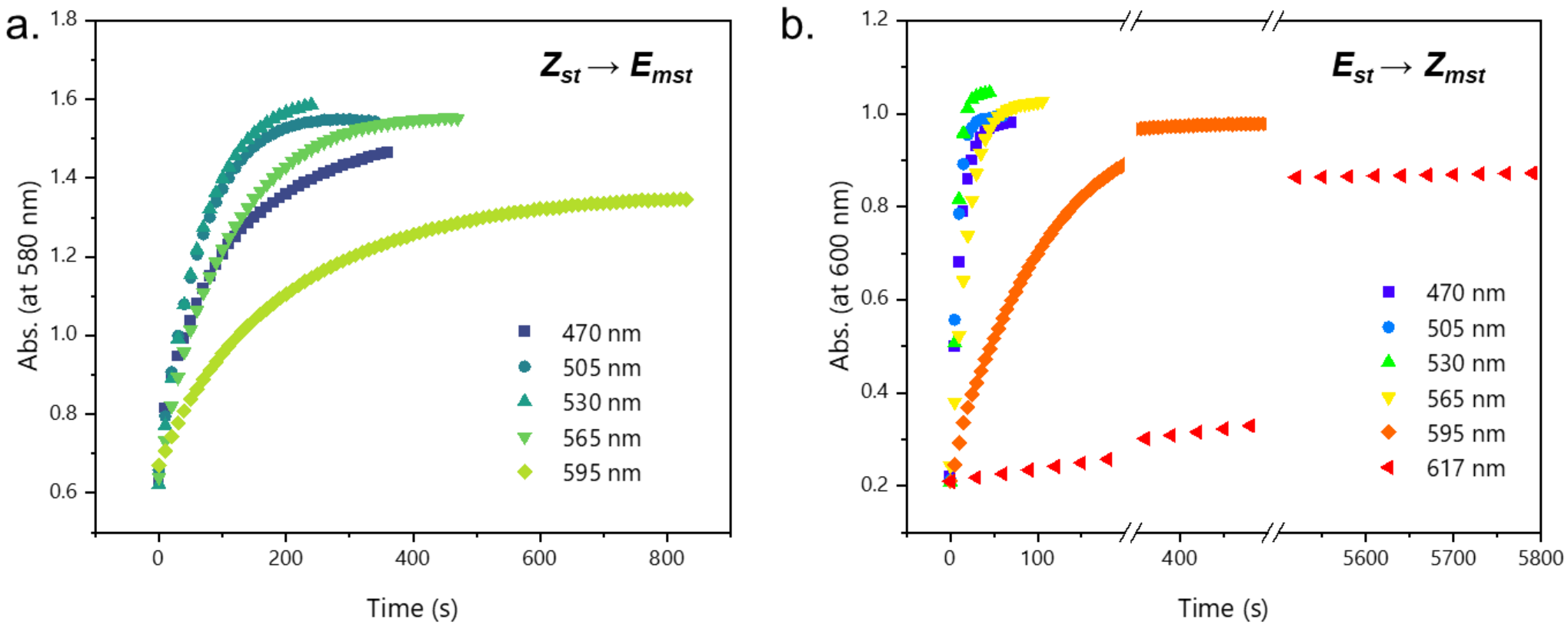

Figure S52. Evolution of the absorption changes during the irradiation of (a). ***Z*$_{st}$-CMBZT-2H^{2+}** and (b). ***E*$_{st}$-CMBZT-2H^{2+}** (2*10^{-5} M, −15 ℃ in DCM) with 470, 505, 530, 565, 595 and 617 nm light.

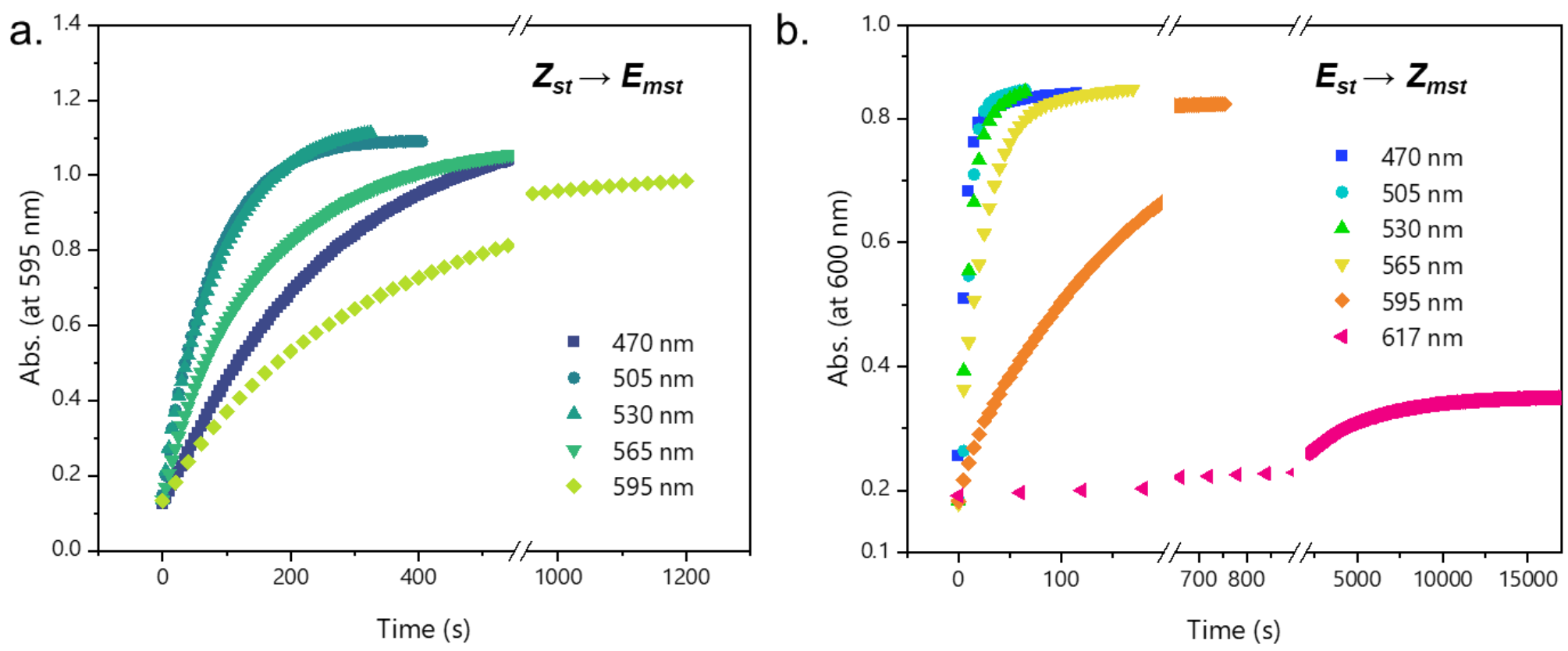

Figure S53. Evolution of the absorption changes during the irradiation of (a). ***Z*$_{st}$-CMBZO-2H^{2+}** and (b). ***E*$_{st}$-CMBZO-2H^{2+}** (2*10^{-5} M, −15 ℃ in DCM) with 470, 505, 530, 565, 595 and 617 nm light.

12. Electronic circular dichroism spectra

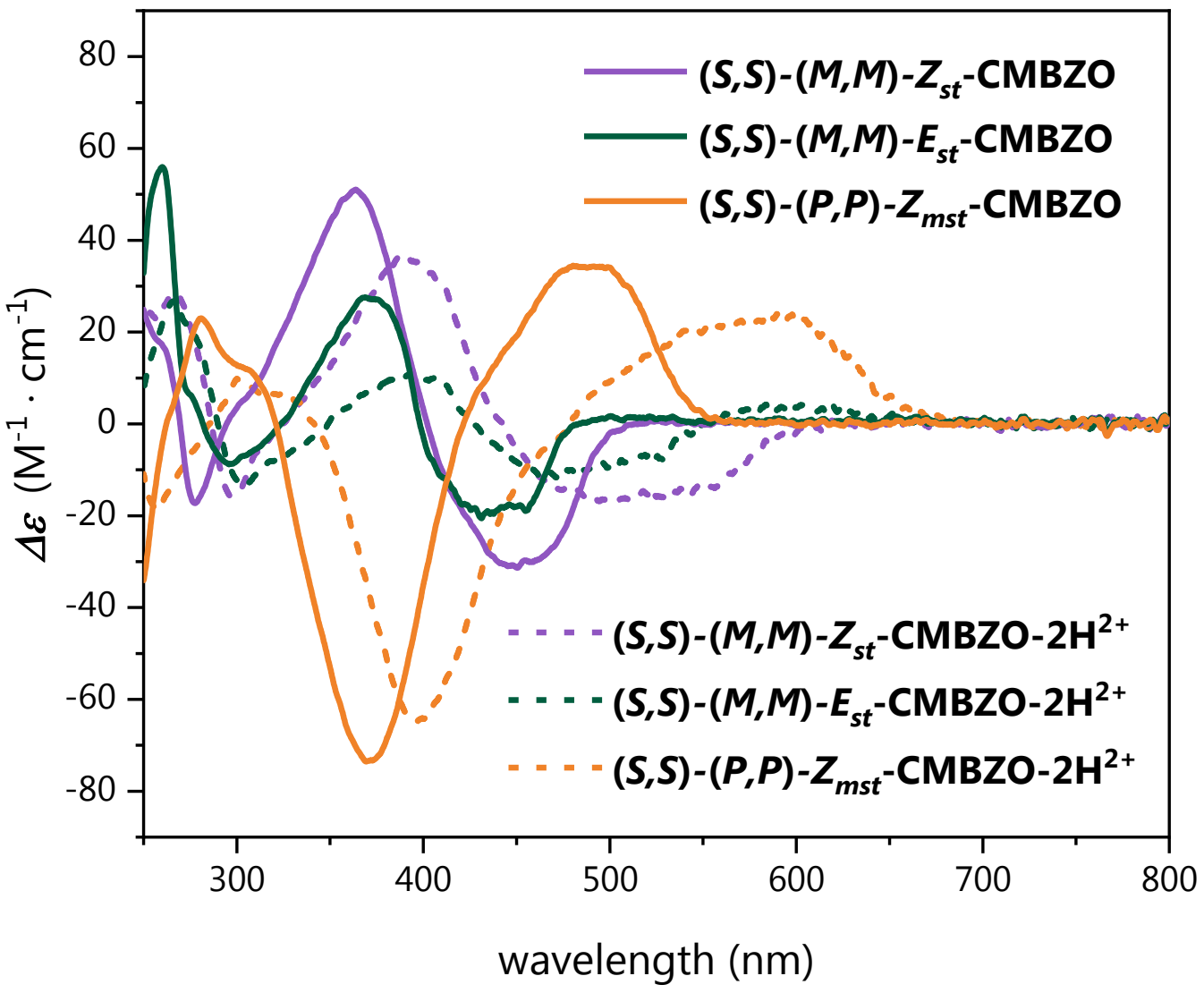

Figure S54. ECD spectra of **CMBZO** (solid line): starting from **(*S*,*S*)-(*M*,*M*)-Z_{st}-CMBZO** (purple, DCM, r.t., 20 μM), followed by 455 nm light irradiation at – 10 °C and then allowed to warm to room temperature to obtain **(*S*,*S*)-(*M*,*M*)-E_{st}-CMBZO** (blue) and subsequently irradiated at 455 nm to generate **(*S*,*S*)-(*P*,*P*)-Z_{mst}-CMBZO** (orange). ECD spectra of **CMBZO-2H^{2+}** (dash line): starting from **(*S*,*S*)-(*M*,*M*)-Z_{st}-CMBZO-2H^{2+}** (purple, DCM, r.t., 20 μM), followed by 530 nm light irradiation at – 10 °C and then warm to room temperature to obtain **(*S*,*S*)-(*M*,*M*)-E_{st}-CMBZO-2H^{2+}** (blue) and subsequently irradiated at 530 nm to generate **(*S*,*S*)-(*P*,*P*)-Z_{mst}-CMBZO-2H^{2+}** (orange).

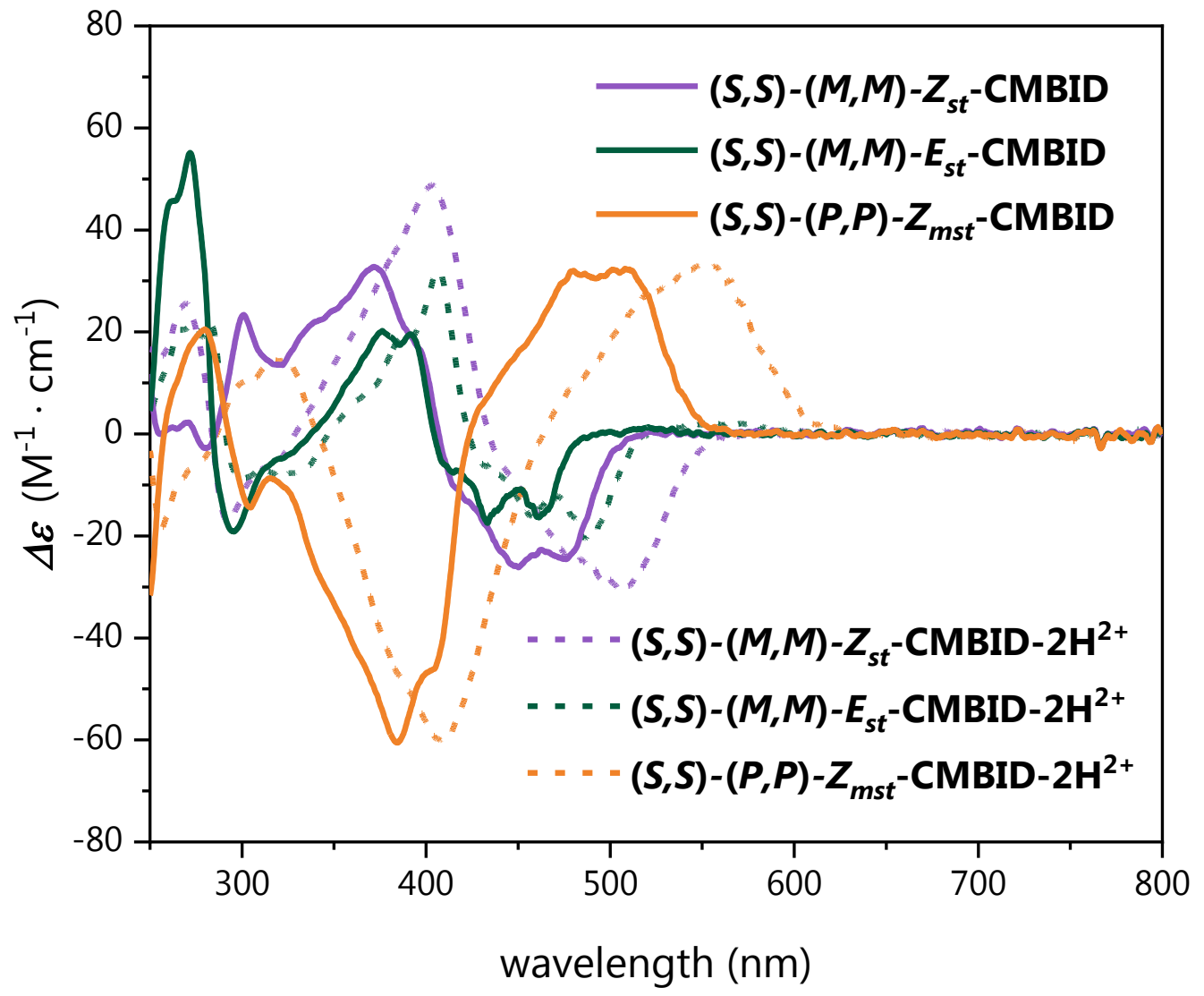

Figure S55. ECD spectra of **CMBID** (solid line): starting from **(*S*,*S*)-(*M*,*M*)-Z_{st}-CMBID** (purple, DCM, r.t., 20 μM), followed by 455 nm light irradiation at – 10 °C and then allowed to warm to room temperature to obtain **(*S*,*S*)-(*M*,*M*)-E_{st}-CMBID** (blue) and subsequently irradiated at 455 nm to generate **(*S*,*S*)-(*P*,*P*)-Z_{mst}-CMBID** (orange). ECD spectra of **CMBID-2H^{2+}** (dash line): starting from **(*S*,*S*)-(*M*,*M*)-Z_{st}-CMBID-2H^{2+}** (purple, DCM, r.t., 20 μM), followed by 505 nm light irradiation at – 10 °C and then warm to room temperature to obtain **(*S*,*S*)-(*M*,*M*)-E_{st}-CMBID-2H^{2+}** (blue) and subsequently irradiated at 505 nm to generate **(*S*,*S*)-(*P*,*P*)-Z_{mst}-CMBID-2H^{2+}** (orange).

13. UV/vis spectra of rotatory cycles of the CMR after TFA/TEA cycles

To examine whether the accumulation of triethylammonium trifluoroacetate, formed during repeated TFA/TEA neutralization cycles, interferes with the photoisomerization and thermal helix inversion processes of the coumarin motor derivatives, UV/vis switching experiments were performed after the samples had undergone three acid/base cycles. Under the dilute spectroscopic conditions, the accumulated salts remained in the millimolar concentration range, and no precipitation was observed after acid/base cycling.

After salt accumulation, the samples were kept at room temperature. Upon light irradiation, the ***Z**$_{st}$***-CMR** state undergo ¾ unidirectional cycles to reach the PSS- ***Z**$_{mst}$***-CMR** state (step 1, Figure S56). Subsequent heating of the solution at 50 °C for 3 h promoted the second thermal helix inversion (step 2, Figure S56), leading to the recovery of the THI-***Z**$_{st}$***-CMR** state with spectral features comparable to those observed before salt accumulation (Figures S57-S59). These results indicate that the accumulated triethylammonium trifluoroacetate does not significantly compromise the motor's photochemical switching or thermal helix inversion across the tested acid/base cycles.

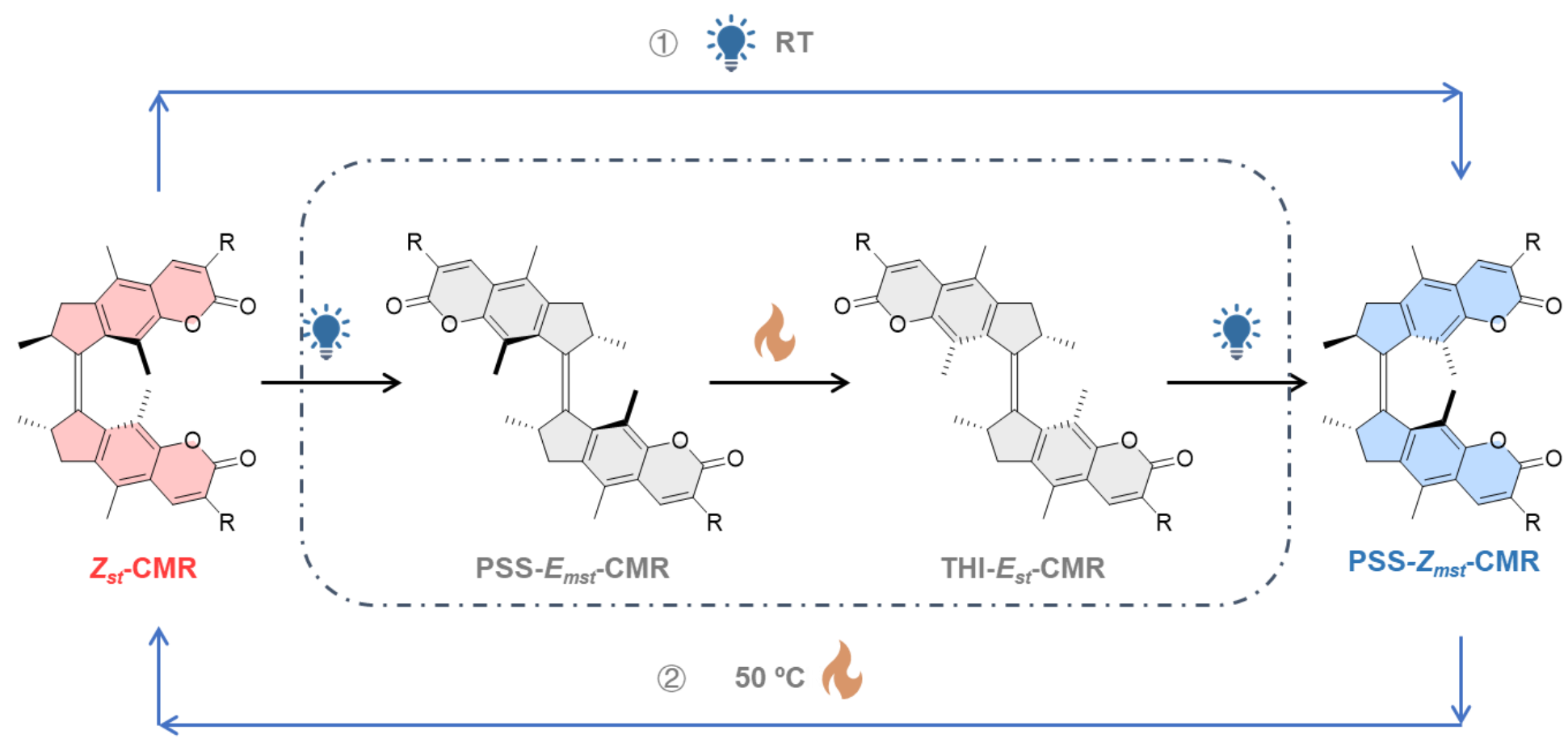

Figure S56. Scheme of the motor rotatory process from (1) ***Z**$_{st}$***-CMR** to PSS-***Z**$_{mst}$***-CMR** at room temperature (RT) upon irridation and (2) PSS-***Z**$_{mst}$***-CMR** to THI-***Z**$_{st}$***-CMR** upon warming up to 50 ºC for 5 h.

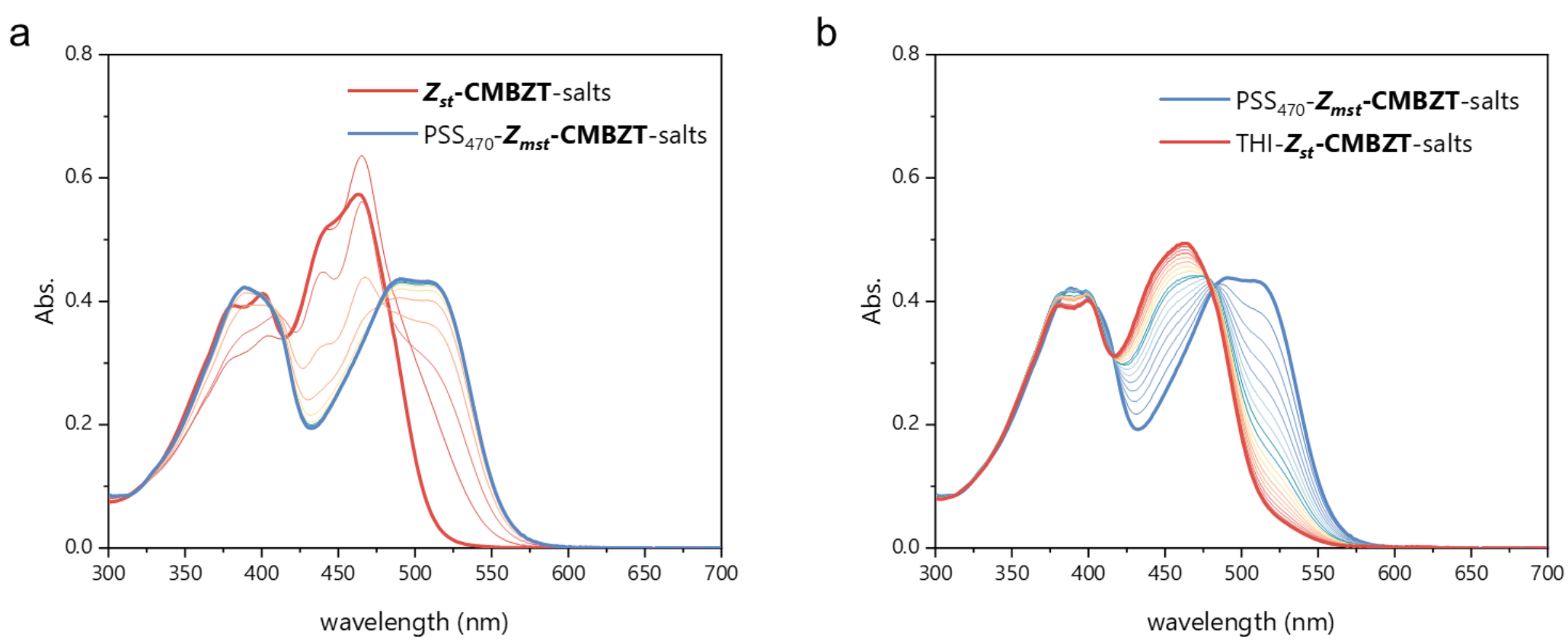

Figure S57. (a). Changes in the UV/Vis spectra of ***Z_{st}*-CMBZT** with salts (red line, 1.0*10^{-5} M in THF) upon irradiation at 470 nm at room temperature, which shows the formation of predominantly PSS_{470}-***Z_{mst}*-CMBZT** (blue line), (b). Changes in the UV/Vis spectra of PSS_{470}-***Z_{mst}*-CMBZT** with salts (blue line, 1.0*10^{-5} M in THF) upon heating up to 50 ºC for 5 h, which shows the recovery of **THI-*Z_{st}*-CMBZT** (red line).

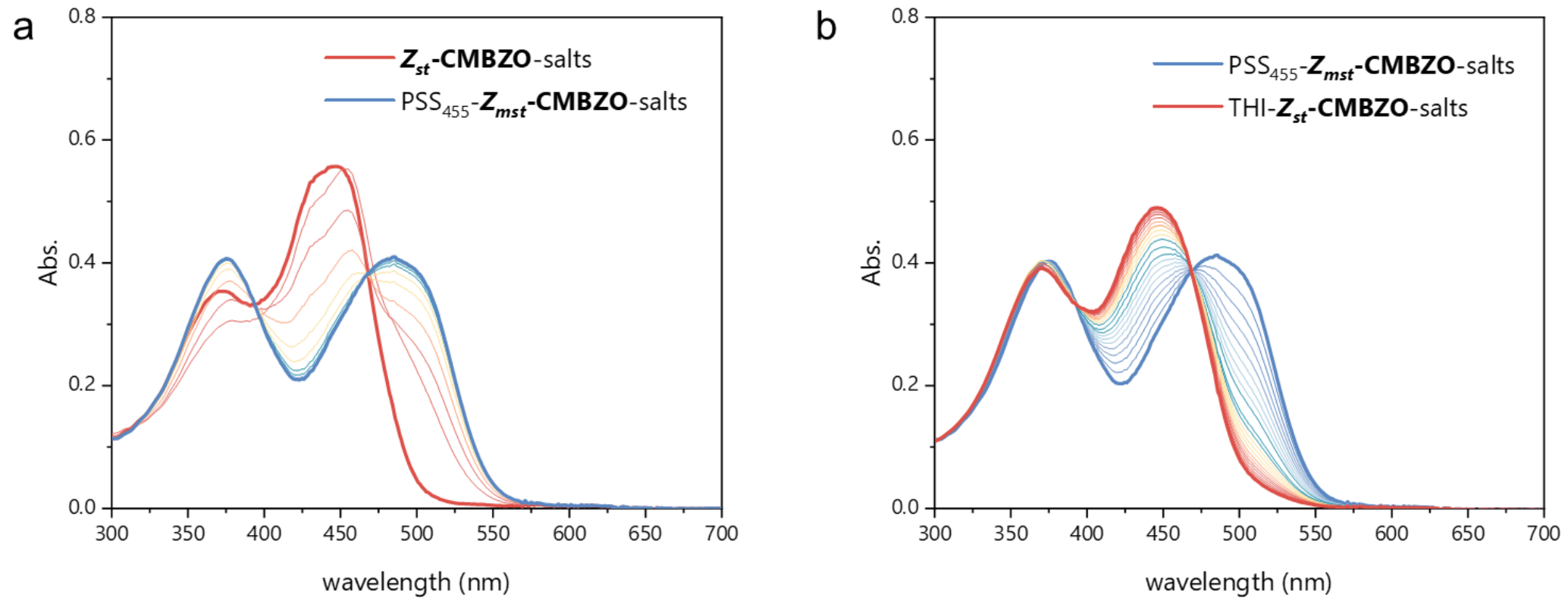

Figure S58. (a). Changes in the UV/Vis spectra of ***Z_{st}*-CMBZO** with salts (red line, 1.0*10^{-5} M in THF) upon irradiation at 455 nm at room temperature, which shows the formation of predominantly PSS_{455}-***Z_{mst}*-CMBZO** (blue line), (b). Changes in the UV/Vis spectra of PSS_{455}-***Z_{mst}*-CMBZO** with salts (blue line, 1.0*10^{-5} M in THF) upon heating up to 50 ºC for 5 h, which shows the recovery of **THI-*Z_{st}*-CMBZO** (red line).

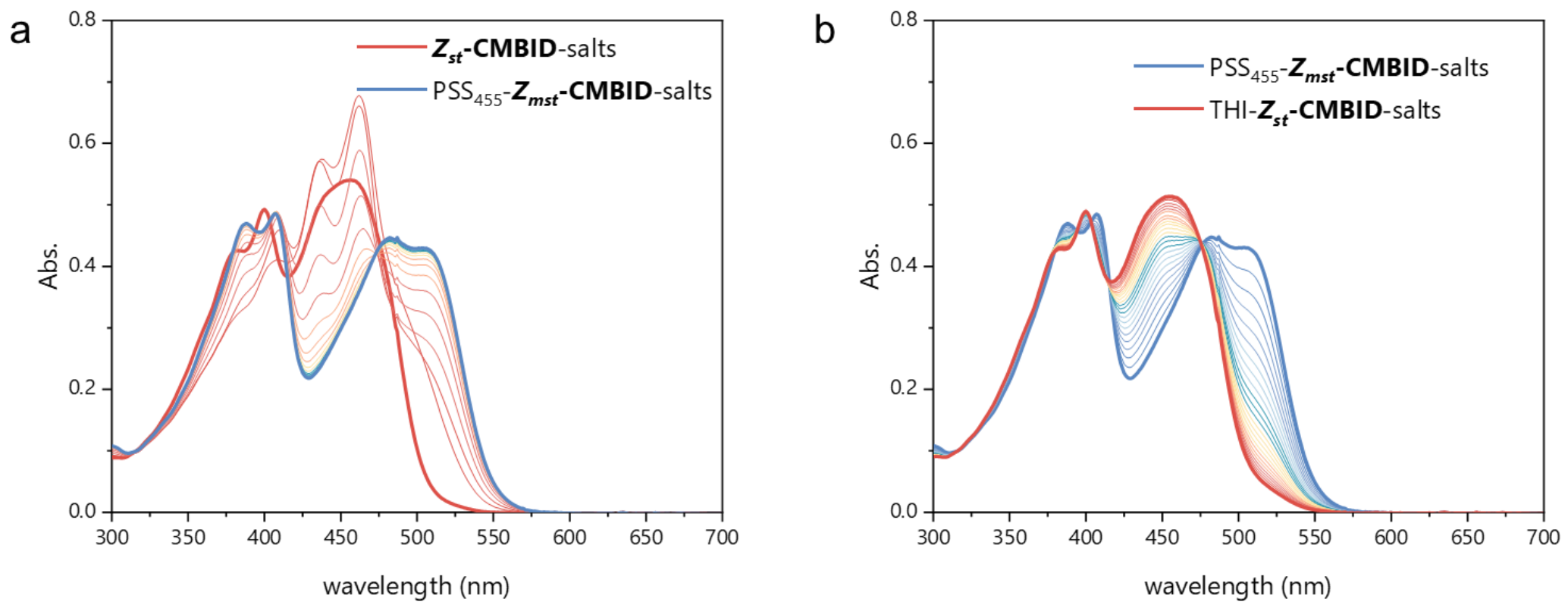

Figure S59. (a). Changes in the UV/Vis spectra of ***Z_{st}*-CMBID** with salts (red line, 1.0*10^{-5} M in THF) upon irradiation at 455 nm at room temperature, which shows the formation of predominantly PSS_{455}-***Z_{mst}*-CMBID** (blue line), (b). Changes in the UV/Vis spectra of PSS_{455}-***Z_{mst}*-CMBID** with salts (blue line, 1.0*10^{-5} M in THF) upon heating up to 50 ºC for 5 h, which shows the recovery of **THI-*Z_{st}*-CMBID** (red line).

14. Computational Analysis

The ground-state geometries of (*S*,*S*)-(*M*,*M*)-enantiomers of molecular motors with both stable-*cis* (***Z*$_{st}$**) and stable-*trans* (***E*$_{st}$**) forms were carried out based on the density functional theory (DFT) methods at the B3LYP-D3(BJ)[13-16]/6-311+g(d,p)[17] level using the Gaussian 16 program.[18] Analytical frequency calculations were also performed at the same level of theory to confirm the local minimum point of the optimized structures. In addition, the solvation model based on the SMD continuum solvent model (THF for **CMR**, DCM for **CMR-2H^{2+}**) and self-consistent reaction field (SCRF) was considered in the calculations.[19] Time-dependent density functional theory (TD-DFT) calculations were performed at the B3LYP-D3(BJ)/Def2-TZVP[20] level with the SMD continuum solvent model for THF/DCM on the optimized geometries. All the calculations were performed at 293.15 K unless otherwise mentioned. Absorption spectra were simulated via TD-DFT calculations of the lowest 50 vertical singlet electronic excitations with Gaussian broadening for the oscillator strength *f* and rotatory strength R_{vel} (in velocity form), and half-width at half-height was set at 0.4 eV. No special shift or scaling was applied unless otherwise mentioned. The isosurfaces of molecular orbitals (MOs) were drawn using IQmol 2.15 program[21] with the isovalue set to 0.05 Å^{-3}. All coordinates of optimized geometries of these molecular motors are provided in the corresponding excel file as the supporting file.

Table S4. Summary of the Gibbs free energies (*G*), relative energies and the corresponding proportion of different conformers of **CMBZT** and **CMBZT-2H^{2+}**.

Compound	Conformer	*G* (Hartree)	*Rel-ΔG* (kcal mol^{-1})	Pop. (%)[a]
**(*S*,*S*)-(*M*,*M*)-Z_{st}-CMBZT**	**a**	-2904.272047	9.2	< 0.1
	**b**	-2904.280438	4.0	< 0.1
	**c**	-2904.286774	0	> 99.8
**(*S*,*S*)-(*M*,*M*)-E_{st}-CMBZT**	**a**	-2904.267738	8.6	< 0.1
	**b**	-2904.272915	5.4	< 0.1
	**c**	-2904.281457	0	> 99.8
**(*S*,*S*)-(*M*,*M*)-Z_{st}-CMBZT-2H^{2+}**	**a**	-2905.148454	0	92.5
	**b**	-2905.145425	1.9	7.4
	**c**	2905.141228	4.5	< 0.1
**(*S*,*S*)-(*M*,*M*)-E_{st}-CMBZT-2H^{2+}**	**a**	-2905.144525	0	> 99.8
	**b**	-2905.138212	4.0	< 0.1
	**c**	-2905.136010	5.3	< 0.1

a. The populations were estimated at 298.15 K by Boltzmann distribution law based on the Gibbs free energies with the degeneracy of **Conformer-b** as 2.

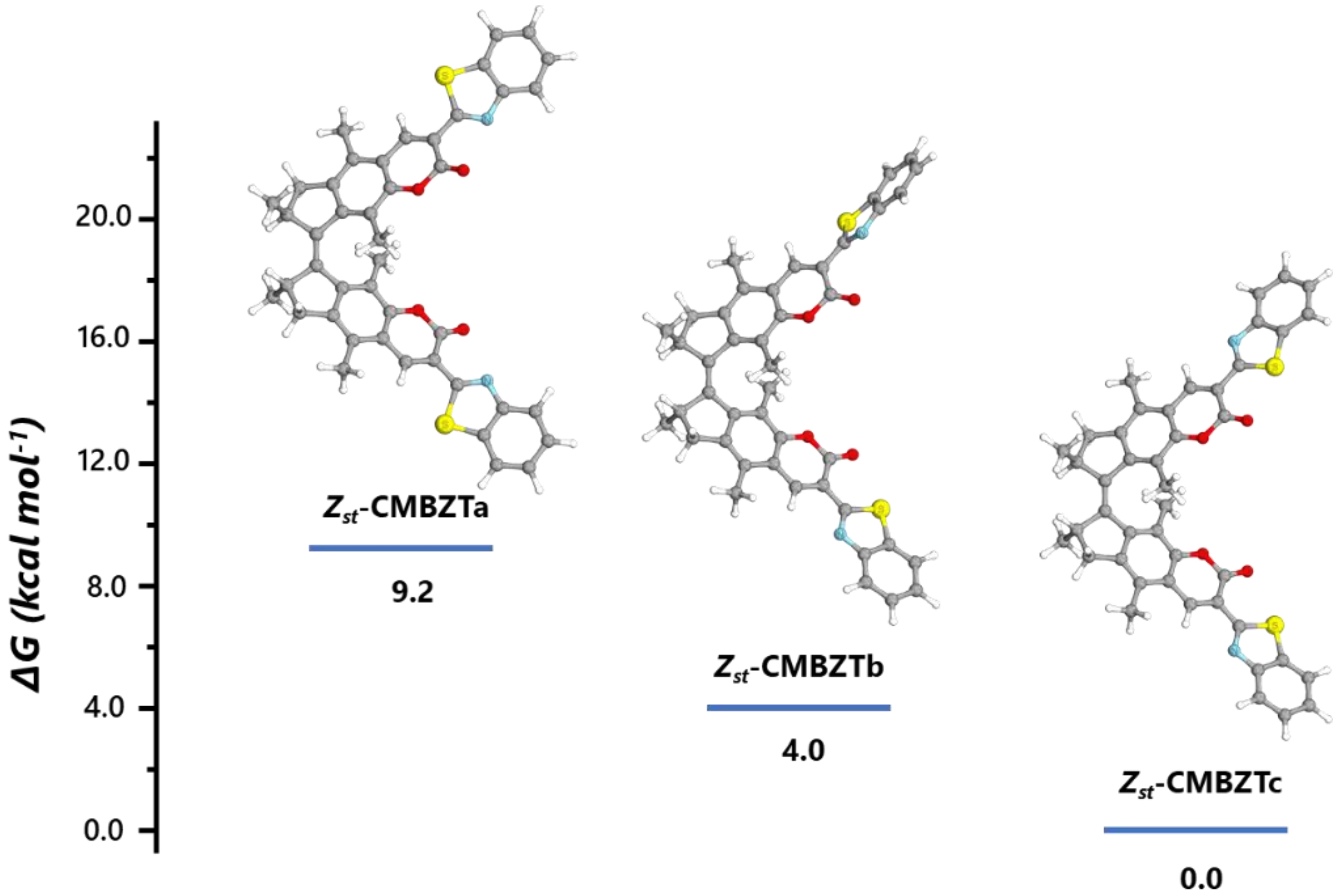

Figure S60. Optimized geometries of different conformers with free energy diagram of **(*S*,*S*)-(*M*,*M*)-*Z*$_{st}$-CMBZT**, color codes: *S* = yellow; *O* = red; *N* = blue, respectively.

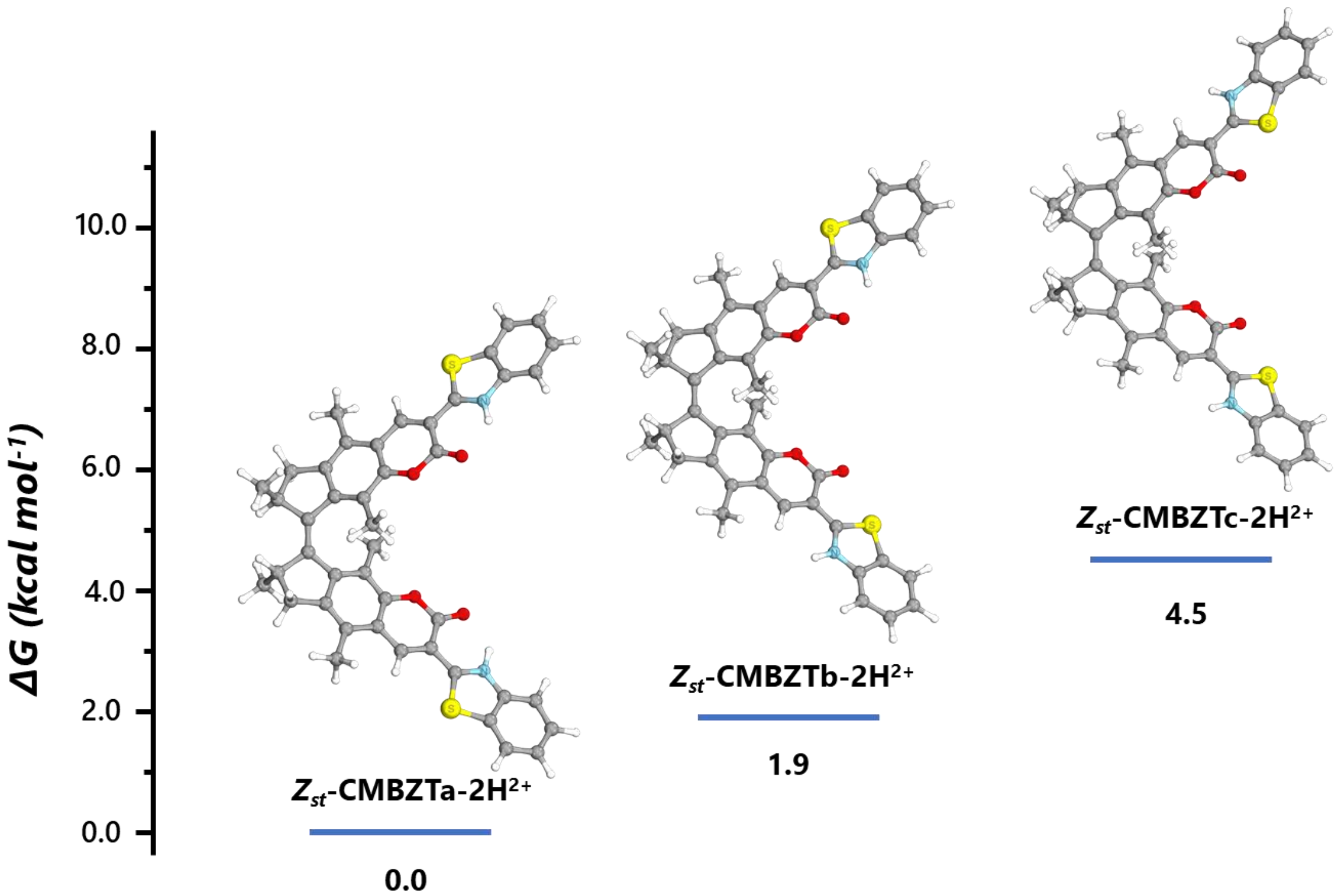

Figure S61. Optimized geometries of different conformers with free energy diagram of **(*S*,*S*)-(*M*,*M*)-*Z*$_{st}$-CMBZT-2H^{2+}**, color codes: *S* = yellow; *O* = red; *N* = blue, respectively.

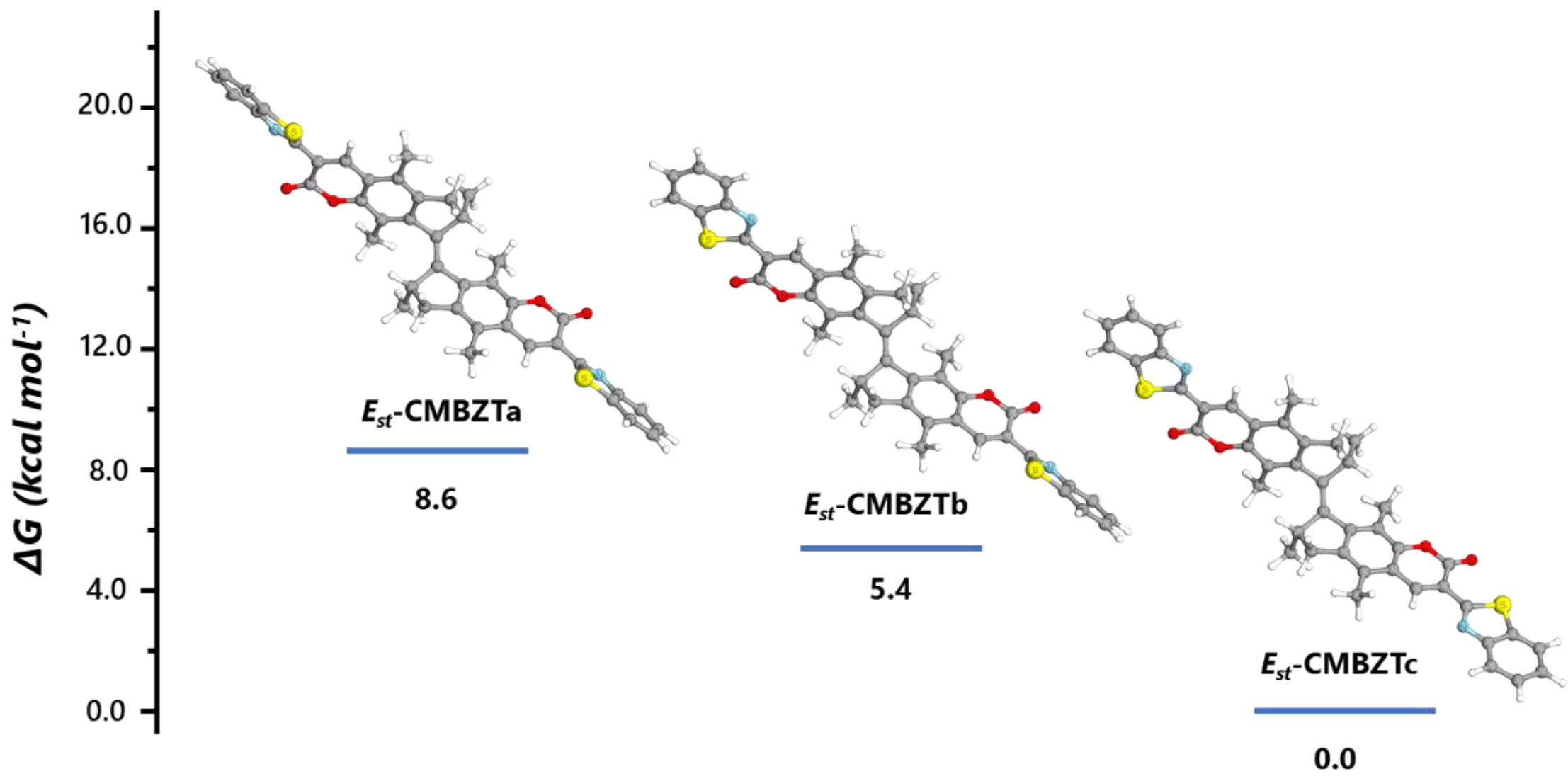

Figure S62. Optimized geometries of different conformers with free energy diagram of **(*S*,*S*)-(*M*,*M*)-E_{st}-CMBZT**, color codes: *S* = yellow; *O* = red; *N* = blue, respectively.

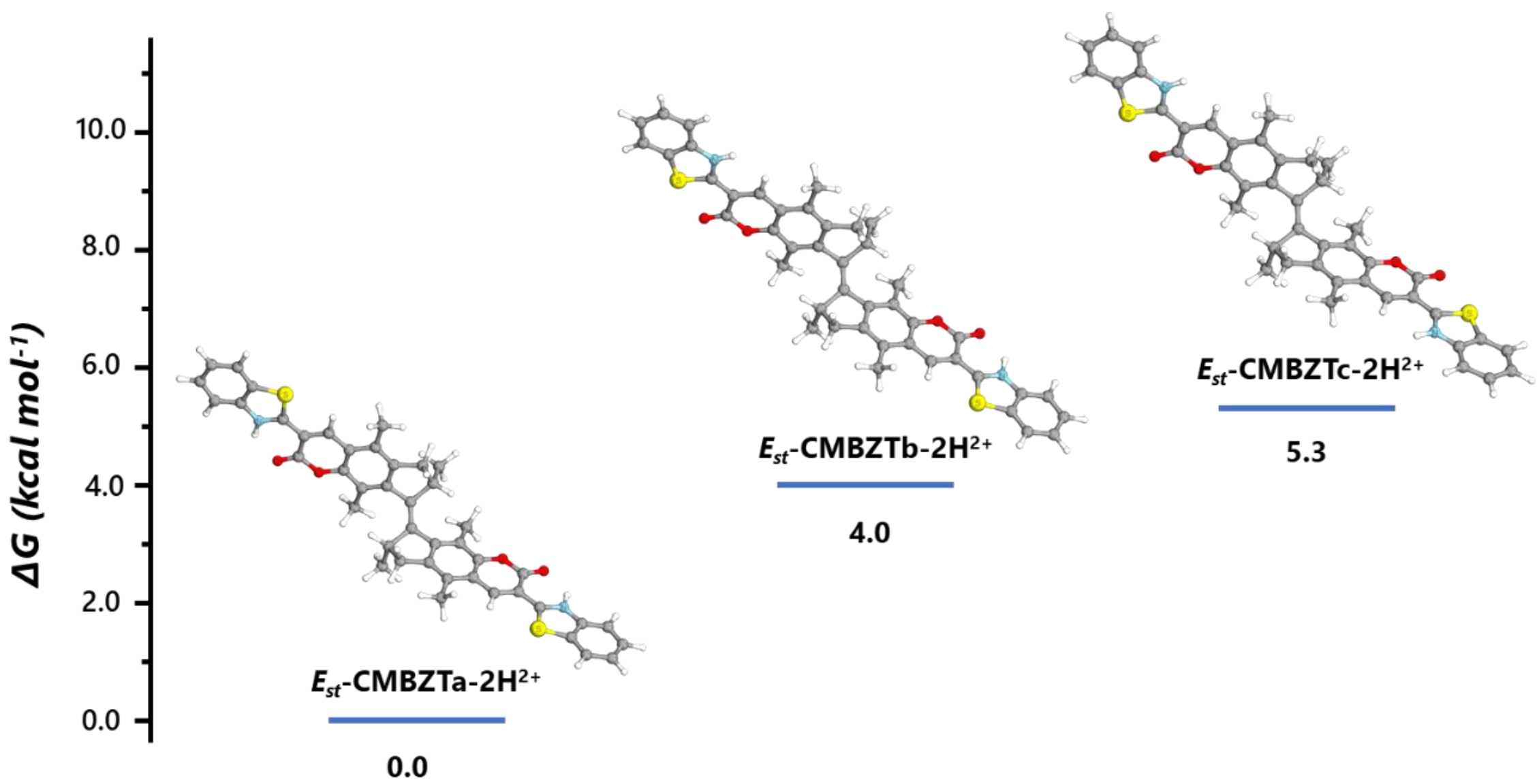

Figure S63. Optimized geometries of different conformers with free energy diagram of **(*S*,*S*)-(*M*,*M*)-E_{st}-CMBZT-2H^{2+}**, color codes: *S* = yellow; *O* = red; *N* = blue, respectively.

Table S5. Summary of the Gibbs free energies (*G*), relative energies and the corresponding proportion of different conformers of **CMBZO** and **CMBZO-2H^{2+}**.

Compound	Conformer	*G* (Hartree)	*Rel-ΔG* (kcal mol^{-1})	Pop. (%)[a]
**(*S*,*S*)-(*M*,*M*)-Z_{st}-CMBZO**	**a**	-2258.296819	0.72	11.8
	**b**	-2258.297503	0.29	48.6
	**c**	-2258.297969	0	39.6
**(*S*,*S*)-(*M*,*M*)-E_{st}-CMBZO**	**a**	-2258.289929	1.3	5.2
	**b**	-2258.291549	0.4	47.8
	**c**	-2258.292118	0	47.0
**(*S*,*S*)-(*M*,*M*)-Z_{st}-CMBZO-2H^{2+}**	**a**	-2259.165209	0	> 99.8
	**b**	-2259.155720	6.0	< 0.1
	**c**	-2259.147034	11.4	< 0.1
**(*S*,*S*)-(*M*,*M*)-E_{st}-CMBZO-2H^{2+}**	**a**	-2259.159173	0	> 99.8
	**b**	-2259.148859	6.5	< 0.1
	**c**	-2259.136178	14.4	< 0.1

a. The populations were estimated at 298.15 K by Boltzmann distribution law based on the Gibbs free energies with the degeneracy of **Conformer-b** set as 2.

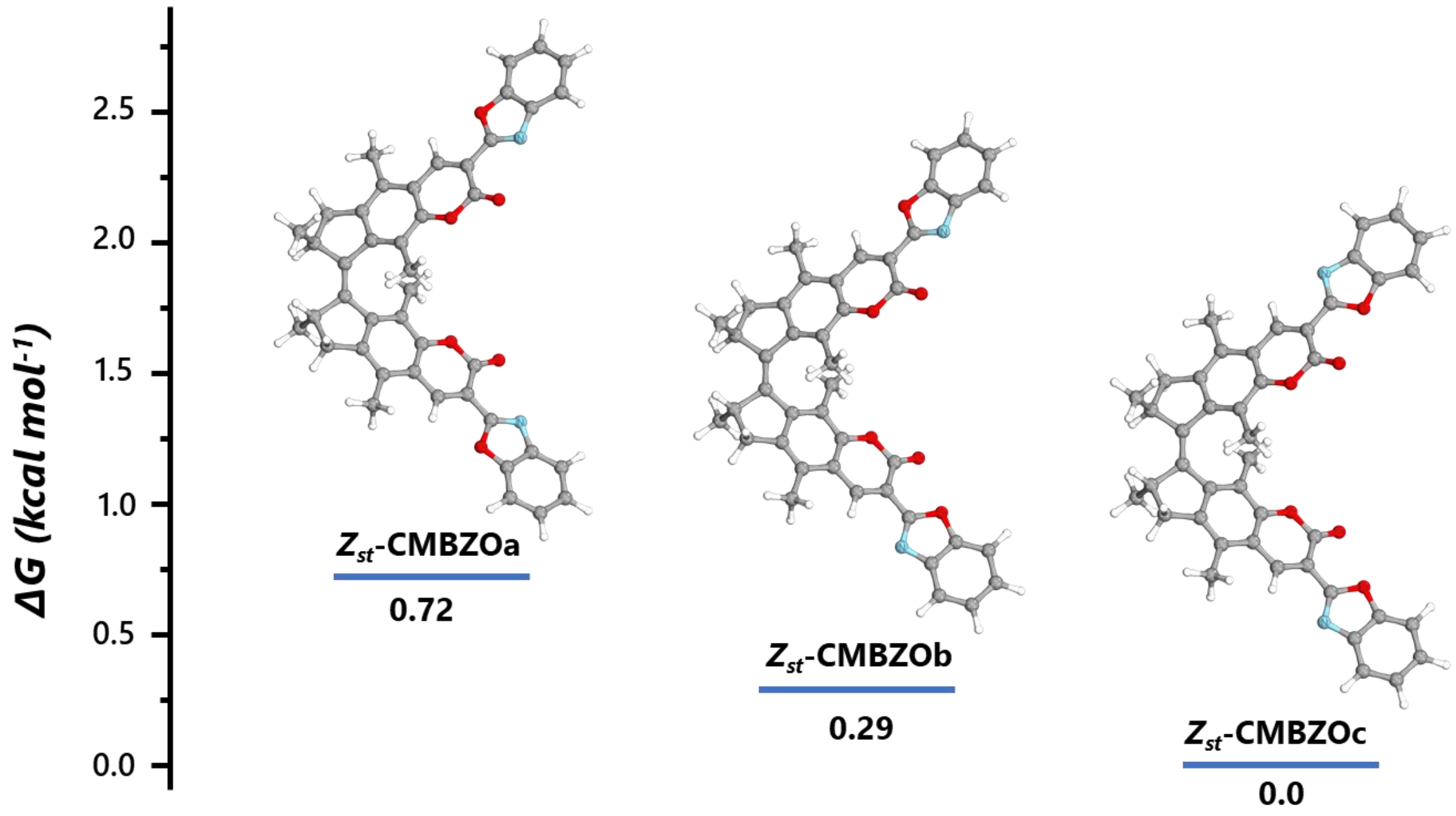

Figure S64. Optimized geometries of different conformers with free energy diagram of **(*S*,*S*)-(*M*,*M*)-Z_{st}-CMBZO**, color codes: *O* = red; *N* = blue, respectively.

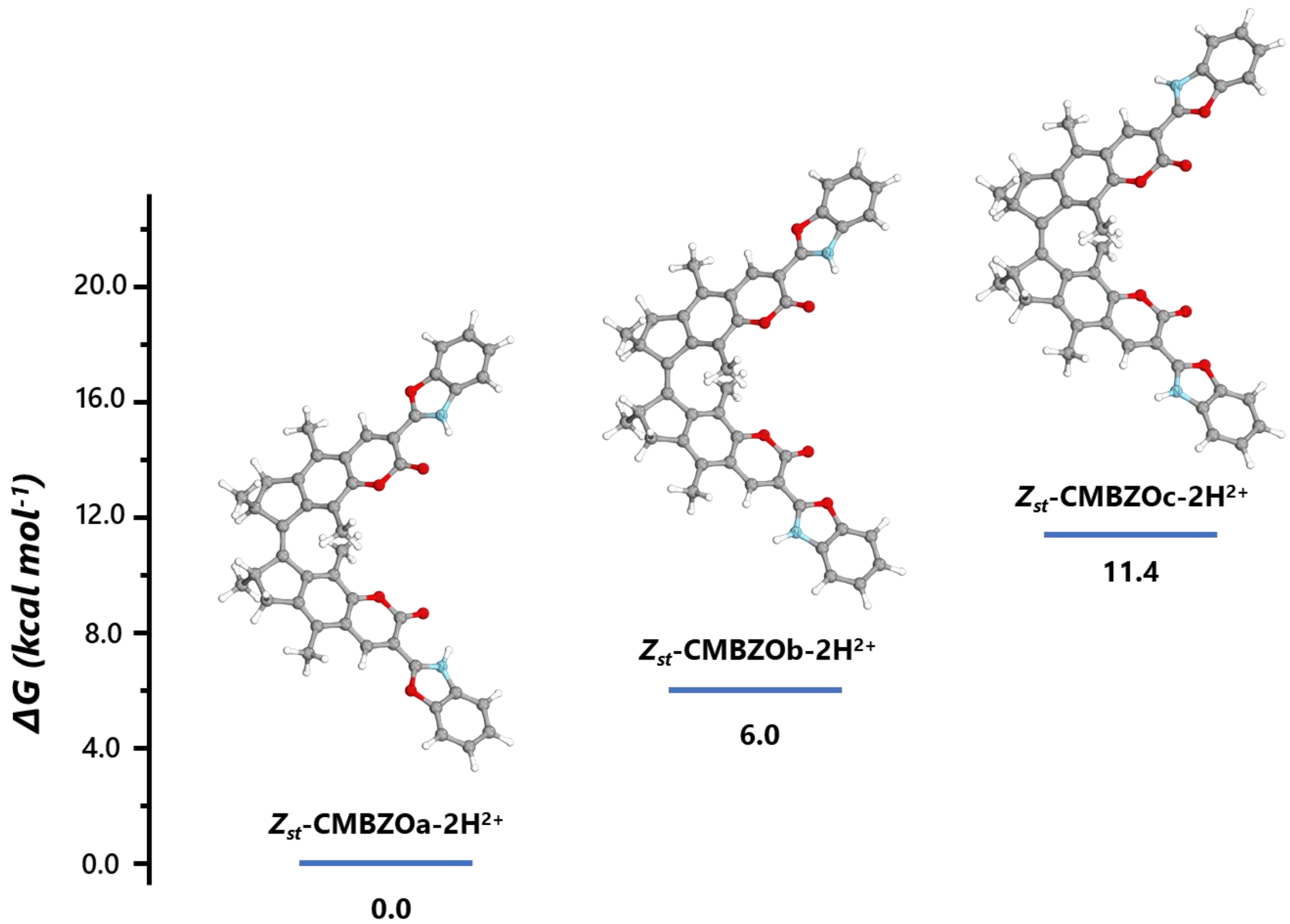

Figure S65. Optimized geometries of different conformers with free energy diagram of **(*S*,*S*)-(*M*,*M*)-Z_{st}-CMBZO-2H^{2+}**, color codes: *O* = red; *N* = blue, respectively.

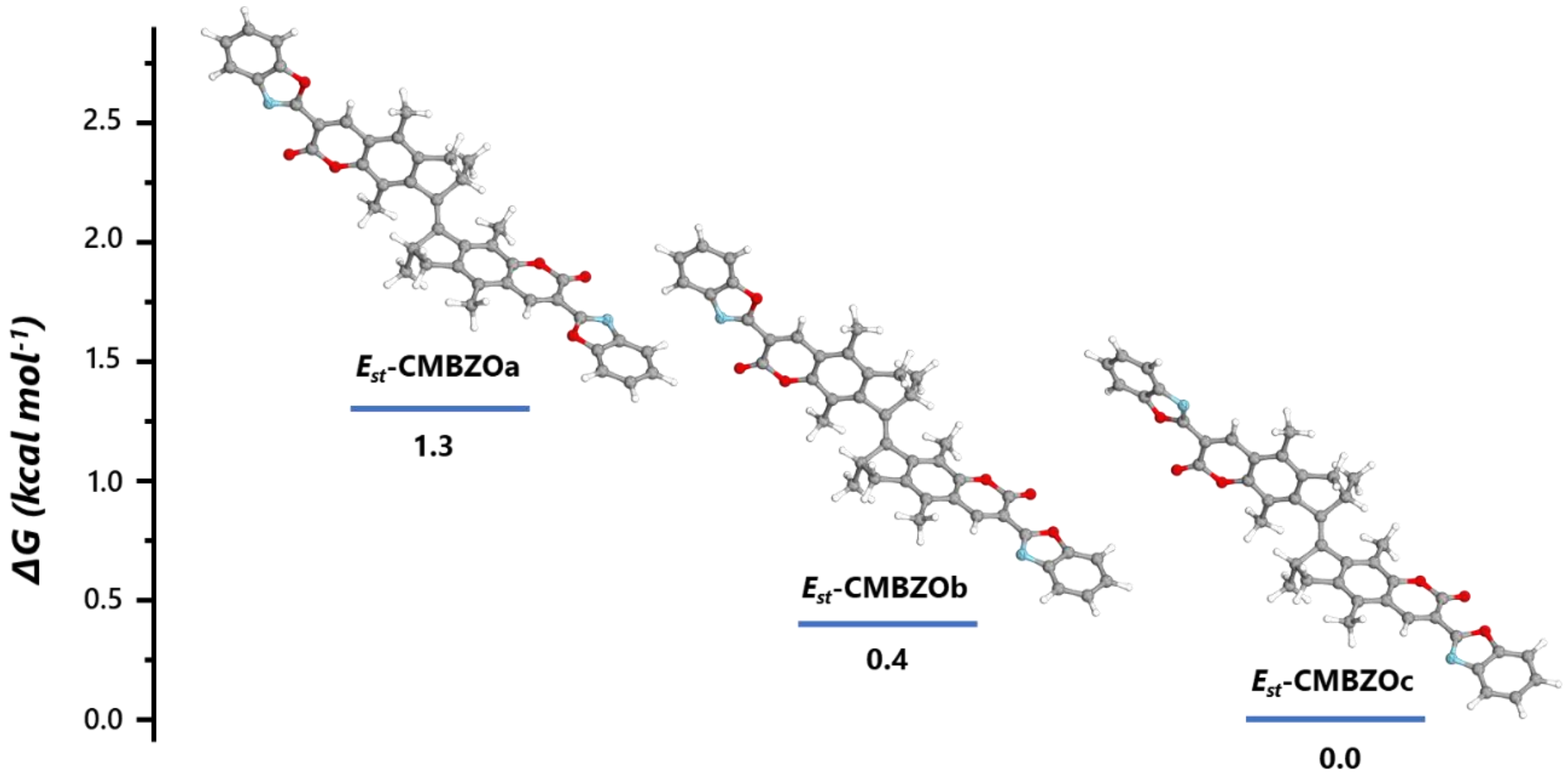

Figure S66. Optimized geometries of different conformers with free energy diagram of **(*S*,*S*)-(*M*,*M*)-*E*$_{st}$-CMBZO**, color codes: *O* = red; *N* = blue, respectively.

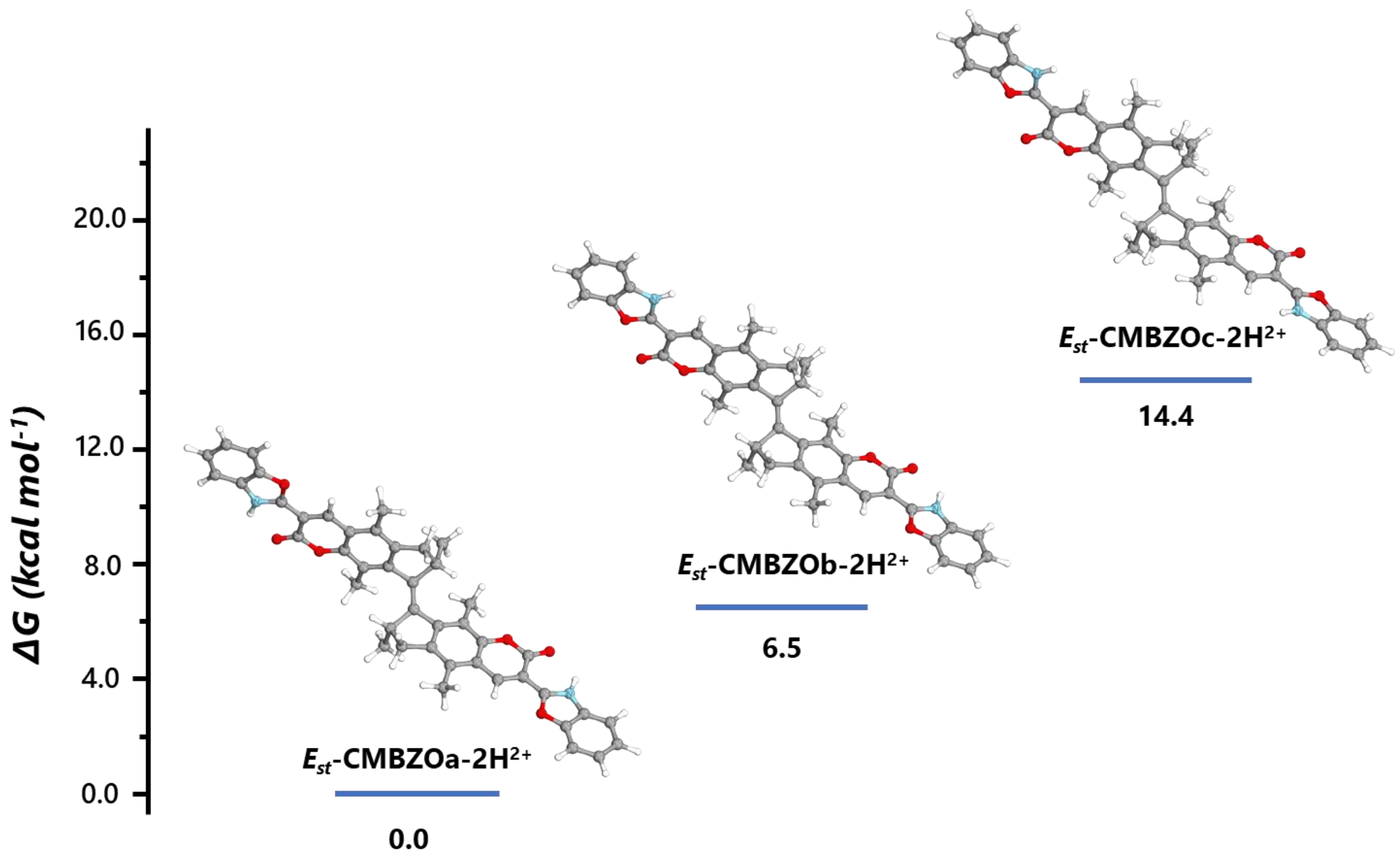

Figure S67. Optimized geometries of different conformers with free energy diagram of **(*S*,*S*)-(*M*,*M*)-*E*$_{st}$-CMBZO-2H^{2+}**, color codes: *O* = red; *N* = blue, respectively.

Table S6. Summary of the Gibbs free energies (*G*), relative energies and the corresponding proportion of different conformers of **CMBID** and **CMBID-2H^{2+}**.

Compound	Conformer	*G* (Hartree)	*Rel-ΔG* (kcal mol^{-1})	Pop. (%)[a]
**(*S*,*S*)-(*M*,*M*)-Z_{st}-CMBID**	**a**	-2218.563253	13.4	< 0.1
	**b**	-2218.573771	6.8	< 0.1
	**c**	-2218.584672	0	> 99.8
**(*S*,*S*)-(*M*,*M*)-E_{st}-CMBID**	**a**	-2218.555195	14.7	< 0.1
	**b**	-2218.569135	5.9	< 0.1
	**c**	-2218.578593	0	> 99.8
**(*S*,*S*)-(*M*,*M*)-Z_{st}-CMBID-2H^{2+}**	**-**	-2219.462386	0	100
**(*S*,*S*)-(*M*,*M*)-E_{st}-CMBID-2H^{2+}**	**-**	-2219.457552	0	100

a. The populations were estimated at 298.15 K by Boltzmann distribution law based on the Gibbs free energies with the degeneracy of **Conformer-b** set as 2.

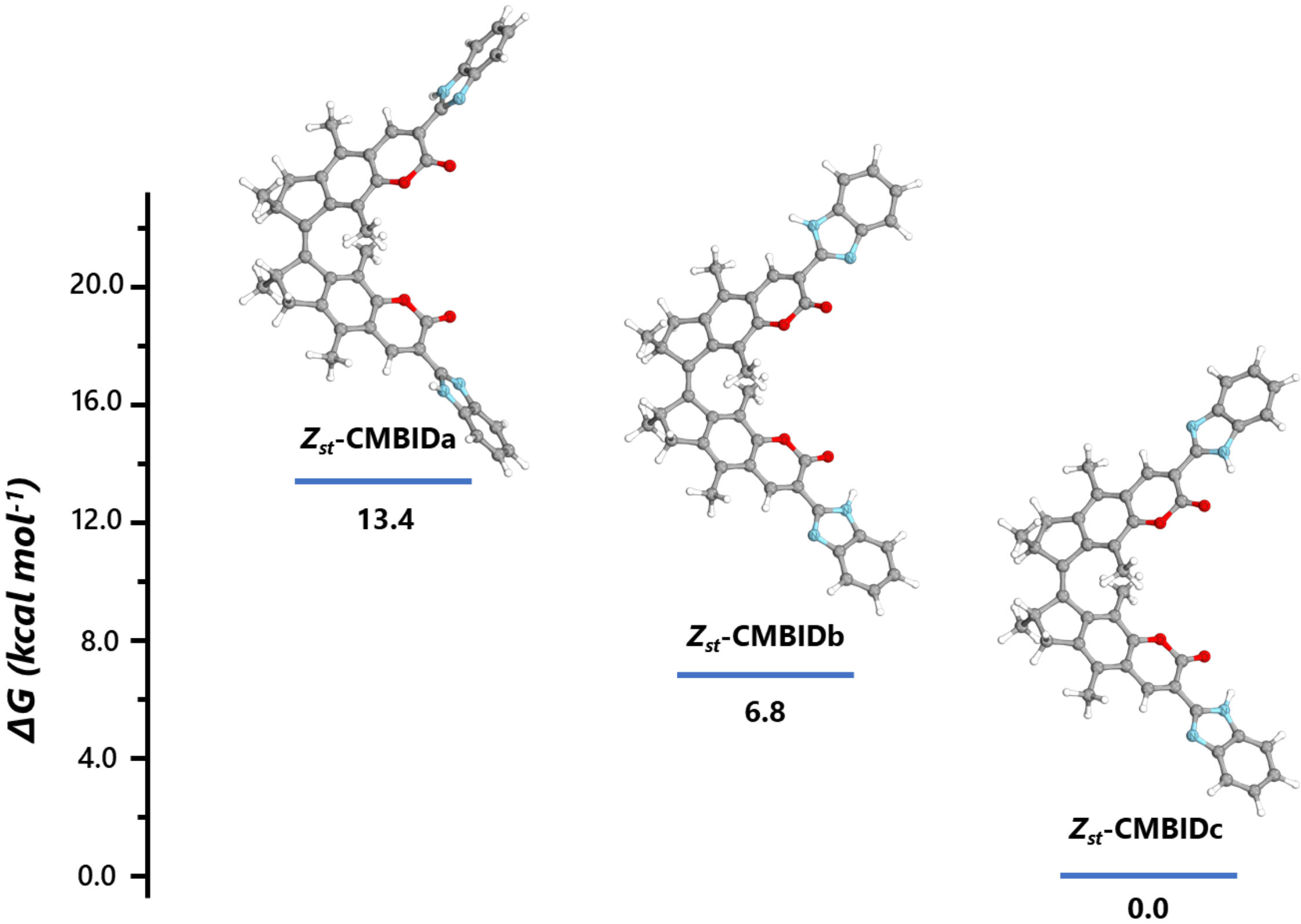

Figure S68. Optimized geometries of different conformers with free energy diagram of **(*S*,*S*)-(*M*,*M*)-Z_{st}-CMBID**, color codes: *O* = red; *N* = blue, respectively.

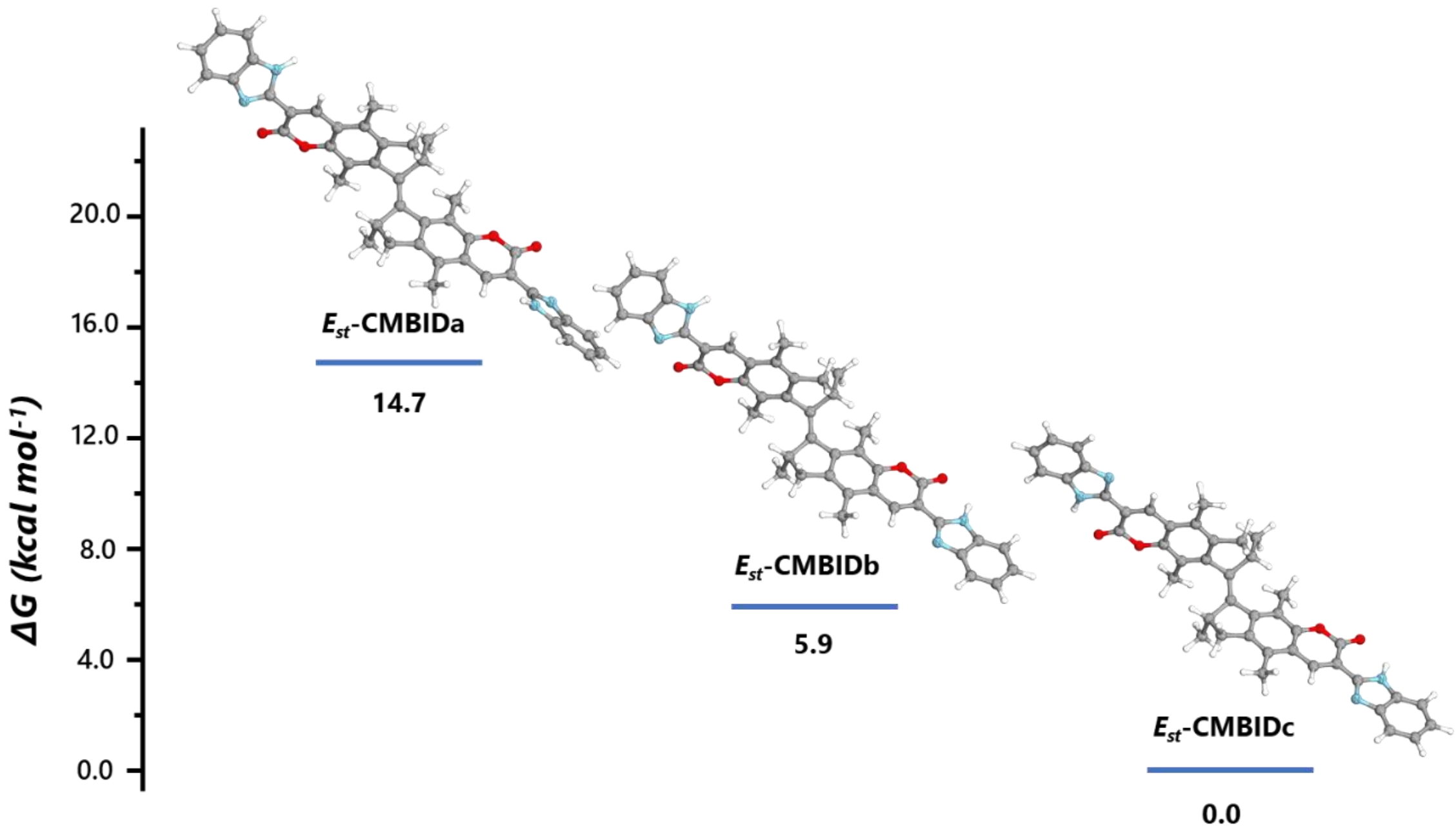

Figure S69. Optimized geometries of different conformers with free energy diagram of **(*S*,*S*)-(*M*,*M*)-E_{st}-CMBID**, color codes: *O* = red; *N* = blue, respectively.

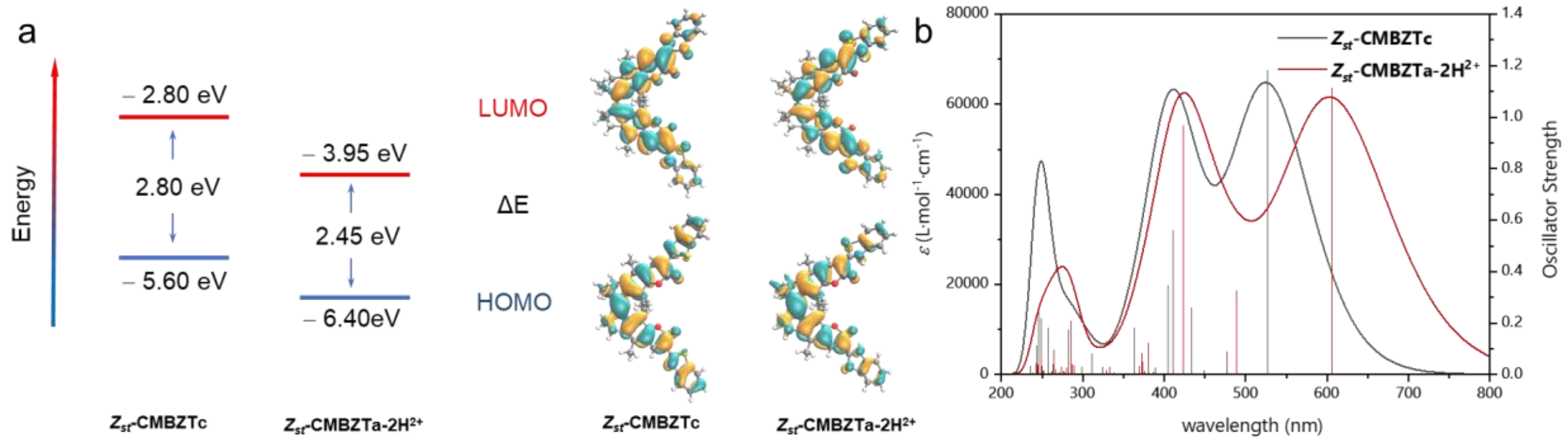

Figure S70. (a). The frontier molecular orbitals and energy gaps and of **(*S*,*S*)-(*M*,*M*)-*Z*$_{st}$-CMBZTc**, and **(*S*,*S*)-(*M*,*M*)-*Z*$_{st}$-CMBZTa-2H^{2+}** based on the optimized ground-state geometries calculated at the B3LYP-D3/Def2-TZVP level. (b). The calculated UV-vis absorption spectra of **(*S*,*S*)-(*M*,*M*)-*Z*$_{st}$-CMBZTc**, and **(*S*,*S*)-(*M*,*M*)-*Z*$_{st}$-CMBZTa-2H^{2+}**. Calculated excitation energies and oscillator strengths f are displayed as sticks for comparison.

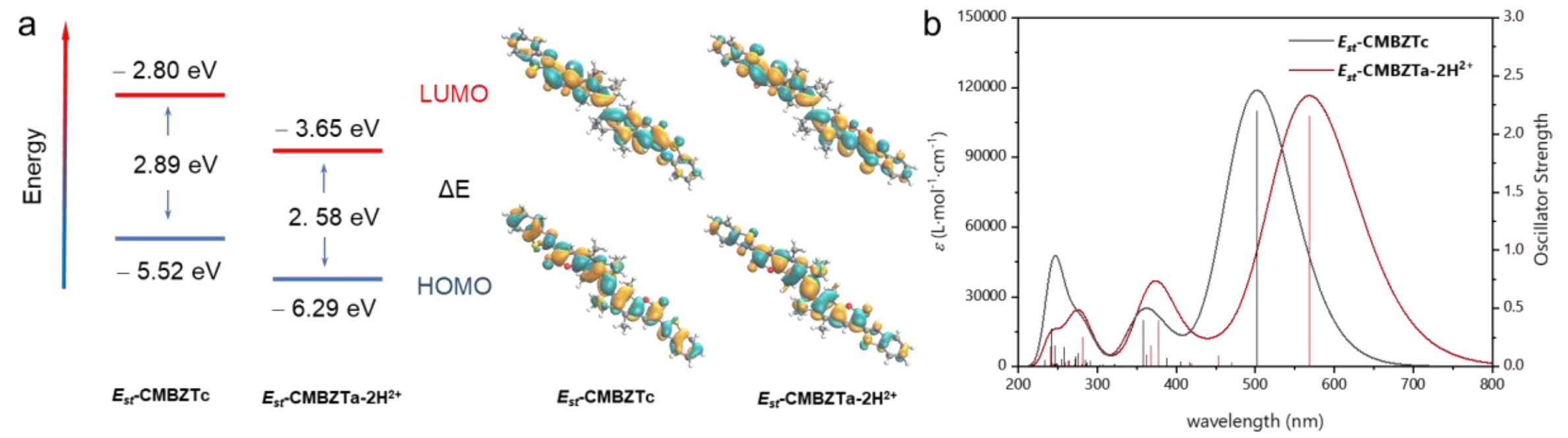

Figure S71. (a). The frontier molecular orbitals and energy gaps and of **(*S*,*S*)-(*M*,*M*)-*E*$_{st}$-CMBZTc**, and **(*S*,*S*)-(*M*,*M*)-*E*$_{st}$-CMBZTa-2H^{2+}** based on the optimized ground-state geometries calculated at the B3LYP-D3/Def2-TZVP level. (b). The calculated UV-vis absorption spectra of **(*S*,*S*)-(*M*,*M*)-*E*$_{st}$-CMBZTc**, and **(*S*,*S*)-(*M*,*M*)-*E*$_{st}$-CMBZTa-2H^{2+}**. Calculated excitation energies and oscillator strengths f are displayed as sticks for comparison.

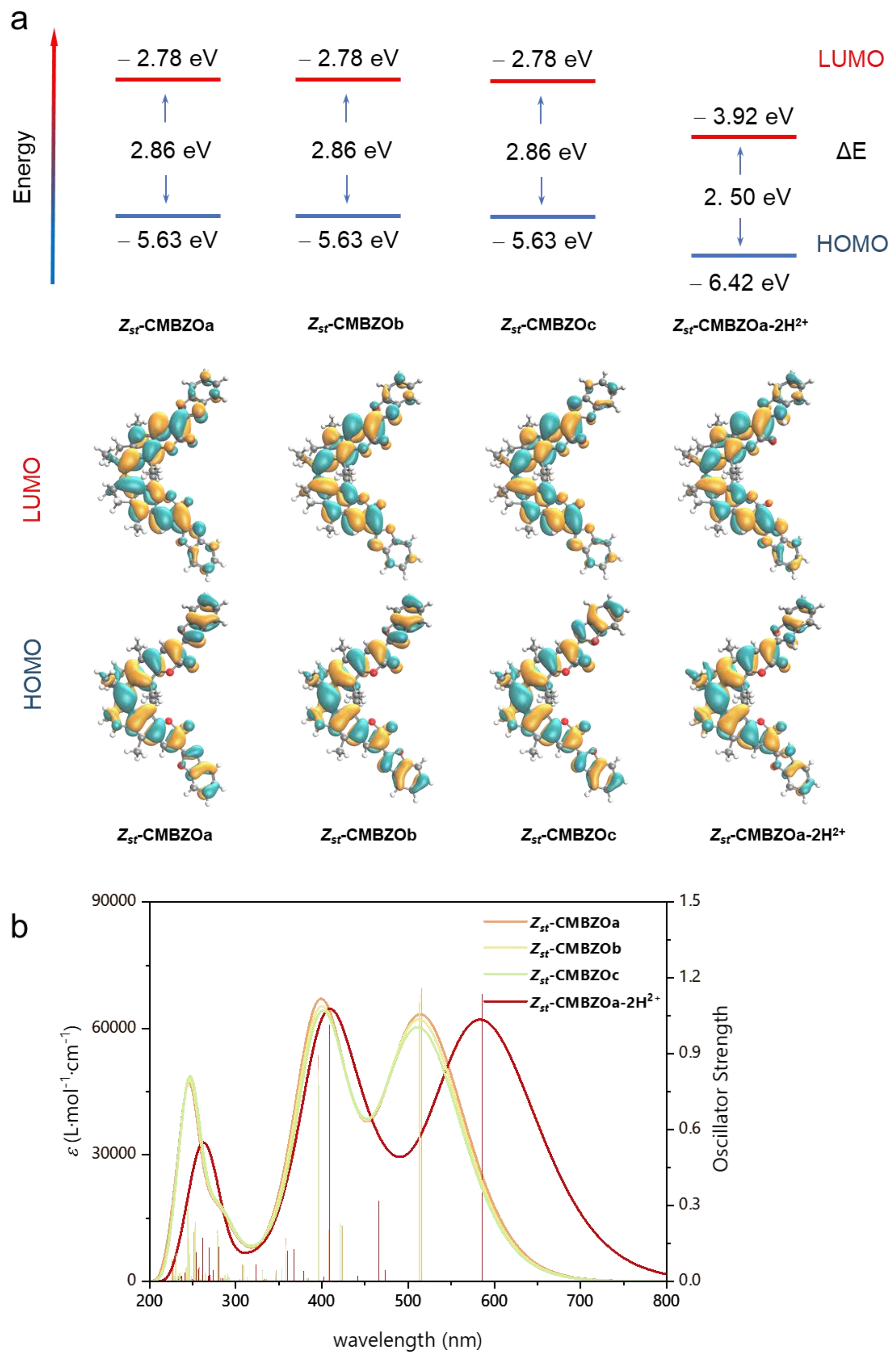

Figure S72. (a). The frontier molecular orbitals and energy gaps and of **(*S*,*S*)-(*M*,*M*)-*Z*$_{st}$-CMBZOa-c**, and **(*S*,*S*)-(*M*,*M*)-*Z*$_{st}$-CMBZOa-2H^{2+}** based on the optimized ground-state geometries calculated at the B3LYP-D3/Def2-TZVP level. (b). The calculated UV-vis absorption spectra of **(*S*,*S*)-(*M*,*M*)-*Z*$_{st}$-CMBZOa-c**, and **(*S*,*S*)-(*M*,*M*)-*Z*$_{st}$-CMBZOa-2H^{2+}**. Calculated excitation energies and oscillator strengths f are displayed as sticks for comparison

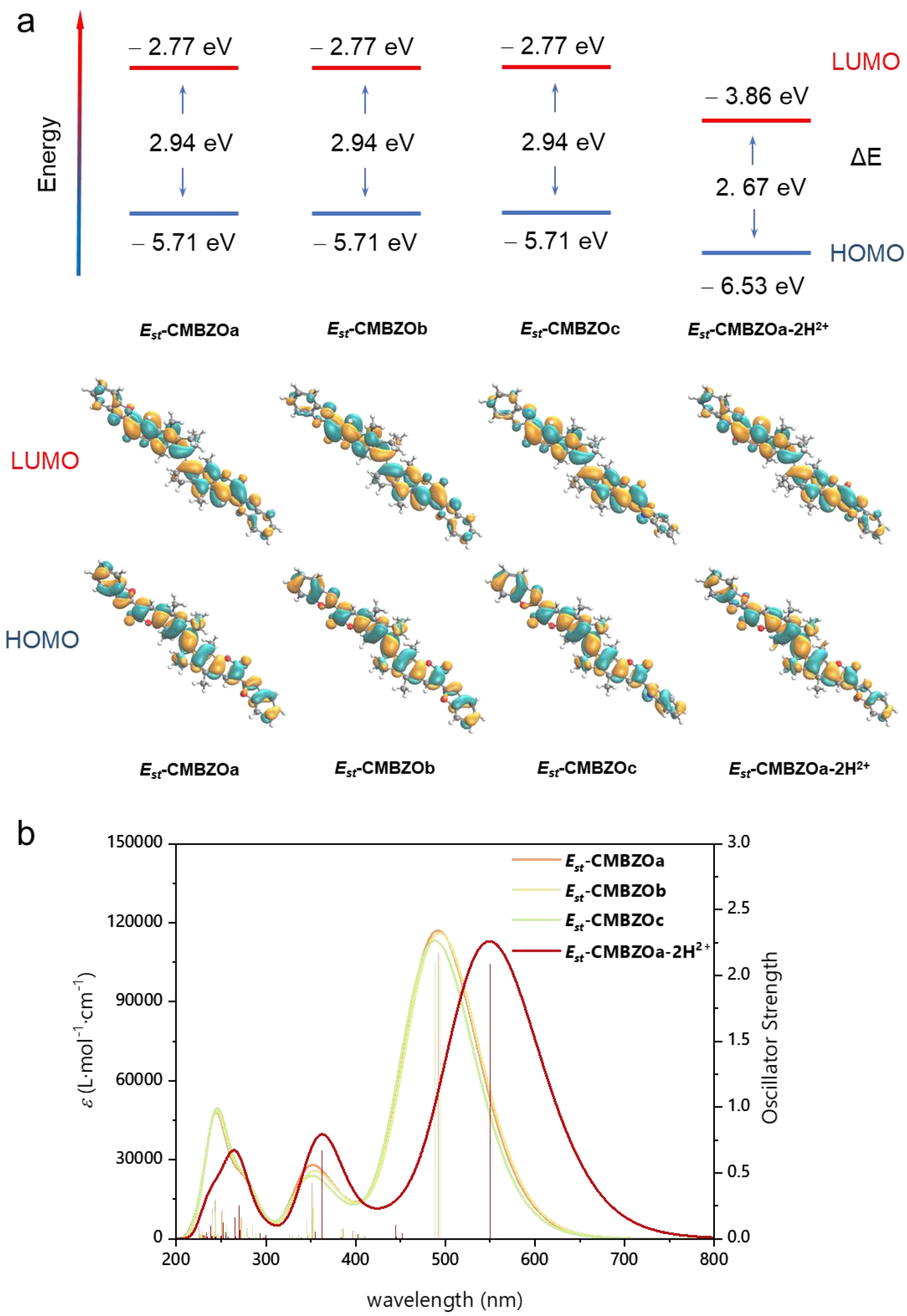

Figure S73. (a). The frontier molecular orbitals and energy gaps and of ***(S,S)*-*(M,M)*-*E**st*-CMBZOa-c**, and ***(S,S)*-*(M,M)*-*E**st*-CMBZOa-2H^{2+}** based on the optimized ground-state geometries calculated at the B3LYP-D3/Def2-TZVP level. (b). The calculated UV-vis absorption spectra of ***(S,S)*-*(M,M)*-*E**st*-CMBZOa-c**, and ***(S,S)*-*(M,M)*-*E**st*-CMBZOa-2H^{2+}**. Calculated excitation energies and oscillator strengths f are displayed as sticks for comparison

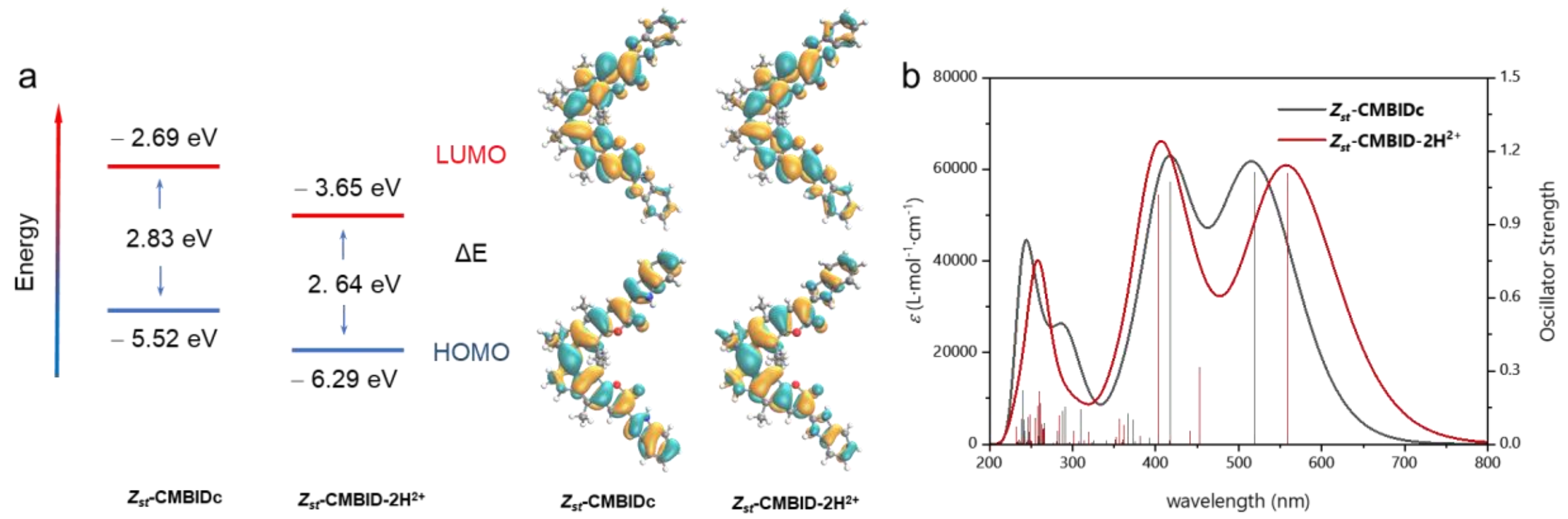

Figure S74. (a). The frontier molecular orbitals and energy gaps and of **(*S*,*S*)-(*M*,*M*)-*Z*$_{st}$-CMBIDc**, and **(*S*,*S*)-(*M*,*M*)-*Z*$_{st}$-CMBID-2H^{2+}** based on the optimized ground-state geometries calculated at the B3LYP-D3/Def2-TZVP level. (b). The calculated UV-vis absorption spectra of **(*S*,*S*)-(*M*,*M*)-*Z*$_{st}$-CMBIDc**, and **(*S*,*S*)-(*M*,*M*)-*Z*$_{st}$-CMBID-2H^{2+}**. Calculated excitation energies and oscillator strengths f are displayed as sticks for comparison.

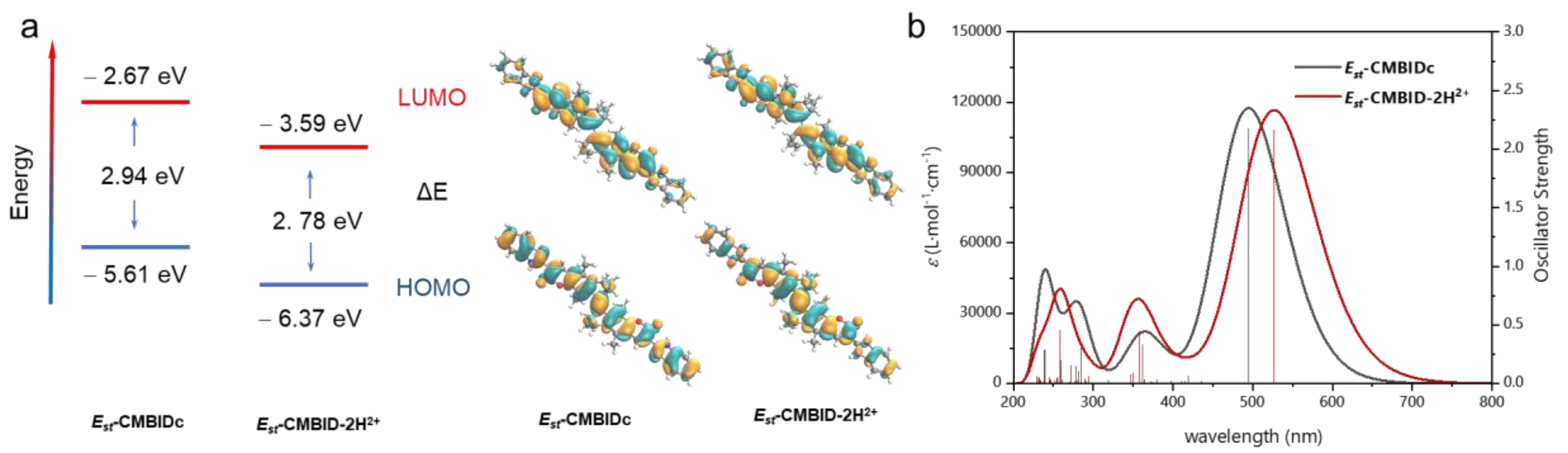

Figure S75. (a). The frontier molecular orbitals and energy gaps and of **(*S*,*S*)-(*M*,*M*)-*E*$_{st}$-CMBIDc**, and **(*S*,*S*)-(*M*,*M*)-*E*$_{st}$-CMBID-2H^{2+}** based on the optimized ground-state geometries calculated at the B3LYP-D3/Def2-TZVP level. (b). The calculated UV-vis absorption spectra of **(*S*,*S*)-(*M*,*M*)-*E*$_{st}$-CMBIDc**, and **(*S*,*S*)-(*M*,*M*)-*E*$_{st}$-CMBID-2H^{2+}**. Calculated excitation energies and oscillator strengths f are displayed as sticks for comparison.

15. NMR spectra

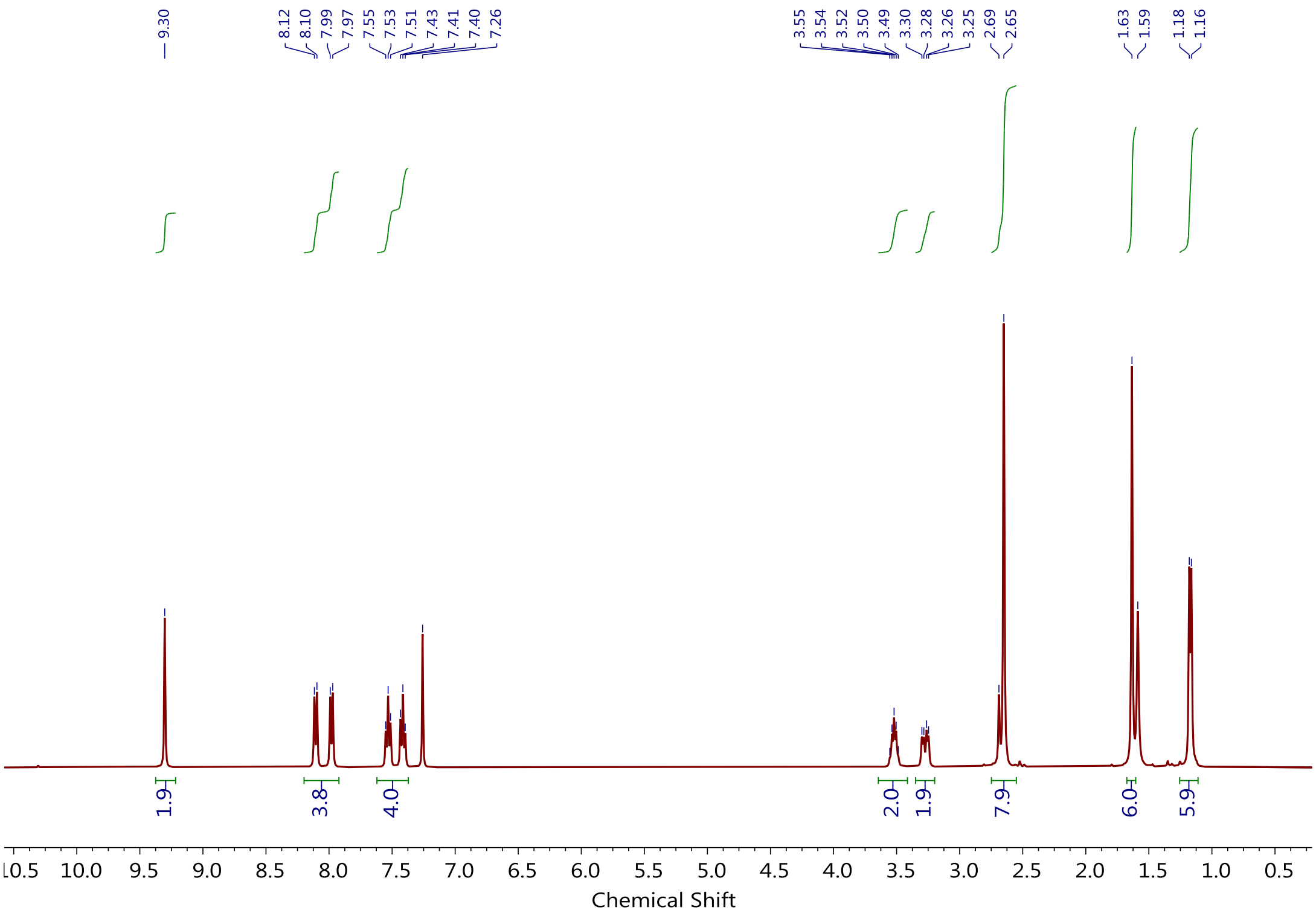

Figure S76. ^{1}H NMR spectrum of ***(S,S)*-(*M,M*)-*Z*$_{st}$-CMBZT** (400 MHz, $CDCl_3$, 298 K).

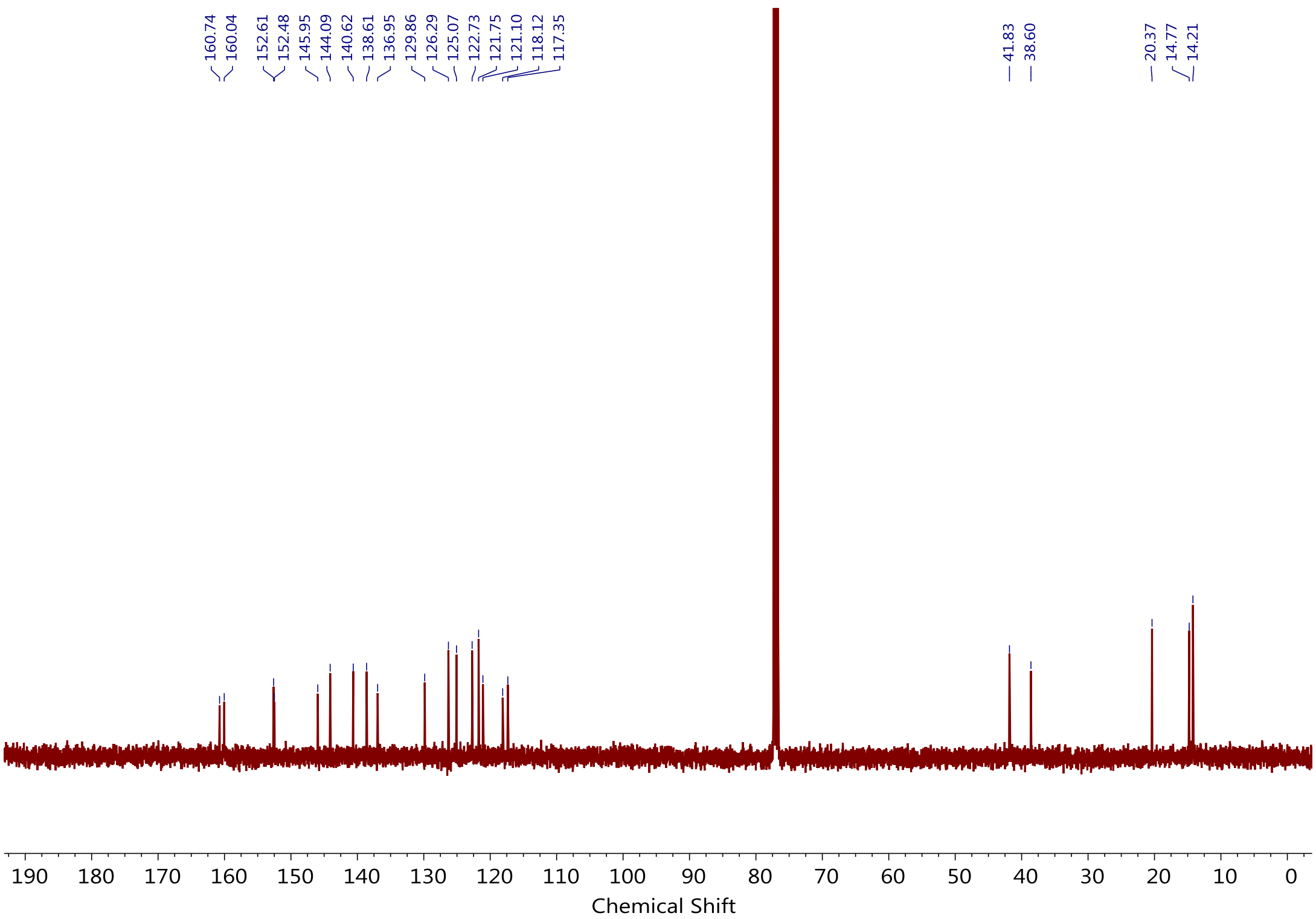

Figure S77. ^{13}C NMR spectrum of ***(S,S)*-(*M,M*)-*Z*$_{st}$-CMBZT** (101 MHz, $CDCl_3$, 298 K).

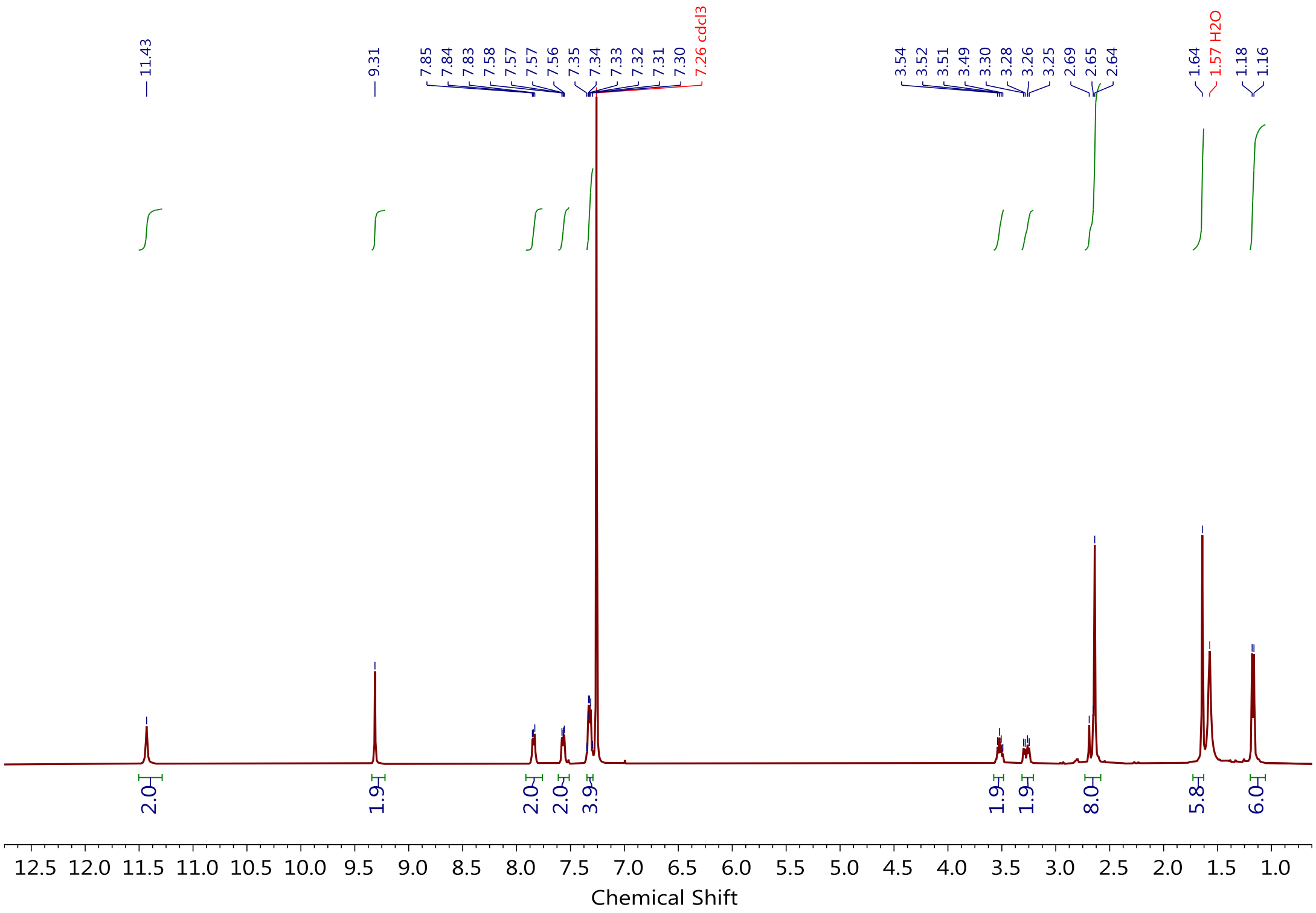

Figure S78. ^{1}H NMR spectrum of ***(S,S)*-*(M,M)*-*Z*$_{\mathbf{st}}$-CMBID** (400 MHz, $CDCl_3$, 298 K).

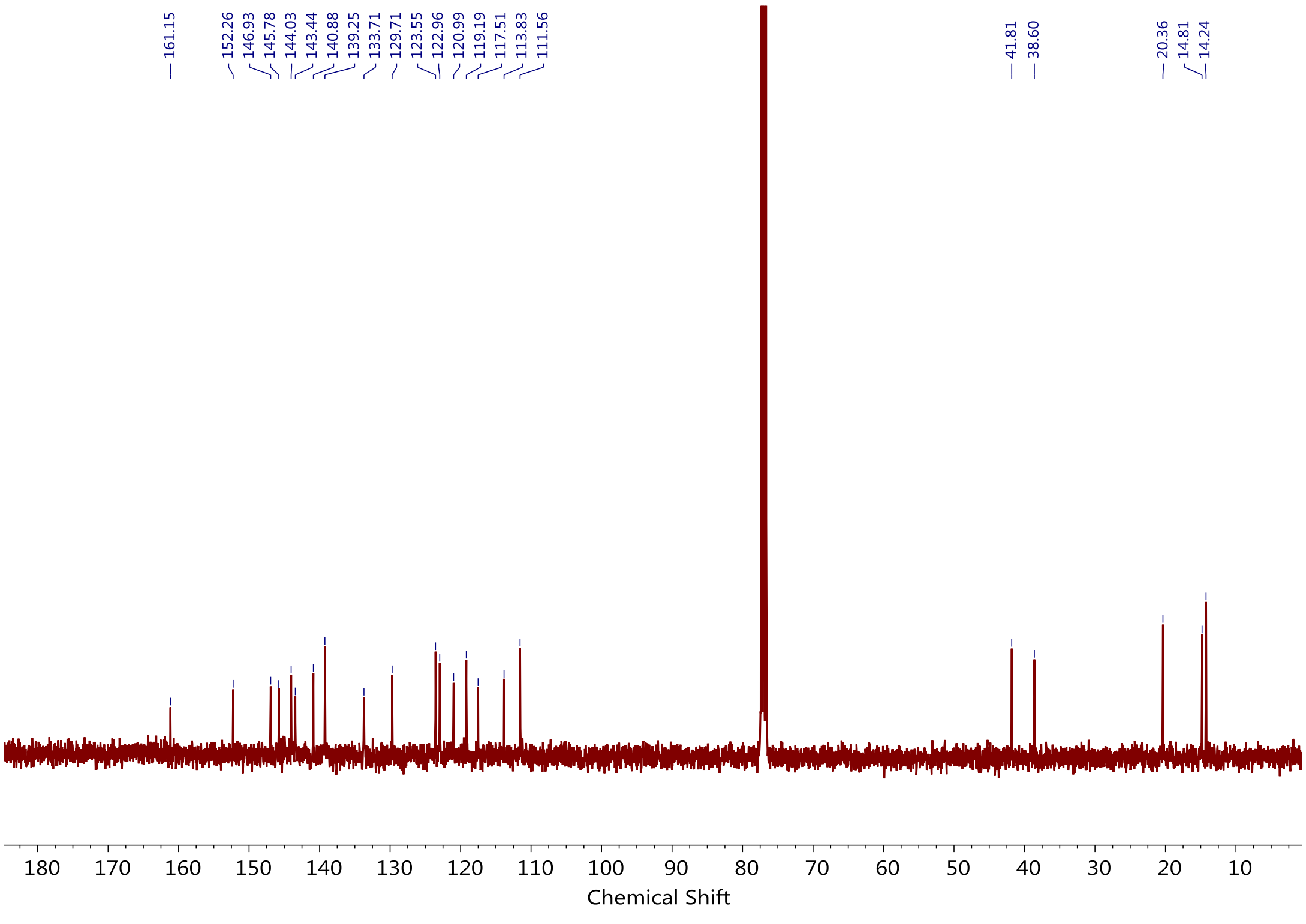

Figure S79. ^{13}C NMR spectrum of ***(S,S)*-*(M,M)*-*Z*$_{\mathbf{st}}$-CMBID** (101 MHz, $CDCl_3$, 298 K).

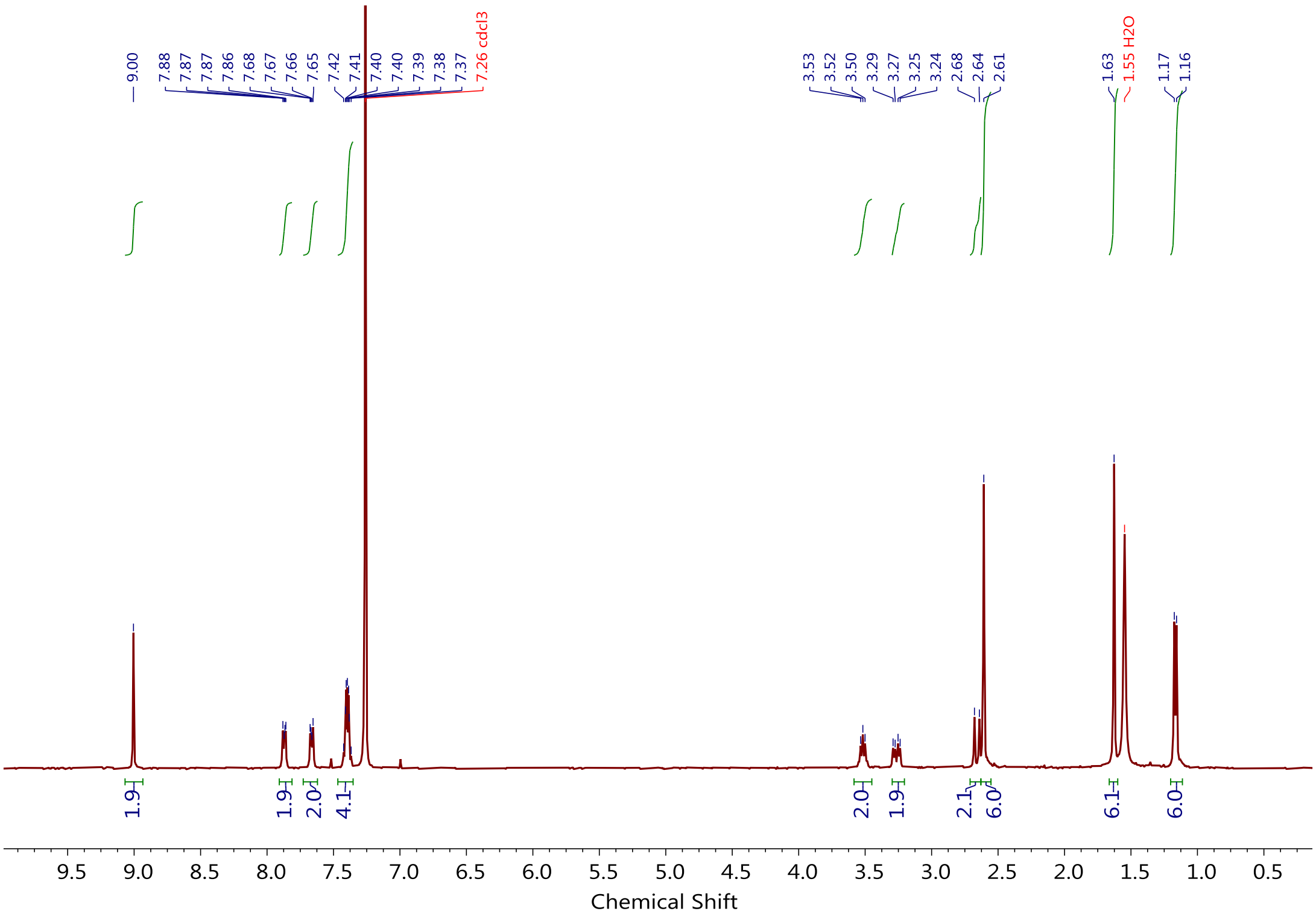

Figure S80. ^{1}H NMR spectrum of ***(S,S)*-*(M,M)*-*Z*$_{st}$-CMBZO** (400 MHz, $CDCl_3$, 298 K).

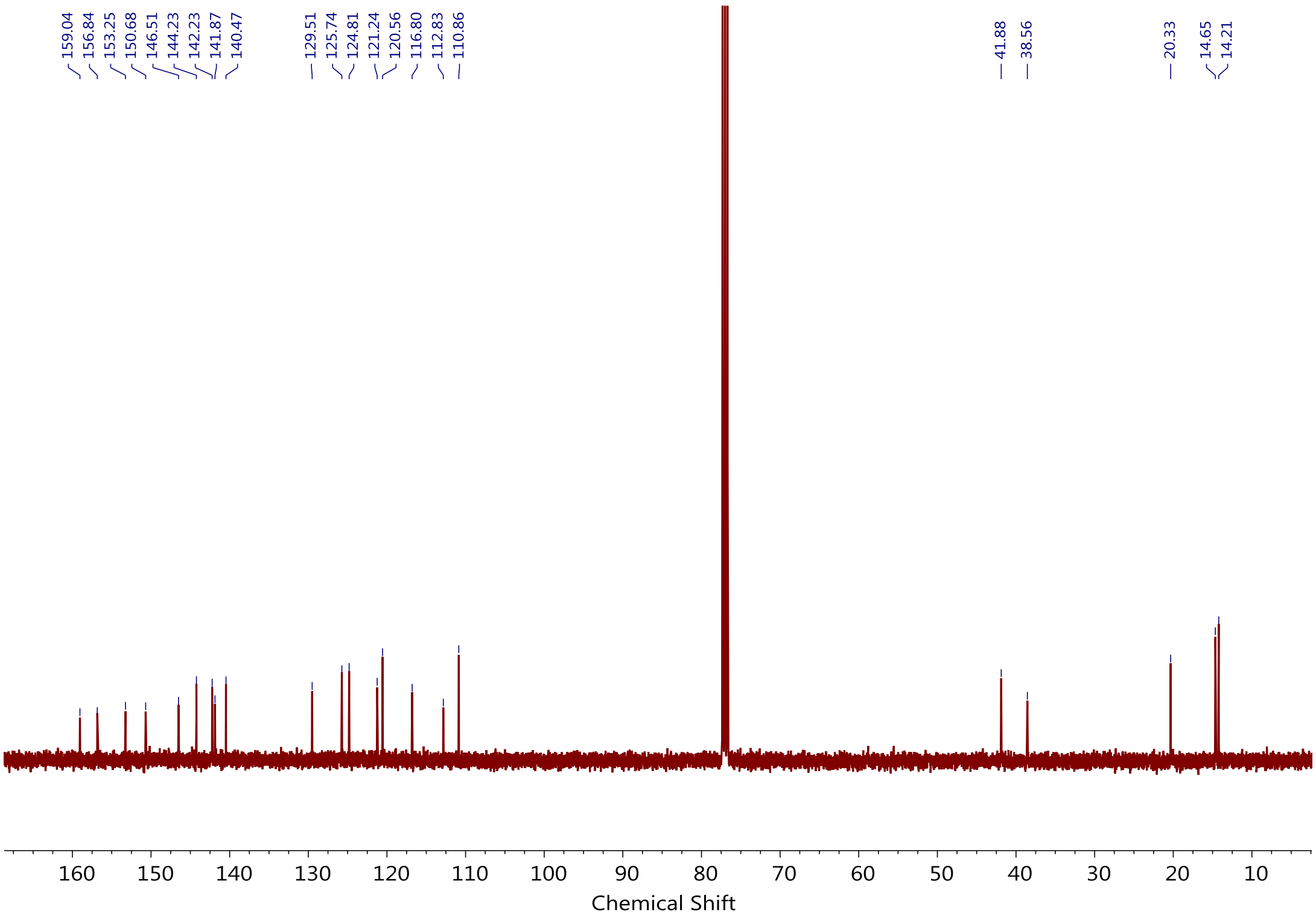

Figure S81. ^{13}C NMR spectrum of ***(S,S)*-*(M,M)*-*Z*$_{st}$-CMBZO** (101 MHz, $CDCl_3$, 298 K).

16. HRMS data of the synthesized compounds

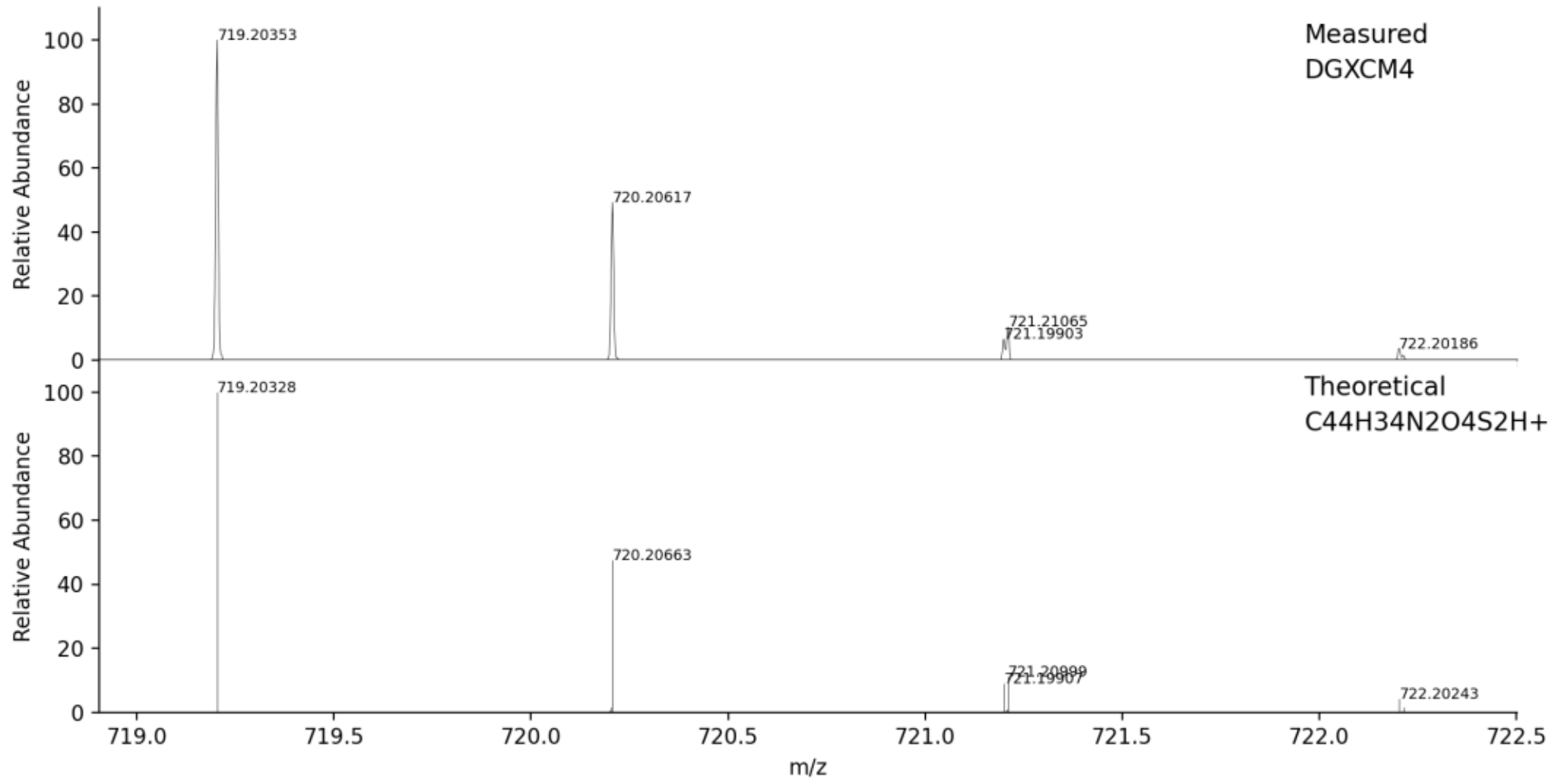

Figure S82. HRMS (ESI) spectra of **(*S*,*S*)-(*M*,*M*)-*Z*$_{st}$-CMBZT** (top: measured, bottom: theoretical).

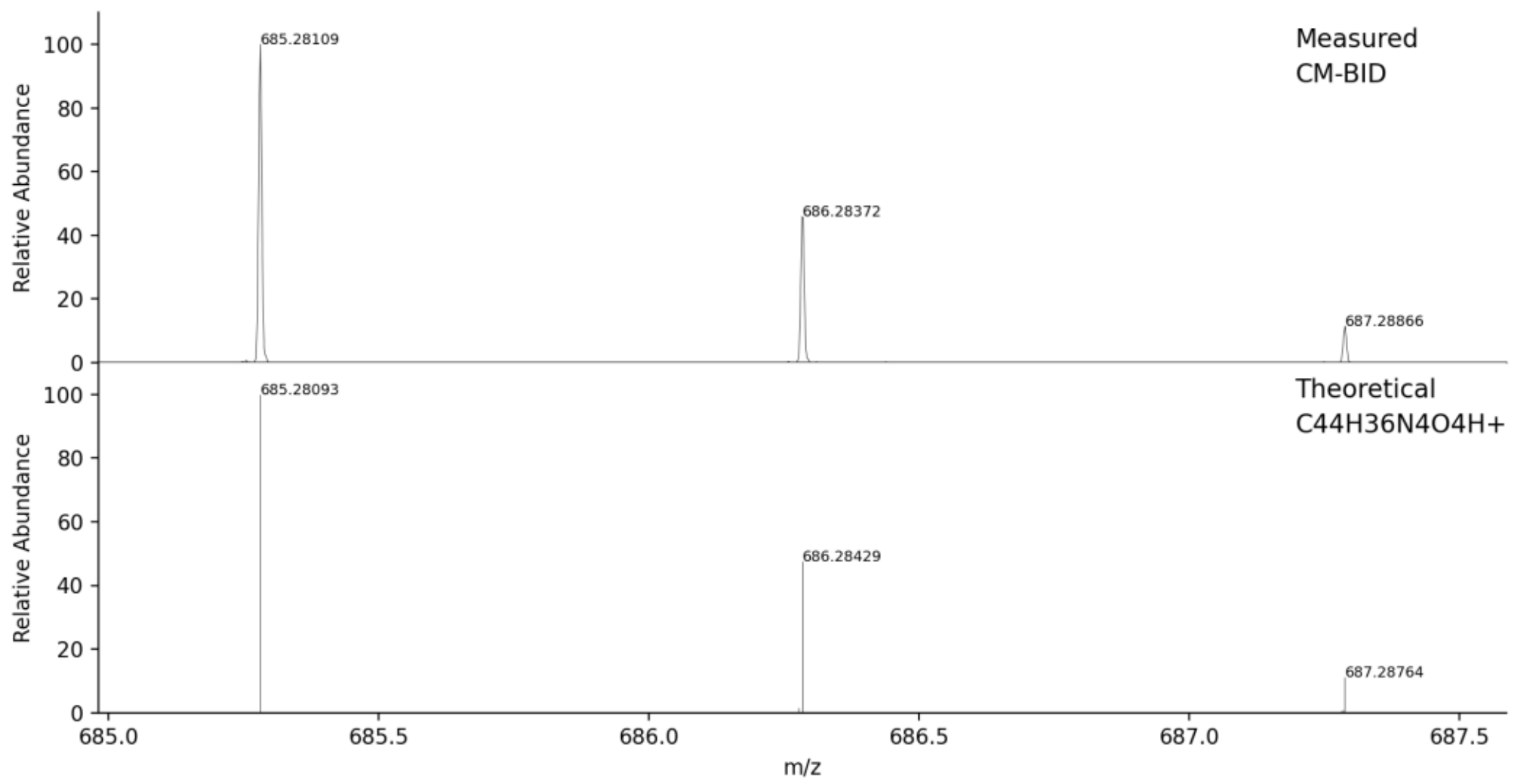

Figure S83. HRMS (ESI) spectra of **(*S*,*S*)-(*M*,*M*)-*Z*$_{st}$-CMBID** (top: measured, bottom: theoretical).

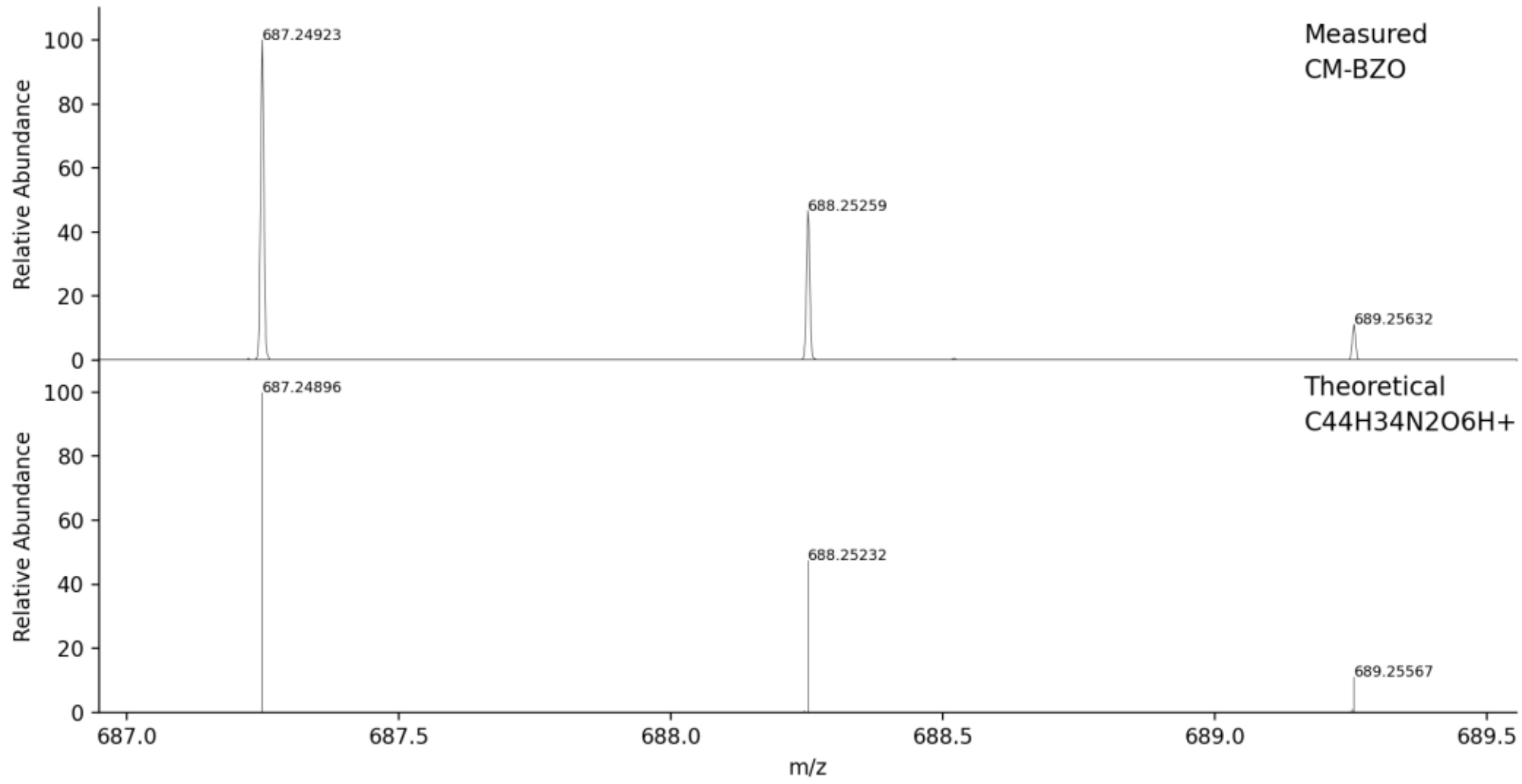

Figure S84. HRMS (ESI) spectra of **(*S*,*S*)-(*M*,*M*)-Z_{st}-CMBZO** (top: measured, bottom: theoretical).

17. References

[1] A. Ryabchun, A. Bobrovsky, *Adv. Opt. Mater.* **2019**, *7*, 1901486.

[2] J. Wang, M. C. A. Stuart, B. L. Feringa, *Angew. Chem. Int. Ed.* **2026**, *65*, e21360.

[3] V. Quesneau, B. Roubinet, P.-Y. Renard, A. Romieu, *Tetrahedron Lett.* **2019**, *60*, 151279.

[4] M. K. J. ter Wiel, R. A. van Delden, A. Meetsma, B. L. Feringa, *J. Am. Chem. Soc.* **2005**, *127*, 14208-14222.

[5] Z. A. Sizova, A. A. Karasev, L. L. Lukatskaya, M. I. Rubtsov, A. O. Doroshenko, *Theor. Exp. Chem.* **2002**, *38*, 168-172.

[6] M. Medved, M. W. H. Hoorens, M. Di Donato, A. D. Laurent, J. Fan, M. Taddei, M. Hilbers, B. L. Feringa, W. J. Buma, W. Szymanski, *Chem. Sci.* **2021**, *12*, 4588-4598.

[7] B. Aydıner, Z. Seferoğlu, *Eur. J. Org. Chem.* **2018**, *2018*, 5921-5934.

[8] Y. W. More, S. D. Padghan, R. S. Bhosale, R. P. Pawar, A. L. Puyad, S. V. Bhosale, S. V. Bhosale, *Sensors* **2018**, *18*, 3433.

[9] K. Stranius, K. Börjesson, *Sci. Rep.* **2017**, *7*, 41145.

[10] H. J. Kuhn, S. E. Braslavsky, R. Schmidt, *Pure Appl. Chem.* **2004**, *76*, 2105-2146.

[11] A. Volker, J. D. Steen, S. Crespi, *Beilstein J. Org. Chem.* **2024**, *20*, 1684-1692.

[12] S. Hoops, S. Sahle, R. Gauges, C. Lee, J. Pahle, N. Simus, M. Singhal, L. Xu, P. Mendes, U. Kummer, *Bioinformatics* **2006**, *22*, 3067-3074.

[13] A. D. Becke, *Phys. Rev. A* **1988**, *38*, 3098-3100.

[14] C. Lee, W. Yang, R. G. Parr, *Phys. Rev. B* **1988**, *37*, 785-789.

[15] A. D. Becke, *J. Chem. Phys.* **1992**, *96*, 2155-2160.

[16] S. Grimme, J. Antony, S. Ehrlich, H. Krieg, *J. Chem. Phys.* **2010**, *132*, 154104.

[17] R. Krishnan, J. S. Binkley, R. Seeger, J. A. Pople, *J. Chem. Phys.* **1980**, *72*, 650-654.

[18] M. J. Frisch, G. W. Trucks, H. B. Schlegel, G. E. Scuseria, M. A. Robb, J. R. Cheeseman, G. Scalmani, V. Barone, G. A. Petersson, H. Nakatsuji, X. Li, M. Caricato, A. V. Marenich, J. Bloino, B. G. Janesko, R. Gomperts, B. Mennucci, H. P. Hratchian, J. V. Ortiz, A. F. Izmaylov, J. L. Sonnenberg, Williams, F. Ding, F. Lipparini, F. Egidi, J. Goings, B. Peng, A. Petrone, T. Henderson, D. Ranasinghe, V. G. Zakrzewski, J. Gao, N. Rega, G. Zheng, W. Liang, M. Hada, M. Ehara, K. Toyota, R. Fukuda, J. Hasegawa, M. Ishida, T. Nakajima, Y. Honda, O. Kitao, H. Nakai, T. Vreven, K. Throssell, J. A. Montgomery Jr., J. E. Peralta, F. Ogliaro, M. J. Bearpark, J. J. Heyd, E. N. Brothers, K. N. Kudin, V. N. Staroverov, T. A. Keith, R. Kobayashi, J. Normand, K. Raghavachari, A. P. Rendell, J. C. Burant, S. S. Iyengar, J. Tomasi, M. Cossi, J. M. Millam, M. Klene, C. Adamo, R. Cammi, J. W. Ochterski, R. L. Martin, K. Morokuma, O. Farkas, J. B. Foresman, D. J. Fox, Wallingford, CT, **2016**.

[19] A. V. Marenich, C. J. Cramer, D. G. Truhlar, *J. Phys. Chem. B.* **2009**, *113*, 6378-6396.

[20] F. Weigend, R. Ahlrichs, *Phys. Chem. Chem. Phys.* **2005**, *7*, 3297-3305.

[21] A. Gilbert, 2.15 ed., IQmol Molecular Viewer, **2015**.
